# Vibrating membrane bioreactors enhance nutrient removal and energy efficiency in municipal wastewater treatment

**DOI:** 10.1038/s41467-026-75747-6

**Published:** 2026-07-20

**Authors:** Weichen Lin, Tao Xue, Haojie Ding, Ziwei Liu, Caiyun Zhang, Kang Xiao, Chunsheng Chen, Xia Huang

**Affiliations:** 1 School of Environment, Beijing Normal University, Beijing, China; 2 State Key Laboratory of Regional Environment and Sustainability, School of Environment, Tsinghua University, Beijing, China; 3 Beijing OriginWater Membrane Technology Co. Ltd., Beijing, China; 4 College of Resources and Environment, University of Chinese Academy of Sciences, Beijing, China; 5 Research and Application Center for Membrane Technology, School of Environment, Tsinghua University, Beijing, China

**Keywords:** Chemical engineering, Pollution remediation

## Abstract

Membrane bioreactors (MBRs) are widely applied in municipal wastewater treatment, yet their broader adoption has been constrained by energy-intensive aeration and membrane fouling. Here, we report approximately 800 consecutive days of full-scale (7500 m^3^ d^−1^) parallel operation of the vibrating MBR (VMBR) and the conventional aerated MBR (AMBR) systems, systematically comparing their pollutant removal performance, long-term filtration stability, membrane fouling mechanisms, and full life cycle environmental impacts. Results showed that the VMBR demonstrated enhanced nutrient removal, lowering effluent total nitrogen by 22% and reducing the chemical dosage for phosphorus removal by 40% compared with the AMBR. It also exhibited enhanced fouling resistance, with a 20% higher average specific flux than that of the AMBR throughout the operation. Mechanism analysis revealed that the VMBR generated effective, uniform shear at the membrane surface while preserving the sludge floc structure, thereby mitigating membrane fouling. Consequently, the VMBR reduced specific energy consumption for fouling control by 75% and the overall carbon footprint by 30%. We validate that VMBRs represent a scalable, energy-efficient and sustainable technology for future wastewater treatment.

## Abstract (web summary)

This study demonstrates that vibrating membrane bioreactors outperform conventional aerated systems in fouling control, energy consumption, and chemical use for more sustainable wastewater treatment, based on nearly 800 days of full-scale operation.

## Introduction

The growing freshwater scarcity, intensified by climate change and urbanization, underscores the need for wastewater reclamation and reuse^1–3^. Membrane bioreactors (MBRs) have emerged as a key technology that integrates biological degradation with membrane filtration to effectively remove pollutants, pathogens, and nutrients from municipal and industrial wastewaters^4^. Compared with conventional activated sludge processes, the MBR process offers distinct advantages, including a significantly reduced footprint, superior effluent quality, and lower excess sludge production resulting from extended sludge retention time^5,6^.

However, membrane fouling remains a major barrier to its broader application, leading to increased operational costs, elevated energy consumption, and reduced process sustainability because of frequent chemical cleaning and membrane replacement^7,8^. The prevailing full-scale mitigation strategy employs continuous aeration to generate scouring shear, a configuration known as an aerated membrane bioreactor (AMBR)^9^. Nevertheless, this approach is energy intensive, with aeration for fouling control typically accounting for 35–50% of the plant’s total energy demand, resulting in substantial operational costs^10,11^. Moreover, the hydrodynamic environment created by aeration is often heterogeneous, leading to uneven fouling distribution and localized clogging^12,13^. In addition, the high dissolved oxygen (DO) concentrations in AMBR membrane tanks can adversely affect biological nutrient removal performance by introducing oxygen into anaerobic and anoxic zones via sludge recirculation^14,15^.

In recent years, alternative fouling control strategies have been explored to address these limitations. One promising technology is vibrating membrane bioreactors (VMBRs), which employ mechanical vibration of the membrane stacks to induce shear at the membrane‒liquid interface^16^. This method decouples fouling control from aeration, thereby eliminating the need for air sparging. Early laboratory studies revealed that combining transverse vibration with axial vibration approximately doubled the critical flux^17^ and identified the vibration frequency as the most influential factor in fouling control^18,19^. Recent laboratory-scale investigations utilizing flat-sheet MBRs compared vertical reciprocating vibration with traditional aeration, confirming that the vibrational mode offered better fouling control, with energy savings of up to 78.5%^20,21^. At the pilot scale of 20–50 m^3^ d^−1^, VMBRs enhanced nitrogen removal, with approximately 3.3 mg  L^−1^ lower total nitrogen (TN) in the effluent and significant energy savings of more than 60% compared with conventional aerated systems^22–24^. Pilot plants across scales of 100 and 1000 m^3^ d^−1^ have consistently confirmed the energy efficiency of VMBRs during more than eight months of operation^25,26^.

While these laboratory and pilot-scale studies preliminarily show the potential of VMBRs for pollutant removal, fouling control, and energy efficiency, they are limited by their scale and often evaluate MBRs as standalone units decoupled from the full biological process train. Consequently, significant knowledge gaps persist at the intersection of engineering performance and the underlying science: (1) What are the core biochemical pathways through which VMBRs enhance nitrogen and phosphorus removal throughout the entire process? (2) How can fouling behavior be quantitatively linked to the distinct hydrodynamic environments created by vibration versus aeration, including their effects on floc structure and foulant release? (3) Can a full-scale comparison support the development of a mechanistic, shear-based theory for fouling control in MBR systems? Therefore, a definitive, side-by-side demonstration at full scale, within a complete biological treatment train, is critical to confirm these benefits and elucidate the underlying mechanisms governing performance at scale.

To address these research gaps, we conduct the first comprehensive and long-term parallel evaluation of full-scale AMBR and VMBR systems. By operating two 7500 m^3^ d^−1^ trains and treating identical wastewater over more than two years (~800 days), we systematically link the macroscale performance to microscale mechanisms. Our investigation integrates three aspects: (1) evaluating nutrient removal performance and characterizing functional microbial communities to elucidate biochemical pathways; (2) analyzing fouling development, sludge properties, and floc morphology to quantify the effects of the shear regime; and (3) combining computational fluid dynamics (CFD) simulations with energy analysis to establish a mechanistic understanding of shear distribution and fouling control. By unifying process engineering with microbial ecology and hydrodynamics, this work provides not only a full-scale performance benchmark but also a science-based foundation for the design and optimization of energy-efficient MBR systems.

## Results

### Removal performance of nutrients

**Fig. 1:**
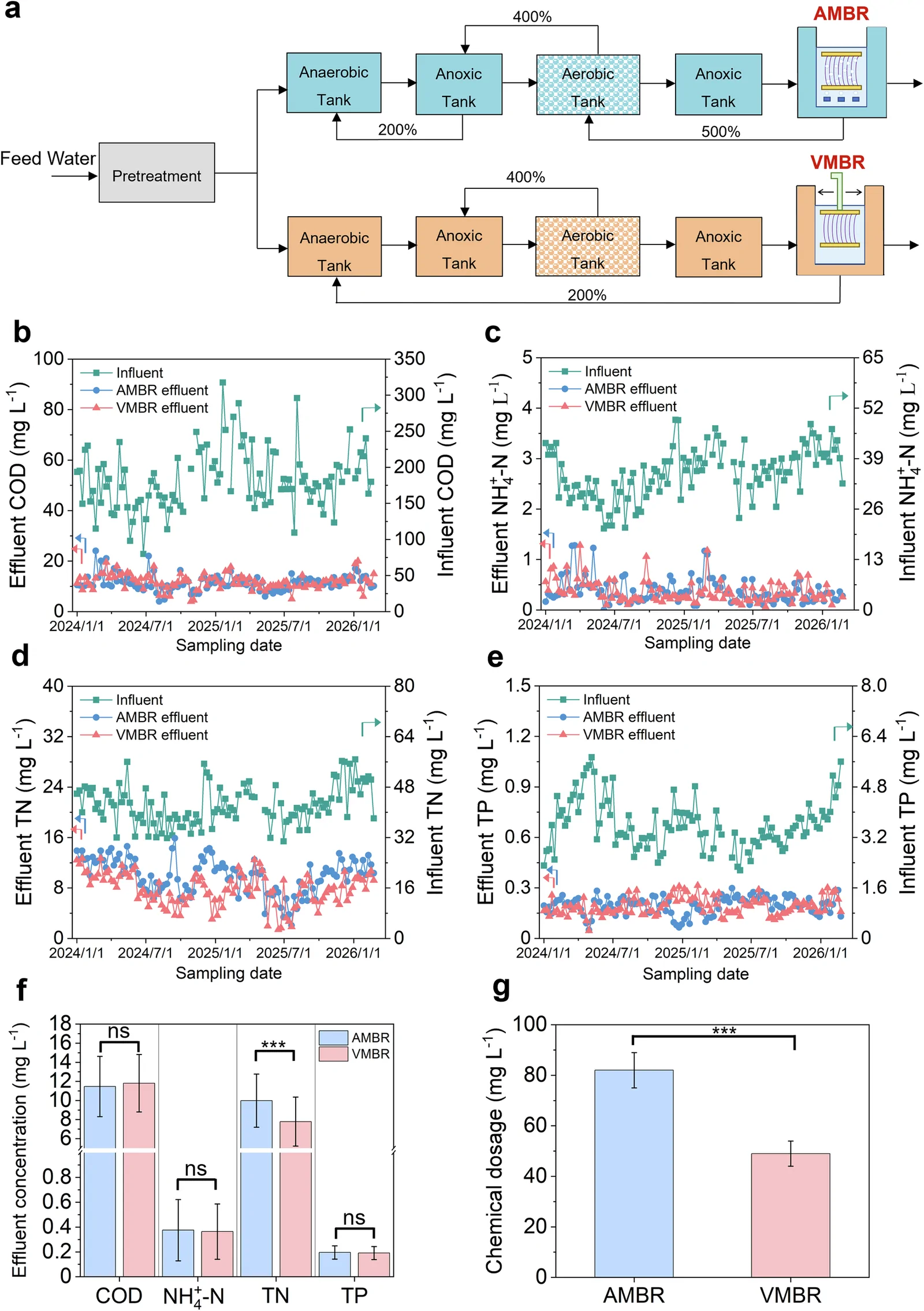
Process flow diagrams and pollutant removal performance of the aerated membrane bioreactor (AMBR) and the vibrating membrane bioreactor (VMBR). **a** Schematic of the AMBR and VMBR processes. Weekly monitoring of influent and effluent quality of municipal wastewater treated by the AMBR and VMBR over a nearly 800-day operational period: (**b**) COD, (**c**) NH_4_^+^-N, (**d**) TN, and (**e**) TP. **f** Comparison of average effluent quality between the AMBR and VMBR. **g** Chemical dosage (FeCl_3_) for phosphorus removal in AMBR and VMBR systems. The error bars in the figures denote the standard deviation (SD) (*n* ≥ 110), and statistical significance was assessed by a two-tailed Student’s *t* test (*, *p* < 0.05; **, *p* < 0.01; ***, *p* < 0.001; ns, not significant).

A parallel full-scale comparison was conducted between a VMBR and an AMBR train (each with a design capacity of 7500 m^3^ d^−1^) at the same municipal wastewater treatment project. Both systems were configured with identical multistage anaerobic–anoxic–aerobic–anoxic biological treatment trains (Fig. 1a), followed by aerated and vibrating membrane tanks (Supplementary Fig. 1 and Supplementary Movies 1, 2). The two systems differed fundamentally in their membrane fouling control strategies: the AMBR relied on continuous aeration, while the VMBR adopted a horizontal reciprocating vibration. Corresponding to this difference in fouling control, the sludge recirculation pathways were also distinct, driven by the large discrepancy in DO levels in the membrane tanks (7.1 ± 0.9 mg L^−1^ in the AMBR vs. 0.06 ± 0.02 mg L^−1^ in the VMBR; Supplementary Fig. 2). In the AMBR, mixed liquor from the membrane tank wasrecycled to the aerobic tank before internal redistribution to the anaerobic/anoxic zones. In contrast, the anoxic mixed liquor from the VMBR membrane tank  was recirculated directly to the anaerobic tank, which enhanced the overall process efficiency.

As illustrated in Fig. 1b–e, over a continuous monitoring period of ~800 days, the effluent quality of both processes stably met the local discharge standards (COD < 30 mg L^−1^, NH_4_^+^-N < 1.5 mg L^−1^, TN < 15 mg L^−1^, and TP < 0.3 mg L^−1^). The average effluent COD and NH_4_^+^-N concentrations were comparable between the two systems (Fig. 1b–c), with no statistically significant difference (*p* > 0.05; Fig. 1f). A notable difference in TN removal was detected (Fig. 1d). Compared with the AMBR (10.0 ± 2.8 mg L^−1^; 76% removal), the VMBR achieved a significantly lower effluent TN concentration (7.8 ± 2.6 mg L^−1^; 82% removal), corresponding to a 22% reduction in effluent TN residues (*p* < 0.05; Fig. 1f). Although both systems achieved similar effluent TP levels ( ~ 0.2 mg L^−1^; Fig. 1e), the chemical dosage (FeCl_3_) required for phosphorus removal was 40% lower in the VMBR (49 ± 5 mg L^−1^) than in the AMBR (82 ± 7 mg L^−1^) (Fig. 1g). This reduction highlights the enhanced biological phosphorus removal capability of VMBRs, an advantage not captured in prior laboratory- and pilot-scale studies focused solely on MBR units^20,25,27^.

The enhanced nitrogen removal and lower chemical usage observed in the VMBR system underscore the critical advantage of decoupling membrane fouling control from aeration. A comparison of nitrate nitrogen removal in individual units between the AMBR and VMBR systems (Supplementary Fig. 3) revealed that although nitrogen removal in the anaerobic tank of the VMBR was lower than that in the AMBR, it was greater in the preanoxic, postanoxic, and membrane tanks. This was attributed to the oxygen-rich mixed liquor in the AMBR, which resulted in a greater reflux of nitrate to the anaerobic tank, where it could compete for and consume more of the high-quality carbon source—volatile fatty acids (VFAs). In the VMBR, lower nitrate reflux allowed influent VFAs to be stored internally, supporting enhanced endogenous denitrification in subsequent anoxic zones. More importantly, the high-DO concentration in the AMBR membrane tank induced endogenous respiration and oxidation, causing a nitrogen release of 2.3 mg L^−1^ (Supplementary Fig. 3). Conversely, the DO concentration in the VMBR membrane tank remained below 0.1 mg L^−1^ (Supplementary Fig. 2), allowing endogenous denitrification to continue and removing 1.5 mg L^−1^ of nitrogen. This finding indicates that the VMBR system achieved a net 3.8 mg L^−1^ nitrogen removal advantage within the membrane tank alone (Supplementary Fig. 3).

Notably, the differences in DO environment and recycle strategy between the two systems are not independent variables, but endogenous outcomes of the distinct fouling control technologies. The anoxic environment in the VMBR membrane tank directly contributed a 3.8 mg L^−1^ TN removal advantage via sustained endogenous denitrification, while the optimized recycle regime enabled by this anoxic environment accounted for the remaining TN removal benefit and 40% reduction in chemical dosage for phosphorus removal.

Analysis of TP removal pathways revealed different behaviors between the two systems (Supplementary Fig. 4). For the AMBR, membrane tank aeration caused high nitrate/DO carryover into the anaerobic tank, leading to 6.7 mg L^−1^ of nitrate removal that depleted VFAs and disrupted the anaerobic environment required for phosphate-accumulating organisms (PAOs) metabolism (Supplementary Fig. 3). In contrast, vibration-based fouling control in the VMBR maintained an anoxic membrane tank (DO < 0.1 mg L^−1^), limiting anaerobic nitrate removal to 3.0 mg L^−1^, preserving VFAs and sustaining the strict anaerobic conditions prerequisite for the enrichment of PAOs and denitrifying PAOs (DPAOs). The VMBR system resulted in significant anaerobic phosphorus release (8 mg L^−1^), indicating robust activity of these enriched populations, followed by substantial biological uptake in the preanoxic and aerobic zones (4.9 mg L^−1^ and 6.4 mg L^−1^, respectively), while negligible biological phosphorus removal in the AMBR led to near-total reliance on chemical precipitation (Supplementary Fig. 4).

The results of the microbial community analysis provide a clear functional explanation (Supplementary Fig. 5). The VMBR was enriched in PAOs and DPAOs (total abundance of 4.3% in the VMBR vs. 1.9% in the AMBR), particularly the key genus *Dechloromonas*, which facilitated simultaneous phosphorus uptake and nitrate reduction. Although the competing glycogen-accumulating organism *Candidatus Competibacter* was more abundant in the VMBR (0.8% in the VMBR vs. 0.4% in the AMBR), the overwhelming dominance of PAOs ensured efficient carbon utilization for biological phosphorus removal. This PAO/DPAO activity was synergistically supported by a stronger nitrifying community, including *Nitrospira* (5.4% in the VMBR vs. 4.3% in the AMBR), which secured a reliable nitrate supply, and a more abundant and diverse denitrifying guild (total 10.4% vs. 9.0%) featuring genera such as *Denitratisoma* (1.8%) for effective nitrate reduction. In contrast, the microbial consortium in the AMBR, characterized by a less developed PAO/DPAO population, resulted in less efficient biological phosphorus removal, directly explaining its greater dependence on chemical supplementation.

### Operational stability and fouling behavior

**Fig. 2:**
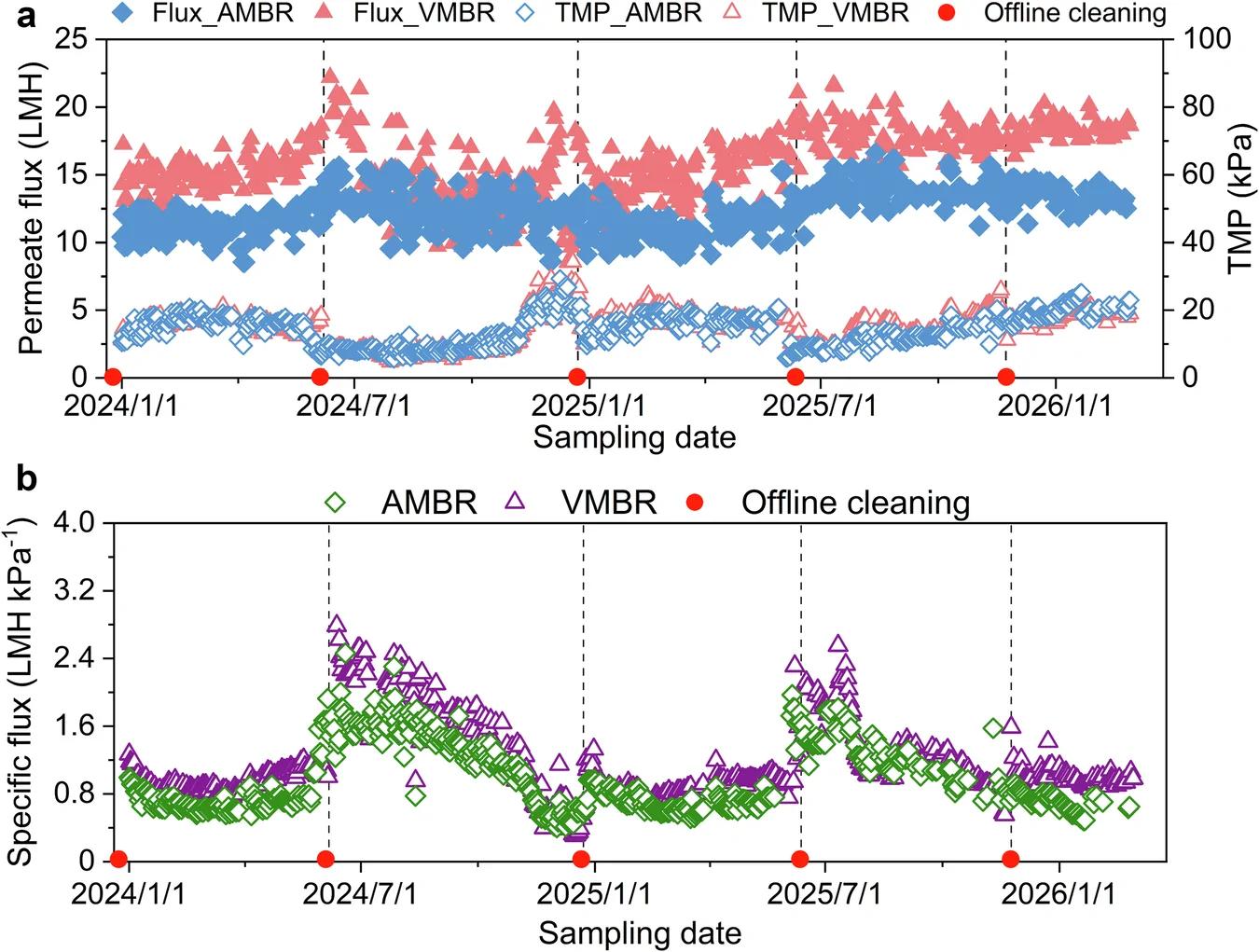
Comparison of operational stability between AMBR and VMBR over a nearly 800-day operational period. **a** Temporal variations of permeate flux and transmembrane pressure (TMP). **b** Temporal variation of specific flux (ratio of permeate flux to TMP). Vertical dashed lines indicate offline cleaning events for both systems.

Figure 2 compares the long-term operational stability of the full-scale AMBR and VMBR systems over the ~800-day operational period. As illustrated in Fig. 2a, the two systems exhibited similar transmembrane pressure (TMP) variation patterns, with TMP dropping sharply after each offline cleaning event and rising gradually during sustained operation, and both maintained stable TMP levels below 30 kPa throughout the monitoring period. However, the permeate flux of the AMBR was consistently lower than that of the VMBR throughout most of the operation cycles. Over the full monitoring period, the AMBR recorded an average permeate flux of 12.3 ± 1.5 L m^−2^ h^−1^ (LMH), while the VMBR maintained a significantly higher average flux of 15.7 ± 2.3 LMH (*p* < 0.001, Supplementary Fig. 6a).

The specific flux, defined as the ratio of permeate flux to TMP (a metric for membrane permeability and fouling propensity), is presented in Fig. 2b. Over the full monitoring period, the VMBR maintained higher specific flux values for the majority of the operation cycle (Fig. 2b), and achieved an average specific flux 20% higher than the conventional AMBR, with the difference statistically significant (*p* < 0.001, Supplementary Fig. 6b). These results confirm that the vibration-based fouling control strategy delivers improved long-term filtration performance in full-scale applications. Even under identical offline cleaning regimes, the VMBR preserved better membrane permeability, mitigated fouling progression, and exhibited more robust operational stability than the conventional AMBR.

To elucidate the intrinsic mechanisms underlying the long-term filtration stability of the VMBR system, we conducted a systematic characterization of membrane samples collected at 30 (M1), 60 (M2), and 90 days (M3) within the first complete operation cycle after the first offline cleaning. Scanning electron microscopy (SEM) images of the fouling layers revealed that both systems experienced combined organic and biological fouling, with sporadic inorganic scaling (Supplementary Fig. 7). Energy-dispersive X-ray (EDX) analysis indicated that the AMBR was more prone to inorganic and organo-inorganic composite fouling. Specifically, the AMBR foulant contains higher proportions of inorganic fouling-related elements (e.g., Si: 1.55%, Al: 0.60%, Ca: 0.60%, Fe: 0.80%) alongside a higher organic carbon content (49.75%), while the VMBR foulant showed significantly reduced inorganic element levels (e.g., Si: 0.20%, Al: 0.20%, Ca: 0.72%, Fe: 0.37%) and a comparable organic carbon fraction (45.21%), confirming less severe inorganic and composite fouling (Supplementary Fig. 8). Furthermore, the total organic carbon (TOC) and adenosine triphosphate (ATP) levels were consistently higher in the AMBR after the initial stage (Supplementary Fig. 9a, b), reflecting relatively more severe organic and biological fouling.

Analysis of organic foulant composition based on the NaClO elution method revealed a temporal shift from proteins and polysaccharides in the initial fouling layer (M1) toward a higher proportion of recalcitrant humic acids in later stages (M2 and M3) (Supplementary Fig. 9c–e). This transition could be attributed to the biodegradation of readily adsorbable biopolymers and the gradual accumulation of resistant humic substances^28^. Quantitative analysis and excitation–emission matrix (EEM) fluorescence spectra collectively indicated a higher degree of organic fouling in the AMBR system relative to the VMBR system (Supplementary Fig. 10).

### Physicochemical properties of membrane tank mixed liquor

To elucidate the mechanisms underlying the divergent nutrient removal and fouling behaviors, the physicochemical properties of the mixed liquor in the AMBR and VMBR membrane tanks were analyzed. Fundamental characteristics differed distinctly between the two systems. The pH in the VMBR system was consistently slightly higher (Supplementary Fig. 11), which was attributed to proton consumption during denitrification in its anoxic environment. The mixed liquor viscosity increased over time in both systems (Supplementary Fig. 12), which correlated with increasing sludge concentration and extracellular polymeric substance (EPS) accumulation^29^. The DO concentration in the AMBR membrane tank was consistently high (>6.5 mg L^−1^), whereas it was close to zero in the VMBR membrane tank (Supplementary Fig. 13). The persistent high-DO environment in the AMBR tank inhibited the anoxic conditions required for denitrification, limiting the nitrogen removal efficiency. Additionally, intensive aeration shear causes the disintegration of sludge flocs^30^. Conversely, the anaerobic environment in the VMBR supported advanced nitrogen removal and minimized physical floc damage. The VMBR system also sustained higher concentrations of mixed liquor suspended solids (MLSS) and volatile suspended solids (MLVSS), indicating higher biomass retention and treatment efficiency (Supplementary Fig. 14)^31^. Moreover, the lower recirculation ratio (200% in the VMBR vs. 500 % in the AMBR) contributed to this higher biomass concentration. The elevated VSS/SS ratio and reduced EPS content under low-shear conditions further indicated that microbial metabolism was directed more toward proliferation rather than defensive EPS secretion, preserving active biomass for pollutant degradation.

**Fig. 3:**
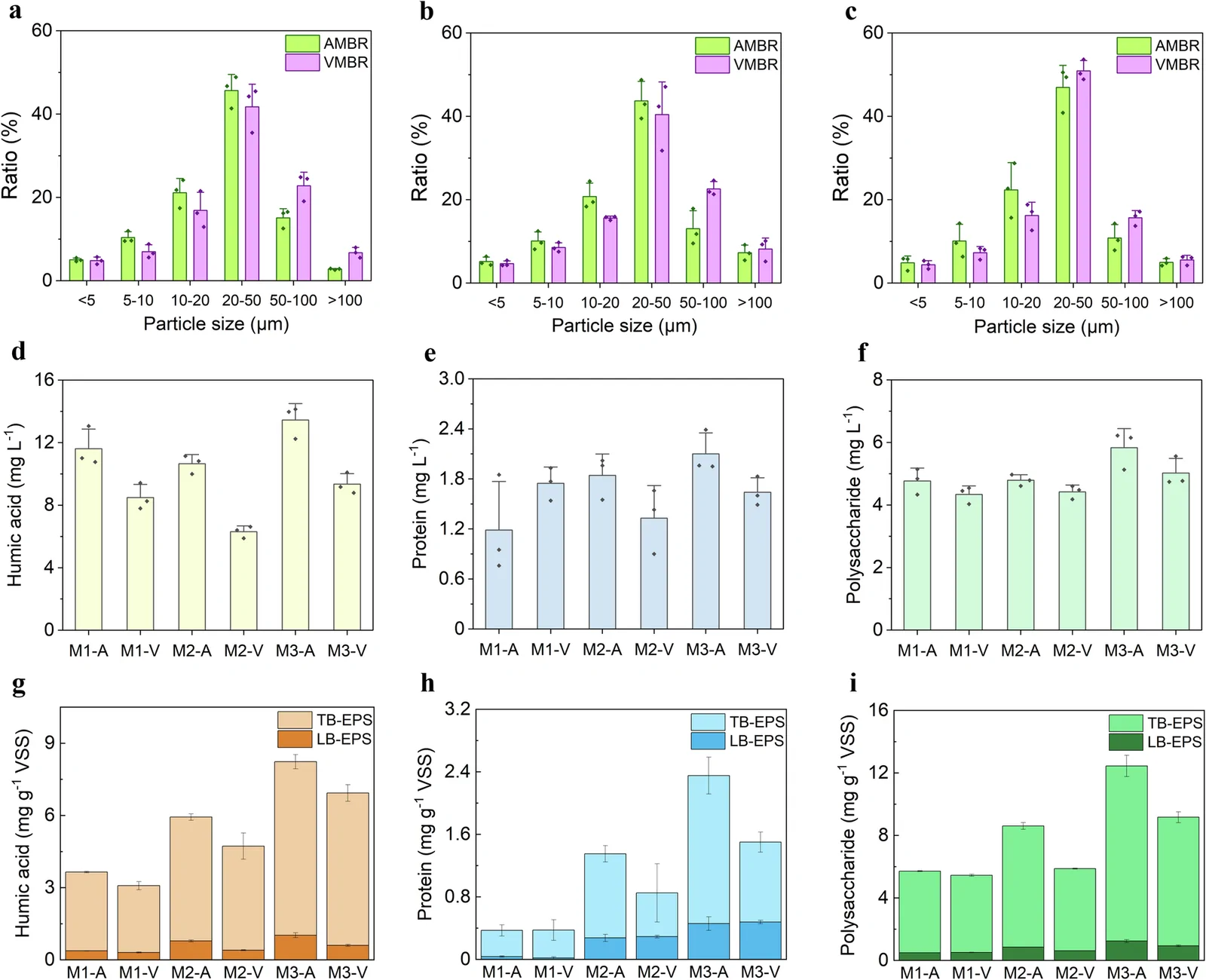
Physicochemical properties of the mixed liquor of the AMBR and VMBR membrane tanks at different stages (M1, M2, and M3). Comparative percentages of particle size fractions in the mixed liquor of the AMBR and VMBR at stages (**a**) M1, (**b**) M2, and (**c**) M3. Comparison of the contents of (**d**) humic acids, (**e**) proteins, and (**f**) polysaccharides in the soluble microbial products (SMPs) of the supernatant between the AMBR and VMBR. Comparison of the contents of (**g**) humic acids, (**h**) proteins, and (**i**) polysaccharides in the extracellular polymeric substances (EPSs) extracted from the sludge between the AMBR and VMBR. The terms TB-EPSs and LB-EPSs refer to tightly bound EPSs and loosely bound EPSs, respectively. Stages M1, M2, and M3 correspond to sampling times of 30, 60, and 90 days of the first operation cycle, respectively. M1-A/M1-V represent AMBR/VMBR at stage M1; M2-A/M2-V and M3-A/M3-V are defined analogously. The error bars represent the means ± SDs (*n* = 3).

Particle size distribution (PSD) analysis revealed clear differences in floc development among stages M1–M3 (Fig. 3a–c and Supplementary Fig. 15). At M1, the AMBR had a greater proportion of fine particles (<50 μm), indicating early generation due to aeration shear (Fig. 3a). In contrast, the VMBR demonstrated significantly larger average (*d*_ave_) and median (*d*_50_) particle sizes, exceeding those of the AMBR by 27.0% and 28.9%, respectively (Supplementary Fig. 15d). At M2, the AMBR maintained a higher fraction of sub-50 μm particles (Fig. 3b) and a lower *d*_10_ value (Supplementary Fig. 15b), reflecting a persistent fine particle population amidst a dynamic of floc breakup and regeneration. Moreover, the *d*_90_ of particles in the VMBR further increased to 92.6 μm (Supplementary Fig. 15e), indicating an increase in larger, structurally stable flocs. At M3, both systems displayed a more concentrated distribution in the 20–50 μm range (Fig. 3c), but the AMBR continued to exhibit a greater proportion of sub-20 μm particles, demonstrating that sustained high-intensity aeration hindered stable floc growth and led to a fragmented PSD profile^30^. Under low-shear vibration, the VMBR promoted continuous floc aggregation and maturation, yielding larger and denser macrostructures^20^.

The analysis of soluble microbial products (SMPs) provides further insight into fouling propensity (Fig. 3d–f and Supplementary Fig. 16). SMP concentrations were consistently lower in the VMBR system. This was attributed to reduced mechanical shear from targeted vibration, which minimized cell lysis and the passive release of intracellular and EPS components, coupled with anoxic conditions that suppress the aerobic metabolic activity driving SMP formation. Conversely, the aerobic AMBR tank promoted microbial activity and endogenous respiration, increasing SMP generation. By limiting both the physical release and the biochemical production of SMPs, the VMBR effectively reduced the pool of bioavailable foulants.

The composition of extracellular polymeric substances (EPSs) also differed (Fig. 3g–i and Supplementary Fig. 17). Although the TOC content in EPSs increased over time in both systems, the content of loosely bound EPSs (LB-EPSs), which have a greater impact on fouling, was consistently lower in the VMBR^32^. This finding indicates that compared with vigorous aeration shear, vibration induces less floc disruption, reducing LB-EPS detachment into the supernatant. The dynamic changes in LB-EPS composition suggest distinct fouling mechanisms between the two systems. Compositional analysis revealed distinct fouling mechanisms. Initially (M1), the protein content of LB-EPSs in the AMBR was nearly double that in the VMBR (0.037 vs. 0.019 mg g^−1^ VSS), suggesting a greater potential for rapid initial gel layer formation^32^. In the later stages (M2 and M3), the polysaccharide and humic acid contents significantly increased in the AMBR. The increase in polysaccharides reflects a microbial response to shear stress, resulting in the secretion of hydrophilic polymers that form dense gel networks^33^. Increased humic acid content enhances floc hydrophobicity and adhesion potential^34^. Consequently, the dominant fouling mechanism in the AMBR shifted from initial protein-mediated adhesion to a complex synergy of polysaccharide gelation and humic acid adsorption, leading to more resistant fouling layers.

### CFD analysis of the antifouling mechanism

**Fig. 4:**
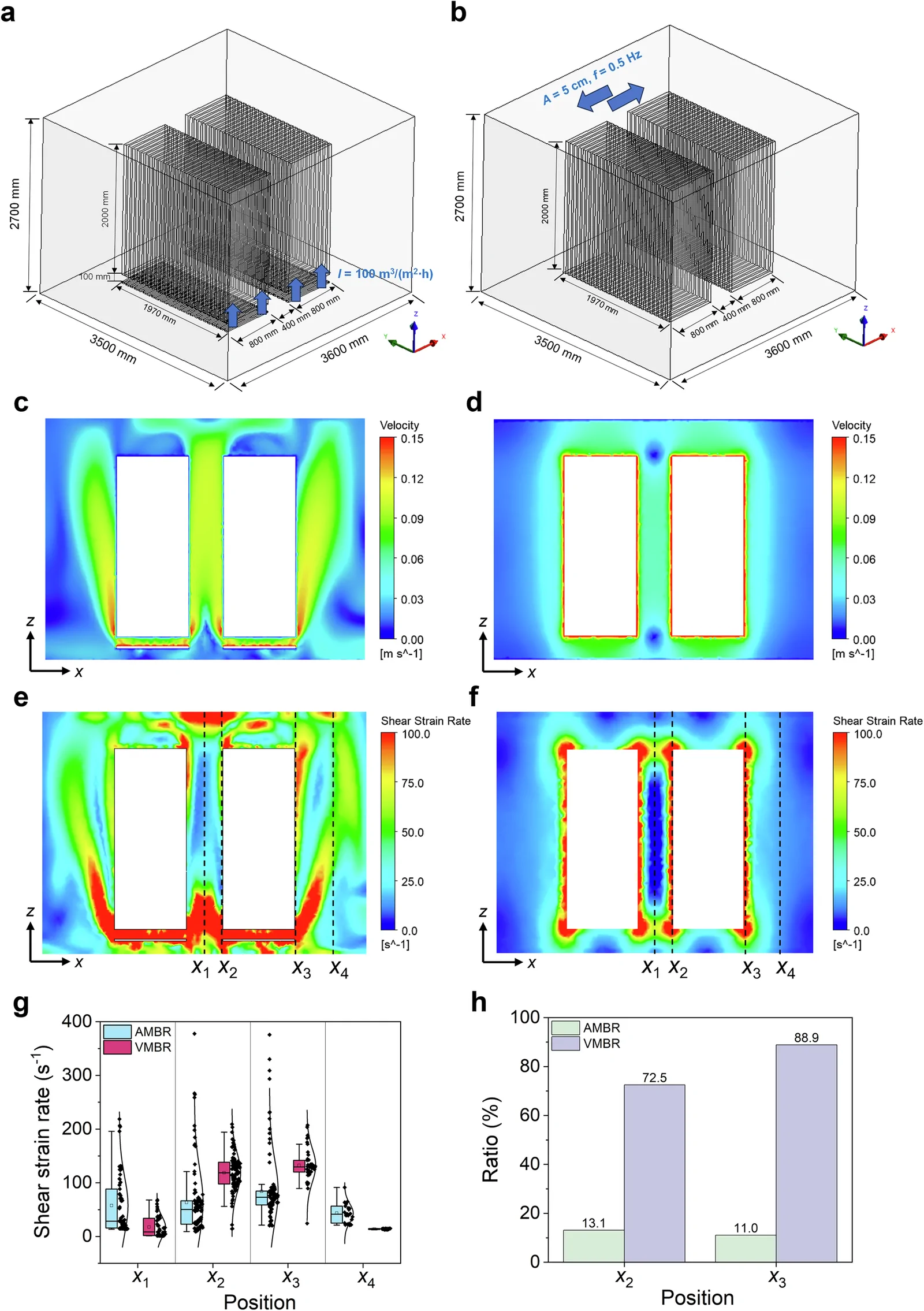
Comparison of flow field characteristics in the AMBR and VMBR membrane tanks. Physical models of the membrane tanks of the (**a**) AMBR and (**b**) VMBR. Each membrane tank has 2 stacks of membrane sheets spaced 30 mm apart. The AMBR applies bottom aeration with an intensity of 100 m^3^ m^−2^  h^−1^, while the VMBR employs horizontal reciprocating vibration with an amplitude (*A*) of 5 cm and a frequency (*f*) of 0.5 Hz. Velocity distribution of mixed liquor in the vertical cross section of the (**c**) AMBR and (**d**) VMBR. Fluid shear rate distribution in the vertical cross section of the (**e**) AMBR and (**f**) VMBR. **g** Statistical distribution of fluid shear rates on the *x*-axis for the AMBR and VMBR. Box plots show the median, quartiles, whiskers corresponding to 1.5× interquartile range and outlier points (*x*_1_–*x*_4_: AMBR/VMBR, *n* = 70/55, 107/91, 127/45, 27/16, respectively). **h** Comparison of the area percentage on the membrane surface where the fluid shear rate exceeds 100 s^−1^ between the AMBR and VMBR. Here, *x*_1_ = 0 (the mixed liquor region at the midpoint between the two stacks of membrane sheets), *x*_2_ = 0.2 m and *x*_3_ = 1 m (regions near the membrane surface), and *x*_4_ = 1.4 m (the mixed liquor region at the midpoint between the membrane stack and the outer boundary).

The AMBR and VMBR systems differ fundamentally in their approach to membrane fouling control. The former employs aeration, while the latter utilizes mechanical vibration, creating distinct hydrodynamic environments. To investigate the fouling mechanisms, CFD models were constructed to characterize the internal flow fields of both systems (Fig. 4a, b).

From a hydrodynamic perspective, the marked differences in fouling behavior stem from their divergent mechanisms for generating shear stress. In the AMBR system, shear production relies on aeration. Bubbles rising through the mixed liquor transfer momentum to the surrounding liquid via interphase forces, thereby inducing liquid circulation^35^. This indirect energy-transfer path from gas to liquid to the membrane surface involves significant efficiency losses. The CFD simulation results indicate that the bubble-induced flow field is strongly heterogeneous (Fig. 4c), with high-velocity zones forming near aeration inlets and membrane module gaps, whereas the flow velocity decreases in central regions, and distinct dead zones emerge at the top. Consequently, the shear rate distribution is nonuniform, ranging widely from less than 100 s^−1^ to more than 400 s^−1^ across the module (Fig. 4e). As bubbles increase and coalesce, their kinetic energy decreases, creating a region of weak shear at the upper section of the module.

Corroborating experimental evidence and postoperative imaging directly link these hydrodynamic traits to AMBR fouling characteristics. Postoperative membrane inspection revealed uneven fouling in the AMBR, with severe sludge cake accumulation at the top (Supplementary Fig. 18). Hydrodynamically, this pattern arises from the decay of shear intensity along the bubble rise path, which is sufficient near the bottom to suppress deposition but inadequate near the top to clear accumulated pollutants (Supplementary Fig. 19a). Additionally, CFD simulations suggest that the high turbulent kinetic energy in the AMBR, particularly in aerated zones, may promote sludge floc breakup, releasing colloidal and soluble substances that exacerbate irreversible fouling^20^. This aligns with the experimental findings and explains the higher fouling propensity of the AMBR.

In contrast, VMBR applies shear directly at the membrane surface via horizontal reciprocating vibration. The horizontal reciprocating motion of membrane modules generates periodic shear forces through direct solid‒liquid relative motion at the membrane‒liquid interface. The results of the CFD simulations confirm that this approach yields a more uniform flow and shear field, with elevated velocity and shear regions closely confined to the membrane surface (Fig. 4d, f). In addition, the wall shear distribution also shows consistent characteristics from bottom to top (Supplementary Fig. 19b). Analysis of the lateral shear rate profiles 1 mm from the membrane surface (Supplementary Fig. 20) reveals that the maximum and average shear rates are 170 s^−1^ and 115 s^−1^, respectively, for the VMBR. Although these values are lower than those in the AMBR (with maximum and average values of 505 s^−1^ and 188 s^−1^, respectively), the energy utilization of the VMBR is more efficient, as nearly all the input energy directly contributes to membrane surface fouling control.

Further CFD analysis of localized shear rate distributions provides deeper insight into the efficiency of each fouling control strategy (Fig. 4g–h). By comparing profiles at defined positions, from the bulk mixed liquor midpoint (*x*_1_, *x*_4_) to regions adjacent to the membrane surface (*x*_2_, *x*_3_), a critical contrast emerges. While the shear rates in the bulk liquid of the VMBR are approximately 70% lower than those in the AMBR, reflecting minimal energy dissipation, the shear near the membrane surface is 57–88% greater in the VMBR (Fig. 4g). More decisively, the area fraction where the shear rate exceeds 100 s^-1^ near the membrane surface is 73–89% for the VMBR, compared with only 11–13% for the AMBR (Fig. 4h). These results quantitatively demonstrate that mechanical vibration directs energy far more effectively toward the membrane‒liquid interface, creating a sustained and uniformly distributed scouring action precisely where it is needed for fouling control, whereas aeration dissipates most of its energy in bulk mixing, resulting in weak and uneven shear at the membrane surface.

In short, the fouling control of AMBR is constrained by inefficient energy transfer, heterogeneous shear distribution, and potential sludge fragmentation. The VMBR, through direct and uniform interfacial shear, offers a more effective and reliable fouling control strategy. Previous studies have largely attributed the reduced fouling rate in the VMBR to the decreased release of EPSs/SMPs^16,20^, but we provide a more fundamental mechanistic explanation for this phenomenon from the perspectives of uniform hydrodynamic shear distribution and the preservation of sludge floc integrity. These results demonstrate that the improved antifouling performance of the VMBR is directly driven by the targeted, uniform shear generated by mechanical vibration, an inherent hydrodynamic feature of the technology that is independent of the system’s DO concentration and recycle strategy.

### Energy consumption and carbon emissions

**Fig. 5:**
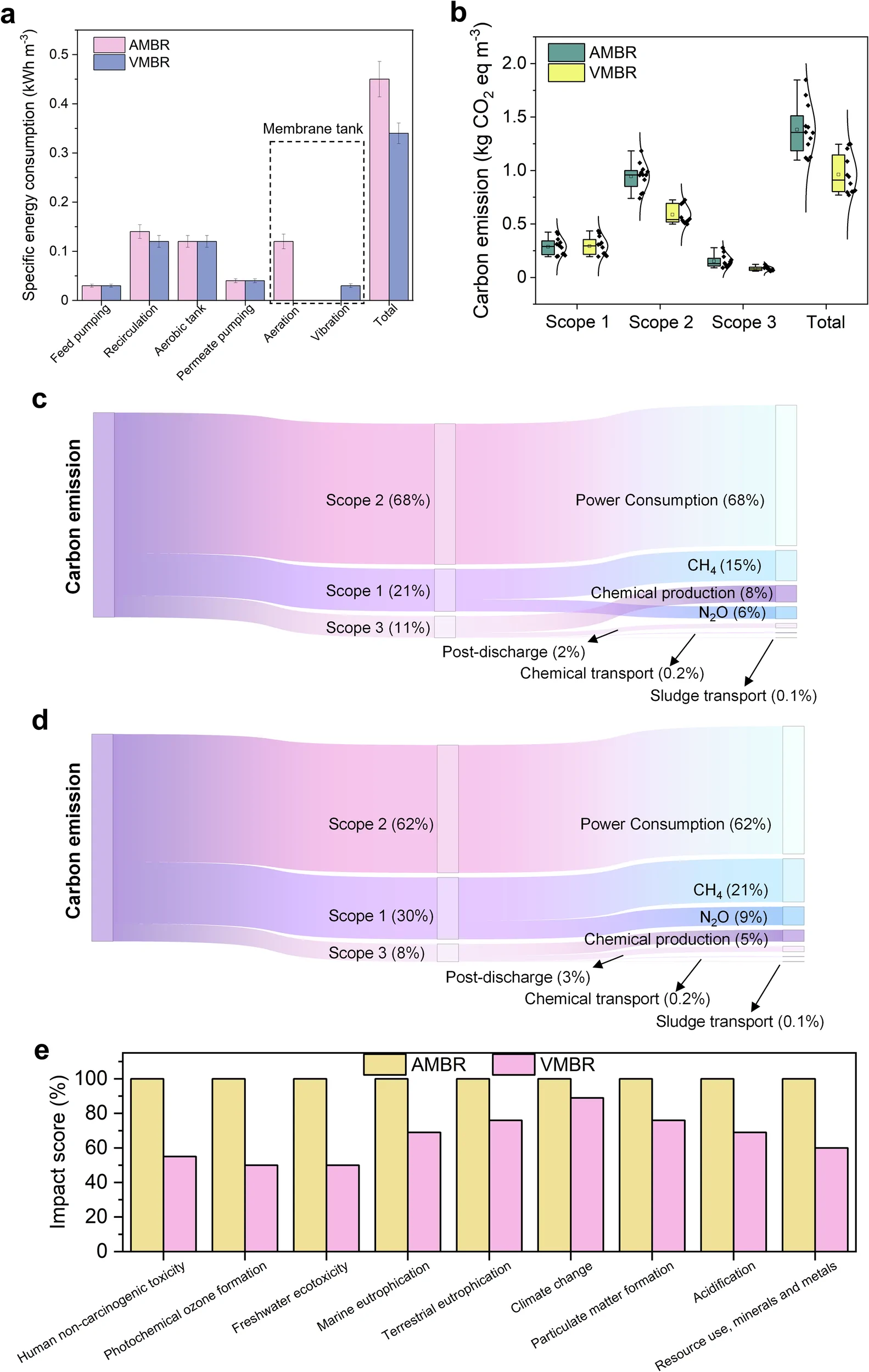
Energy consumption, carbon emission, and environmental impact assessment of the AMBR and VMBR systems. **a** The average specific energy consumption of each unit of the AMBR and VMBR systems. Energy consumption for membrane fouling control in membrane tanks (the dashed box)  refers to aeration in the AMBR and vibration in the VMBR. The error bars represent the means ± SDs (*n* = 44). **b** A comparative analysis of the carbon emissions from the AMBR and VMBR, categorized in accordance with the IPCC Guidelines into Scope 1 (direct emissions), Scope 2 (indirect emissions from energy consumption), Scope 3 (other indirect emissions), and total emissions. Box plots show the median, quartiles, whiskers corresponding to 1.5× interquartile range and outlier points (*n* = 12). The composition and sources of carbon emission for (**c**) AMBR and (**d**) VMBR. e Comparison of environmental impacts per ton of municipal wastewater treated by AMBR and VMBR systems, across 9 indicator categories in terms of human health (human noncarcinogenic toxicity and photochemical ozone formation), ecosystem impact (freshwater ecotoxicity, marine eutrophication, and terrestrial eutrophication), climate change, pollutant emission (particulate matter formation and acidification) and resource consumption (resource use, minerals, and metals).

Continuous monitoring of individual motor units provided comparative energy data for both systems (Fig. 5a). The total specific energy consumption was 0.45 ± 0.04 kWh m^−3^ for the AMBR and 0.34 ± 0.02 kWh m^−3^ for the VMBR, representing a 24% energy saving for the vibrating system. A detailed breakdown shows nearly identical energy use for pumping and biological treatment in both configurations. The crucial difference lies in the method of membrane fouling control. The AMBR relies on aeration, which consumes 0.12 kWh m^−3^, whereas the VMBR uses mechanical vibration, which requires only 0.03 kWh m^−3^. This 75% reduction stems from the direct “solid–liquid” energy transfer in the VMBR, which maximizes the conversion of electrical input into effective shear at the membrane surface. In contrast, the indirect “gas‒liquid‒solid” pathway in the AMBR leads to significant energy dissipation during bulk mixing and turbulence, as confirmed by CFD simulations.

Carbon emissions were calculated following the emission factor method of the Intergovernmental Panel on Climate Change (IPCC) across three scopes (Fig. 5b)^36^. While direct emissions (Scope 1) were similar for both systems at 0.29 kg CO_2_ eq m^−3^, the VMBR achieved significantly lower indirect emissions. Its Scope 2 emissions (from purchased electricity) were 0.59 kg CO_2_ eq m^−3^, 37% lower than the AMBR’s 0.94 kg CO_2_ eq m^−3^. Scope 3 emissions (from chemical production, transport, etc.) were 0.08 kg CO_2_ eq m^−3^ for the VMBR, which was 47% lower than the 0.15 kg CO_2_ eq m^−3^ for the AMBR. Consequently, the total carbon footprint of the VMBR (0.96 kg CO_2_ eq m^−3^) was 30% lower than that of the AMBR (1.38 kg CO_2_ eq m^−3^). The composition and sources of carbon emissions in the AMBR and VMBR are further illustrated in Fig. 5c, d. Although the enhanced denitrification in the VMBR system led to a marginal increase in direct N_2_O emissions, this was offset by significant reductions in indirect emissions from lower electricity consumption and reduced chemical use, particularly for phosphorus removal.

A comparative life cycle assessment (LCA) further substantiates the favorable environmental profile of the VMBR (Supplementary Table 1). With the AMBR system as the baseline (100%), the VMBR demonstrates substantial impact reductions across all nine evaluated categories (Fig. 5e), most notably in terms of photochemical ozone formation and freshwater ecotoxicity (both reduced by 50%) and human noncarcinogenic toxicity (reduced by 45%). These pronounced improvements are directly attributable to the operational advantages of the VMBR: its significantly lower energy consumption (reducing the burden from electricity generation) and the decrease in chemical usage for phosphorus removal (diminishing upstream production and transport impacts). The technology also achieves reductions in climate change, eutrophication, acidification, and particulate matter formation potential, stemming from its enhanced nutrient removal and the associated decrease in process-related emissions. Furthermore, a 40% reduction in mineral and metal resource use underscores the improved resource efficiency linked to lower chemical demand. Uncertainty analysis shows that the VMBR exhibited lower overall sensitivity than the AMBR, indicating more stable environmental performance against operational fluctuations (Supplementary Fig. 21). This comprehensive LCA confirms that the energy-efficient, low-chemical operational paradigm of the VMBR translates into broad and systemic environmental cobenefits, solidifying its role as a key technology for sustainable wastewater treatment.

## Discussion

Confirming and extending earlier findings from lab- and pilot-scale studies, this long-term, full-scale comparative study demonstrates that the VMBR project achieves improved performances to the conventional AMBR across critical metrics of sustainability and efficiency. The VMBR configuration achieves enhanced nutrient removal, with better-controlled membrane fouling and more robust operational stability. These advantages stem from its unique anoxic membrane tank environment and direct mechanical scouring mechanism, which together contribute to significant energy savings and reduced environmental footprints.

The transition from aeration-dependent to mechanical fouling control offers a more sustainable pathway for MBR design. By reducing the negative effects of bubble-induced shear, the VMBR system lessens floc disintegration and creates more favorable conditions for biological treatment. This strategy helps relieve the high energy demand that has restricted broader use of MBRs.

While this full-scale parallel study demonstrates the promising performance of VMBR, broader translational conclusions still require further multi-scenario validation. The full-life-cycle mechanical reliability, maintenance burden, retrofit feasibility for existing MBR facilities, and structural durability of large-scale vibration systems have yet to be comprehensively evaluated over the full membrane design service life (typically 5-8 years). Additionally, carbon emission accounting in this study relies on standardized IPCC emission factors, and future on-site continuous monitoring of greenhouse gas emissions would further improve the accuracy of carbon accounting. Moreover, the operational robustness of VMBR under extreme wastewater quality fluctuations, shock influent loading events, and long-term variable climatic conditions also warrants further investigation. These follow-up studies would help facilitate the broader engineering application of VMBR as a low-carbon, energy-efficient wastewater treatment technology.

## Methods

### Operational management of the AMBR and VMBR systems

The Beijing Doudian Wastewater Reclamation Plant, located in Doudian Town, Fangshan District, Beijing, is a full-scale municipal wastewater treatment plant that uses VMBR technology. The plant has a total design capacity of 15,000 m^3^ d^−1^, with equal capacities of 7500 m^3^ d^−1^ allocated to the AMBR and VMBR processes. It was commenced in May 2021, and after a temporary suspension from July to December 2023 due to flood damage, resumed stable continuous operation in December 2023 and has been running stably ever since. The plant contains four membrane tank galleries, with two equipped for the AMBR process and the other two for the VMBR process. Each gallery is installed with five membrane stacks, with a single stack area of 1750 m^2^ (35 × 50 m^2^ specification), yielding a total membrane area of 8750 m^2^ per gallery.

The recycling strategy for each system was designed following full-scale engineering optimal principles, dictated by the inherent DO characteristics of the membrane tank from each fouling control method. For the AMBR, the high-DO environment in the aerated membrane tank required a multi-step recycle pathway (membrane tank → aerobic tank → anaerobic/anoxic tanks) to avoid disrupting the anaerobic environment, with a total recycle ratio of 500% to meet sludge retention and nitrogen removal requirements. For the VMBR, the vibration-enabled anoxic membrane tank (DO < 0.1 mg L^−1^) allowed direct recirculation of mixed liquor to the anaerobic tank without oxygen interference, enabling a reduced recycle ratio of 200% while maintaining stable process performance. All other key operational parameters of the biological treatment train, including hydraulic retention time (HRT) and aeration intensity of the aerobic tank, were identical between the two parallel systems to ensure a controlled and rigorous comparison. This head-to-head comparison against the industry-standard conventional AMBR aligns with established MBR research and engineering consensus, as a system without any fouling control measures cannot sustain stable filtration and thus cannot serve as a valid experimental control.

The VMBR system was actuated by a horizontal reciprocating motion, configured to operate at a frequency of 0.5 Hz and an amplitude of 5 cm (Supplementary Movie 1). These parameters were selected through full-scale pre-commissioning to balance fouling control efficiency and long-term mechanical stability. The AMBR system operated with an aeration intensity of 100 ± 5 m^3^ m^−2^ h^−1^ (Supplementary Movie 2), which represented the conventional full-scale design value at the site to ensure effective membrane scouring and stable fouling control, allowing a fair comparison between the two systems. The design membrane flux for both the AMBR and VMBR systems is 18 LMH. Online cleaning is performed on average once per week using a sodium hypochlorite (NaClO) solution with a concentration of 800–1,500 ppm for 90–120 min. Offline cleaning is conducted approximately every six months. This process involves first rinsing the membrane surface sludge with a high-pressure water jet after the modules are lifted out. The modules are then soaked in a 4000–5000 ppm NaClO solution for 12 h, followed by an 8-h soak in a 20000 ppm citric acid solution, achieving a flux recovery rate exceeding 95%.

To systematically evaluate the treatment efficacy and operational stability, a comprehensive monitoring protocol was implemented. The TMP was logged continuously by the supervisory control and data acquisition system, providing real-time data on fouling propensity as a key metric of system stability. The DO concentration, pH, and temperature were monitored online with a multimeter (WTW, pH/Oxi340i). For water quality assessment, the samples were collected daily from the raw influent and the effluents of both membrane systems, and analyzed for key parameters, including COD, TP, TN, and NH_4_^+^-N, using standard analytical methods. The energy consumption of both the membrane tank motors and the entire process was monitored in real time via power meters installed on each functional unit.

### Characterization of membrane fouling layers

The membrane filament samples were collected from the periphery of the modules in both the AMBR and VMBR systems. Upon collection, the samples were immediately placed in sealed bags containing cotton balls soaked in 0.9% NaCl solution and transported to the laboratory for subsequent foulant characterization.

The micromorphology of the fouling layer on the membrane surface was examined using a scanning electron microscope (SEM, JSM-7001F, JEOL, Japan) at an accelerating voltage of 15.00 kV. Before SEM imaging, the samples were freeze-dried for 24 h and sputter-coated with a palladium layer to ensure conductivity. Energy dispersive X-ray spectroscopy (EDX, Oxford Instruments, UK) was coupled with SEM to perform qualitative and semiquantitative analyses of the elements in the foulant layer, identifying the primary inorganic constituents and their relative abundances.

For analysis of organic foulants, the membrane filaments were cut into pieces and immersed in a 0.5% NaClO solution, followed by shaking at 150 rpm for 2 h to elute the contaminants. The eluate was filtered through a 0.45 μm filter and analyzed for concentrations of total organic carbon (TOC, using a TOC-L CPH analyzer, Shimadzu, Japan, Supplementary Method 1), polysaccharides (via the phenol‒sulfuric acid method^37^, Supplementary Method 2), proteins (using a BCA assay kit, Beyotime, China, Supplementary Method 3), and humic substances (by the modified Lowry method^38^, Supplementary Method 4). The characteristics of the dissolved organic matter in the eluate were further analyzed using three-dimensional excitation‒emission matrix (EEM) fluorescence spectroscopy (F-7000, Hitachi, Japan). The fluorescence spectra were recorded with excitation wavelengths from 200 to 400 nm (5-nm interval) and emission wavelengths from 250 to 500 nm (5-nm interval) at a scan speed of 12,000 nm min^−1^ and a PMT voltage of 600 V. The resulting EEMs were processed in MATLAB to remove Raman and Rayleigh scattering.

For biomass quantification, membrane filaments were cut and placed in a 15 mL centrifuge tube with 3 mL of 1× PBS solution. The tube was vortexed for 1 min, and this washing step was repeated twice. The combined eluate was used to determine the adenosine triphosphate (ATP) content as an indicator of viable biomass. The ATP concentration was measured using a BacTiter-Glo™ ATP kit (Promega (Beijing) Biotech Co., Ltd.) on the basis of the luciferin–luciferase reaction^39^, and the results were normalized to the membrane surface area.

### Determination of physicochemical properties of the mixed liquor in the membrane tank

The mixed liquor samples were collected from the membrane tanks of both the AMBR and VMBR systems. On each sampling occasion, 1 L of mixed liquor was collected in sterile bags and immediately transported to the laboratory for processing and analysis.

To obtain the supernatant for SMP analysis, 50 mL of the mixed liquor was centrifuged at 1649 × *g* for 5 min at 4 °C (Allegra X-30R centrifuge, Beckman, USA). The resulting supernatant was then filtered through 0.7 μm glass fiber filter paper (Whatman, UK). To inhibit microbial growth, sodium azide (NaN_3_) was added to the filtered supernatant to a final concentration of approximately 3 mmol L^−1^, and the samples were stored at 4 °C until analysis.

The extraction of EPS was performed sequentially following SMP separation. After the initial centrifugation step, the pellet was resuspended to the original 50 mL volume using a prewarmed (50 °C) 0.05% NaCl solution, followed by vigorous vortex mixing for 1 min. The suspension was then centrifuged again under the same conditions (1649 × *g*, 5 min, 4 °C). The supernatant from this step was filtered through 0.7 μm glass fiber filter paper (Whatman, UK), yielding the LB-EPS fraction. The remaining pellet was subsequently resuspended in 50 mL of 0.05% NaCl solution and incubated in a water bath at 60 °C for 30 min. This was followed by a final centrifugation and filtration step, with the resulting filtrate designated the TB-EPS fraction^40^.

The concentrations of TOC in the SMP and EPS fractions were determined using a TOC analyzer (TOC-V CPH, Shimadzu, Japan) to quantify the overall organic content. Furthermore, the concentrations of specific organic components, namely, polysaccharides, proteins, and humic substances, were measured using the analytical methods described above.

Mixed liquor samples for microbial community analysis were collected in triplicate from the membrane tanks of both the AMBR and VMBR systems, with consistent sampling volume and location to ensure comparability. After collection, the samples were placed on ice, immediately transported to the laboratory, and centrifuged to harvest biomass pellets, which were aliquoted into sterile centrifuge tubes and stored at −80 °C until analysis. Total genomic DNA was extracted from the pellets using a commercial kit, with DNA concentration and integrity verified via spectrophotometry and 1% agarose gel electrophoresis. The V4 hypervariable region of the bacterial 16S rRNA gene was amplified using the barcoded universal primer pair 515F/806R, with purified PCR amplicons pooled into equimolar libraries and sequenced on the Illumina NovaSeq 6000 platform. Raw sequencing data were processed using QIIME 2 (v2023.5), including quality filtering, denoising to generate amplicon sequence variants, and taxonomic classification against the SILVA 138.1 database, with statistically significant differences in genus-level relative abundance between the two systems assessed via two-tailed Student’s *t* test (significance threshold *p* < 0.05).

### CFD simulation

In this study, CFX (version 24.1; ANSYS Inc., USA) was used to conduct CFD simulations of the AMBR and VMBR systems, with the goal of investigating the differences in their internal flow field characteristics. For simplicity and computational efficiency, the curtain-type membrane modules were represented as flat-sheet membranes. The setup comprised two columns, with each column containing 25 flat sheets. In the AMBR configuration, one aeration branch pipe was positioned at the bottom of each membrane sheet. To reduce computational cost, the perforated aerators at the bottom were modeled as rectangular aeration boxes where the top surface served as the air inlet with the superficial gas velocity applied^12^. The detailed geometric models and relevant parameters for both systems are provided in Fig. 4.

The constructed geometries were subsequently discretized using a structured hexahedral meshing scheme to enhance the mesh quality, minimize potential deformation, and control the total cell count. Local refinement was applied near the walls and the aeration pipes at a growth rate of 1.2. The maximum mesh skewness was maintained below 0.8, indicating high mesh quality. A grid independence study was performed using average shear strain rate as the key indicator, with a relative difference of less than 10% between the optimized mesh (~1.84 million elements) and a finer mesh (Supplementary Fig. 22), which meets the CFD accuracy requirement while balancing computational cost.

The governing equations for both systems were the incompressible Navier‒Stokes equations. For the AMBR system, the Eulerian‒Eulerian multiphase model was adopted to simulate the gas‒liquid two-phase flow^41^. The mixed liquor was assumed to be perfectly mixed within the reactor and defined as the continuous phase, with a density of 1005 kg m^−3^ and a dynamic viscosity of 0.001 Pa·s, matching the measured value at the system’s initial stable operation stage (Supplementary Fig. 12). The gas phase was treated as the dispersed phase and modeled as an ideal gas with a dynamic viscosity of 1.855 × 10^−5^ Pa·s. Interphase momentum exchange included drag (using the Schiller–Naumann model), lift (using the Legendre–Magnaudet model), and turbulent dispersion forces (using the Lopez de Bertodano model) to accurately capture bubble dynamics, including rising, swinging, and interaction with the liquid. The shear stress transport (SST) k-ω turbulence model was selected because of its superior performance in handling separated flows and resolving near-wall regions. For the VMBR system, the fluid was treated as a single phase. The horizontal reciprocating vibration of the membrane module was simulated using the dynamic mesh technique with the elastic smoothing method. The membrane motion was controlled by a user-defined function (UDF), specifying a displacement following a harmonic function: *x*(*t*) = 0.05 sin(2π × 0.5 *t*) m, corresponding to an amplitude of 5 cm and a frequency of 0.5 Hz.

Boundary conditions were defined on the basis of physical realism. For the AMBR system, the aeration surface was set as a velocity inlet, corresponding to a specific aeration intensity of 100 m^3^ m^−2^ h^−1^. The top surface of the tank was defined as a degassing opening boundary with a relative pressure of *P* = 0 Pa. All the other walls were set as no-slip boundaries. In the VMBR system, the membrane surfaces were defined as moving walls whose motion was governed by the UDF, whereas all the other tank walls were stationary no-slip boundaries. Given that the membrane permeation velocity (~10^-6^ m s^−1^) was significantly lower than the characteristic velocity within the reactor (~0.1 m s^−1^), all the membrane surfaces were simplified as impermeable walls^42–44^.

For the solution settings, the pressure-based coupled transient solver was used for both cases. The SIMPLE algorithm was employed for pressure‒velocity coupling. A high-resolution advection scheme was selected for the discretization of the convective terms to ensure solution accuracy^45^. Solution convergence was monitored by tracking the residual curves; the calculation was considered converged when the residuals leveled off and fell below 10^−3^ ^46^. Following the computations, flow field analysis was performed in the postprocessing module.

### Calculation of carbon emissions and environmental impacts

The operational energy consumption for both the AMBR and VMBR processes was calculated on the basis of continuous, component-level measurements. The total specific energy consumption for each system was determined as the sum of the individually metered consumption of its core electrical units. The energy inputs common to both configurations included the feed pump, the mixers for sludge recirculation, the blowers supplying air to the aerobic biological zone, and the effluent extraction pumps. The key distinction lies in the membrane fouling control method. In the AMBR process, this was represented by the energy consumed for membrane tank aeration, whereas in the VMBR process, it was the energy used by the mechanical vibration system. Real-time power data from each unit was logged by the plant’s supervisory control and data acquisition (SCADA) system.

Carbon emissions were quantified following the emission factor approach outlined by the IPCC. Scope 1 emissions refer to direct greenhouse gas releases during treatment, which include CH_4_ from anaerobic conditions and sludge handling and N_2_O from nitrification and denitrification. Scope 2 emissions represent indirect carbon emissions resulting from the consumption of purchased electricity or heat, such as those associated with energy-intensive units, including wastewater lifting, aeration, and sludge treatment. Scope 3 emissions encompass other indirect emissions beyond Scope 2, covering the production of consumed chemicals and the transportation of various goods and materials. In this study, N_2_O from biological nitrogen removal and CH_4_ from anaerobic processes were estimated using influent–effluent nutrient balances and default emission factors (0.005 kg N_2_O-N/kg N removed for N_2_O; a maximum methane-producing capacity of 0.6 kg CH_4_/kg biochemical oxygen demand (BOD) with a methane correction factor of 0.165 for CH_4_) for both the AMBR and VMBR systems. The indirect emissions from purchased electricity were calculated by multiplying the metered energy consumption by a regional grid emission factor of 0.9419 kg CO_2_-e/kWh. The other indirect emissions included the production and transport of major chemicals, particularly ferric chloride (FeCl_3_), for phosphorus removal, for which an emission factor of 1.0–2.17 t CO_2_-e/t FeCl_3_ was applied, and the transport of dewatered sludge for final disposal. All calculations were based on operational data, with emission factors and global warming potentials sourced from authoritative guidelines, including the IPCC and region-specific technical references.

An LCA was conducted to compare the environmental impacts of the full-scale AMBR and VMBR systems. The functional unit was defined as treating 1 m^3^ of municipal wastewater to meet local discharge standards. The system boundary of LCA followed a framework focused on the operational phase of the systems (detailed in Supplementary Fig. 23), which encompassed: (1) direct operational inputs and emissions, including energy consumption for biological treatment, pumping, and membrane fouling control, as well as direct emissions of N_2_O and CH_4_; (2) relevant upstream and downstream processes, including the production and transport of chemicals (FeCl_3_) for phosphorus removal, upstream emissions from regional grid electricity generation (predominantly fossil fuel-based), and the transport and incineration of dewatered sludge. Environmental impacts related to membrane modules (including production, replacement, and end-of-life disposal) are explicitly excluded from the system boundary and accounting scope. Primary inventory data, including specific energy use and chemical dosage rates, were collected during the operational monitoring period. Background data for upstream processes were sourced from the Ecoinvent 3.8 database and adapted to reflect Chinese industrial and energy contexts. Life cycle impact assessment was performed using OpenLCA software and the Environmental Footprint (EF) 3.0 method at the midpoint level. The impacts were evaluated across nine categories, which were grouped into five areas: human health (noncarcinogenic toxicity and photochemical ozone formation), ecosystem quality (freshwater ecotoxicity, marine eutrophication, and terrestrial eutrophication), climate change, atmospheric pollution (particulate matter formation and acidification), and resource consumption (minerals and metals). To enable a direct comparison, the impact results for the AMBR system were normalized to 100% for each category, providing a baseline against which the relative reduction achieved by the VMBR system was calculated and expressed as a percentage. Uncertainty analysis was conducted via Monte Carlo simulation (1000 iterations) to assess the robustness of the LCA results.

### Statistical analysis

The statistical analyses were performed using GraphPad Prism 9 software. Two-tailed Student’s *t* tests were applied to assess the statistical significance of differences between the AMBR and VMBR groups for water quality indicators, sludge physicochemical properties, microbial relative abundances and other quantitative data. The significance was denoted as follows: *, *p* < 0.05; **, *p* < 0.01; ***, *p* < 0.001; ns, not significant.

No statistical method was used to predetermine sample size. No data were excluded from the analyses. The experiments were not randomized. The Investigators were not blinded to allocation during experiments and outcome assessment.

## Supplementary information

Supporting Information

Description of Additional Supplementary File

Supplementary Movie 1. Video of VMBR operation

Supplementary Movie 2. Video of AMBR operation

Transparent Peer Review file

## Source data

Source data

## Data Availability

All the relevant data that support the results of this study are presented in the main text and Supplementary Information. Source data are provided with this paper.
