# Supplementary material for: Vibrating membrane bioreactors enhance nutrient removal and energy efficiency in municipal wastewater treatment
Source: Nat Commun. 2026 Jul 20;17:8854. doi: 10.1038/s41467-026-75747-6 (PMC13500542; doi:10.1038/s41467-026-75747-6)
Supplement: Supplementary file 1 [file 41467_2026_75747_MOESM1_ESM.pdf]

Supporting Information

Vibrating membrane bioreactors enhance nutrient removal and energy efficiency in municipal wastewater treatment

Weichen Lin[1, 2], Tao Xue[3], Haojie Ding[2], Ziwei Liu[2], Caiyun Zhang[3], Kang Xiao[4], Chunsheng Chen[3,*], Xia Huang[2, 5*]

[1] School of Environment, Beijing Normal University, Beijing 100875, China

[2] State Key Laboratory of Regional Environment and Sustainability, School of Environment, Tsinghua University, Beijing 100084, China

[3] Beijing OriginWater Membrane Technology Co., Ltd., Beijing 101407, China

[4] College of Resources and Environment, University of Chinese Academy of Sciences, Beijing, 100049, China

[5] Research and Application Center for Membrane Technology, School of Environment, Tsinghua University, Beijing 100084, China

*Corresponding authors:

chenchunsheng@originwater.com (C. Chen); xhuang@tsinghua.edu.cn (X. Huang)

Table of Contents

Supplementary Fig. 1.. Page S3
Supplementary Fig. 2 .... Page S4
Supplementary Fig. 3 .... Page S5
Supplementary Fig. 4 .... Page S6
Supplementary Fig. 5 .... Page S7
Supplementary Fig. 6 .... Page S8
Supplementary Fig. 7 .... Page S9
Supplementary Fig. 8 .... Page S10
Supplementary Fig. 9 .... Page S11
Supplementary Fig. 10 .... Page S12
Supplementary Fig. 11 .... Page S13
Supplementary Fig. 12 .... Page S14
Supplementary Fig. 13 .... Page S15
Supplementary Fig. 14 .... Page S16
Supplementary Fig. 15 .... Page S17
Supplementary Fig. 16 .... Page S18
Supplementary Fig. 17 .... Page S19
Supplementary Fig. 18 .... Page S20
Supplementary Fig. 19 .... Page S21
Supplementary Fig. 20 .... Page S22
Supplementary Fig. 21 .... Page S23
Supplementary Fig. 22 .... Page S24
Supplementary Fig. 23 .... Page S25
Supplementary Table 1 .... Page S26
Supplementary Method 1 .... Page S27
Supplementary Method 2 .... Page S28
Supplementary Method 3 .... Page S29
Supplementary Method 4 .... Page S30

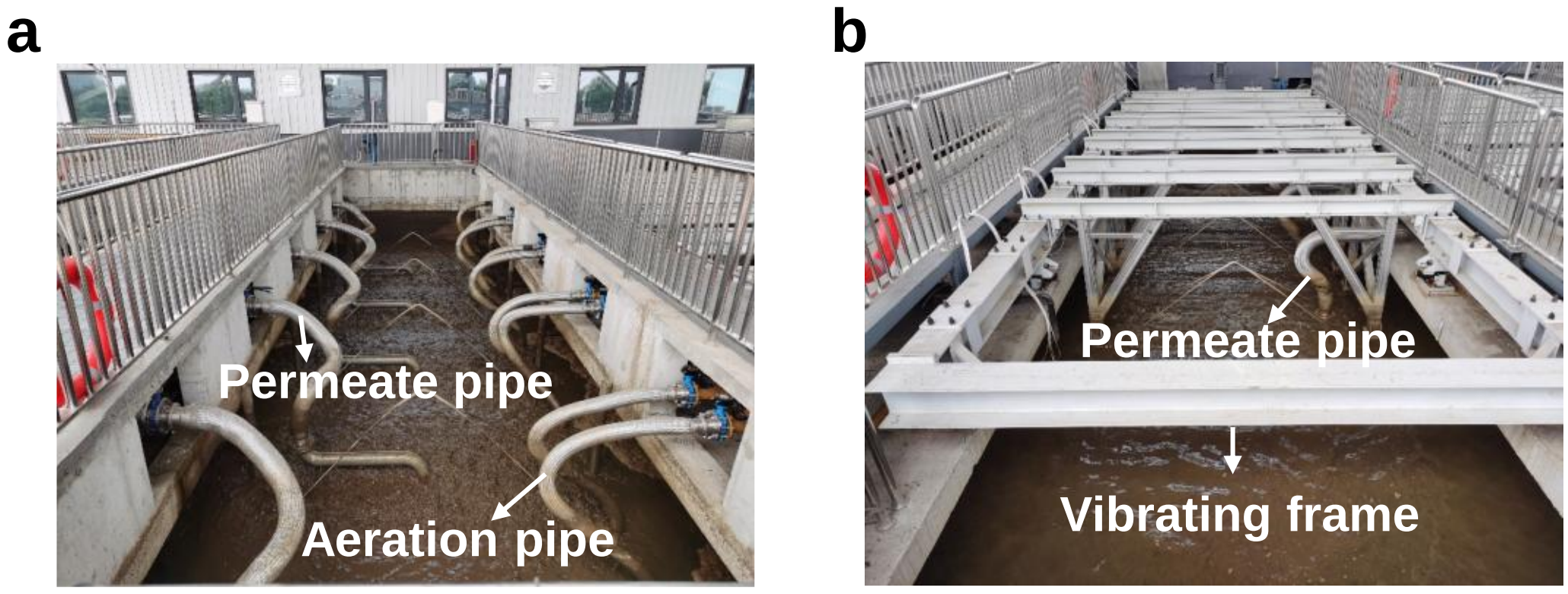

Supplementary Fig. 1 Field photos of the membrane tanks of the full-scale AMBR and VMBR systems. a, AMBR membrane tank with permeate pipes and bottom aeration pipes. b, VMBR membrane tank with permeate pipes and vibrating frame.

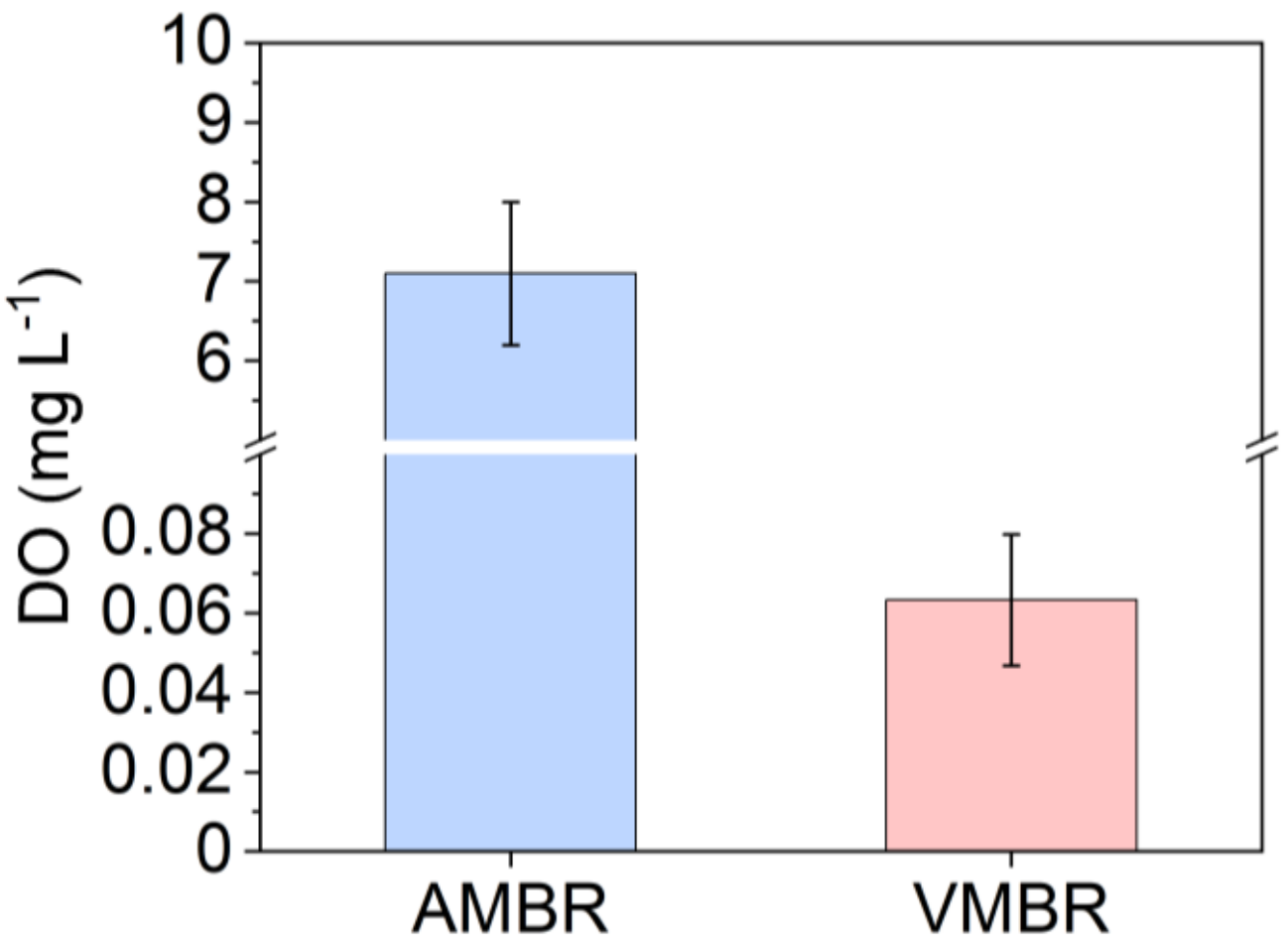

Supplementary Fig. 2 DO concentration in the membrane tanks of AMBR and VMBR. Error bars in the figures represent the SD (*n* = 21) and data are presented as mean values ± SD.

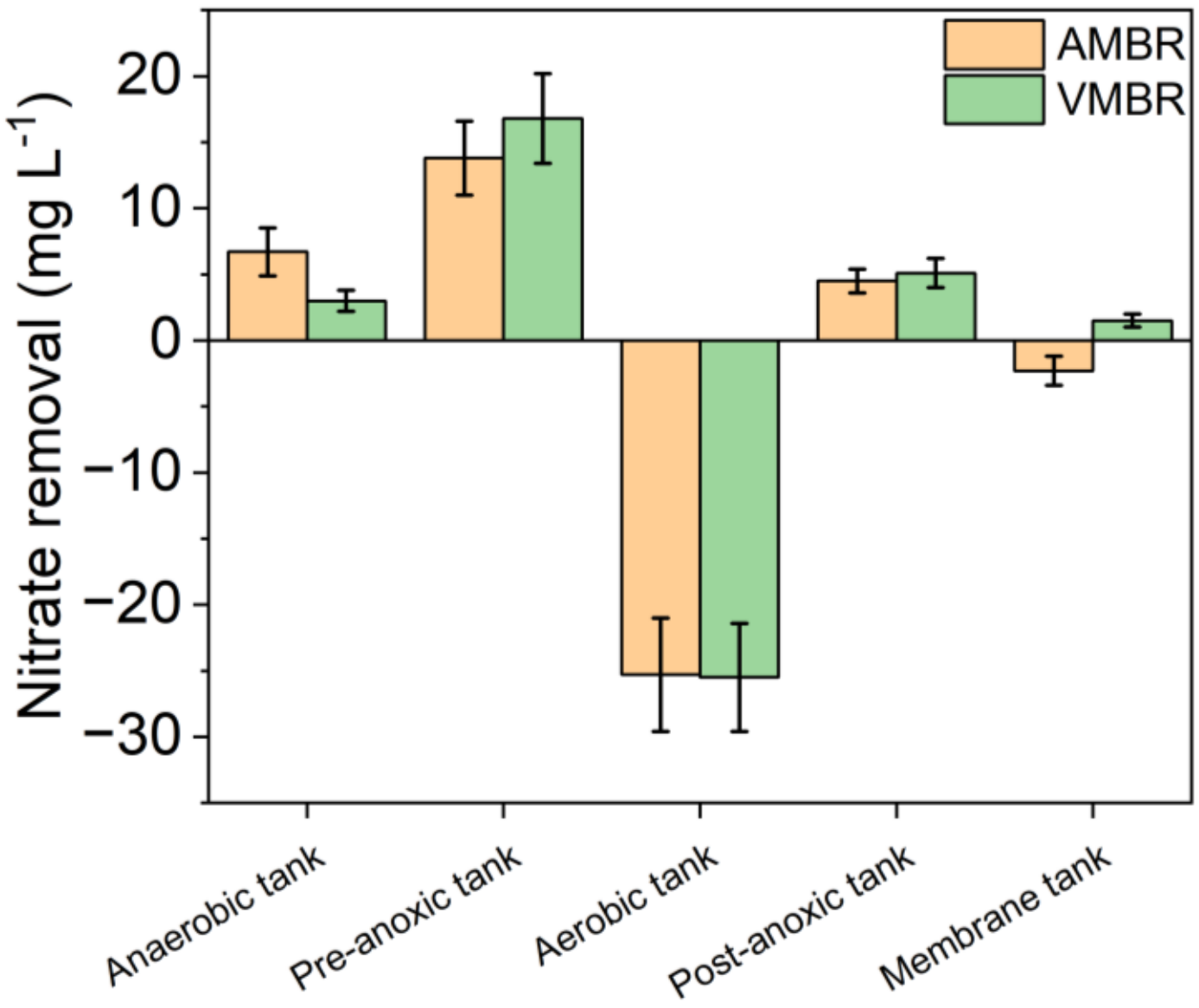

Supplementary Fig. 3 Comparison of nitrate nitrogen removal in individual units within AMBR and VMBR systems, with negative values indicating release into the unit. Error bars in the figures represent the SD (*n* = 12) and data are presented as mean values ± SD.

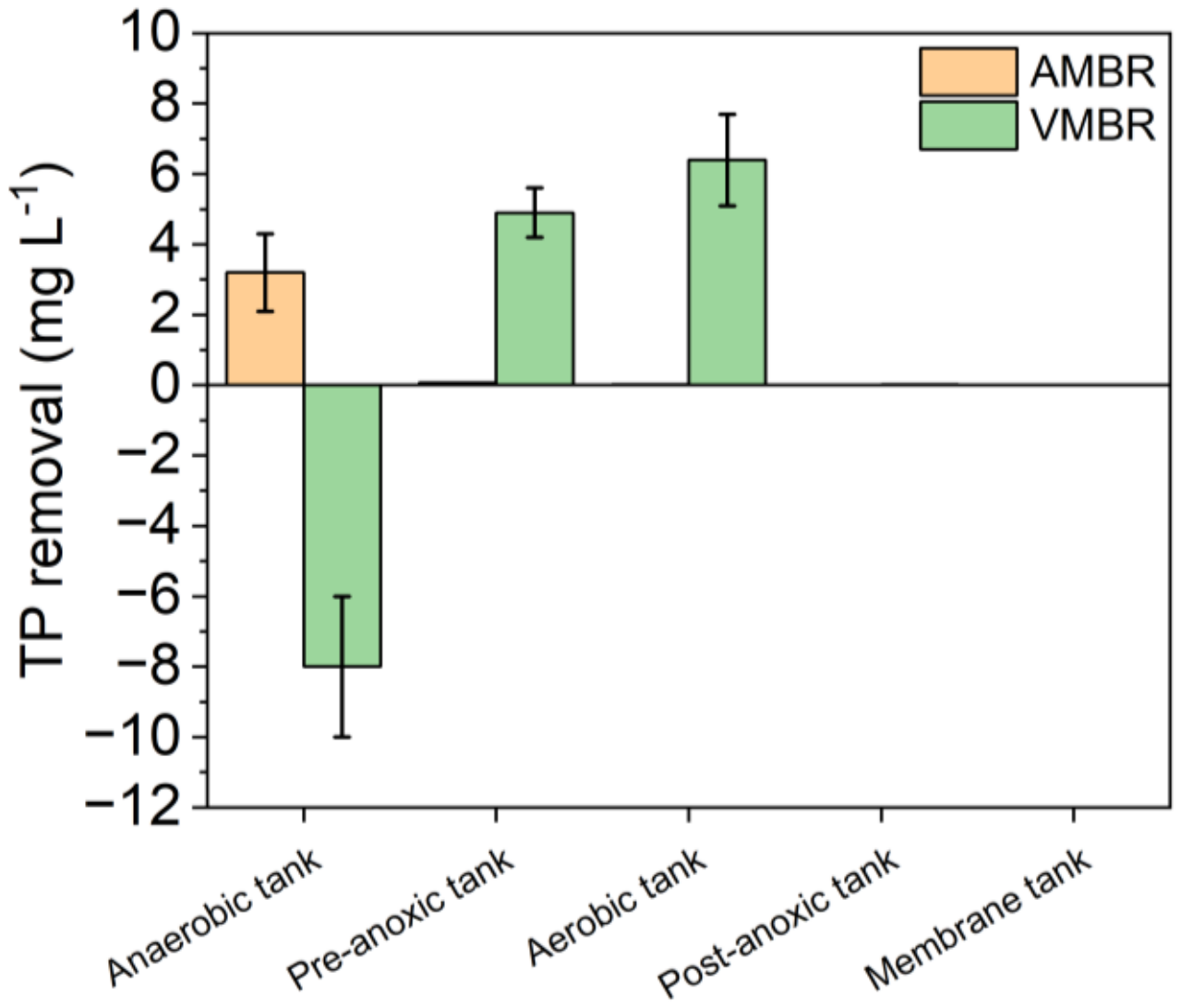

Supplementary Fig. 4 Comparison of TP removal in individual units within AMBR and VMBR systems, with negative values indicating release into the unit. Error bars in the figures represent the SD (n = 12) and data are presented as mean values ± SD.

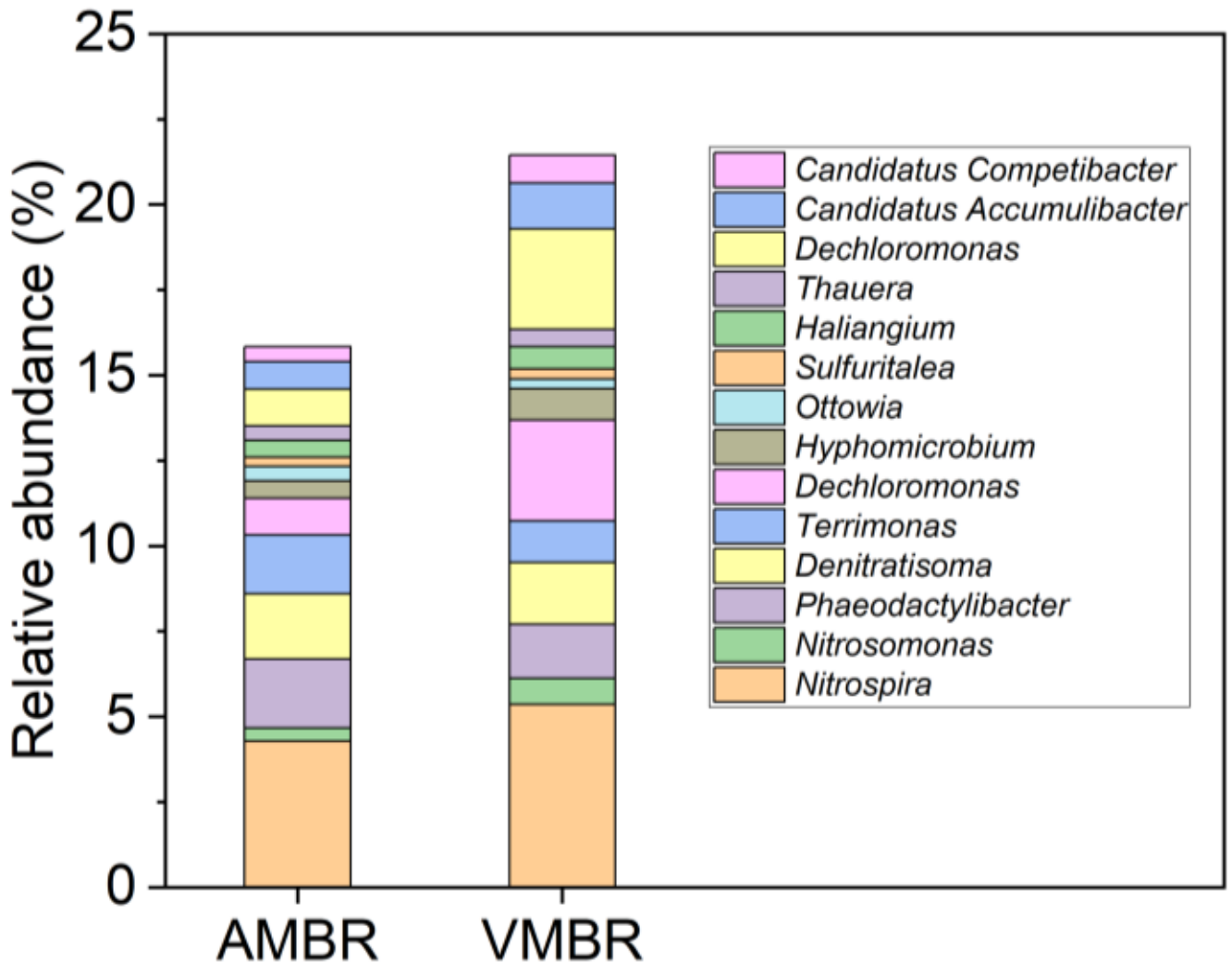

Supplementary Fig. 5 Comparison of the relative abundance of functional bacterial communities associated with nitrogen and phosphorus removal at the genus level in the mixed liquor of the membrane tank of AMBR and VMBR systems.

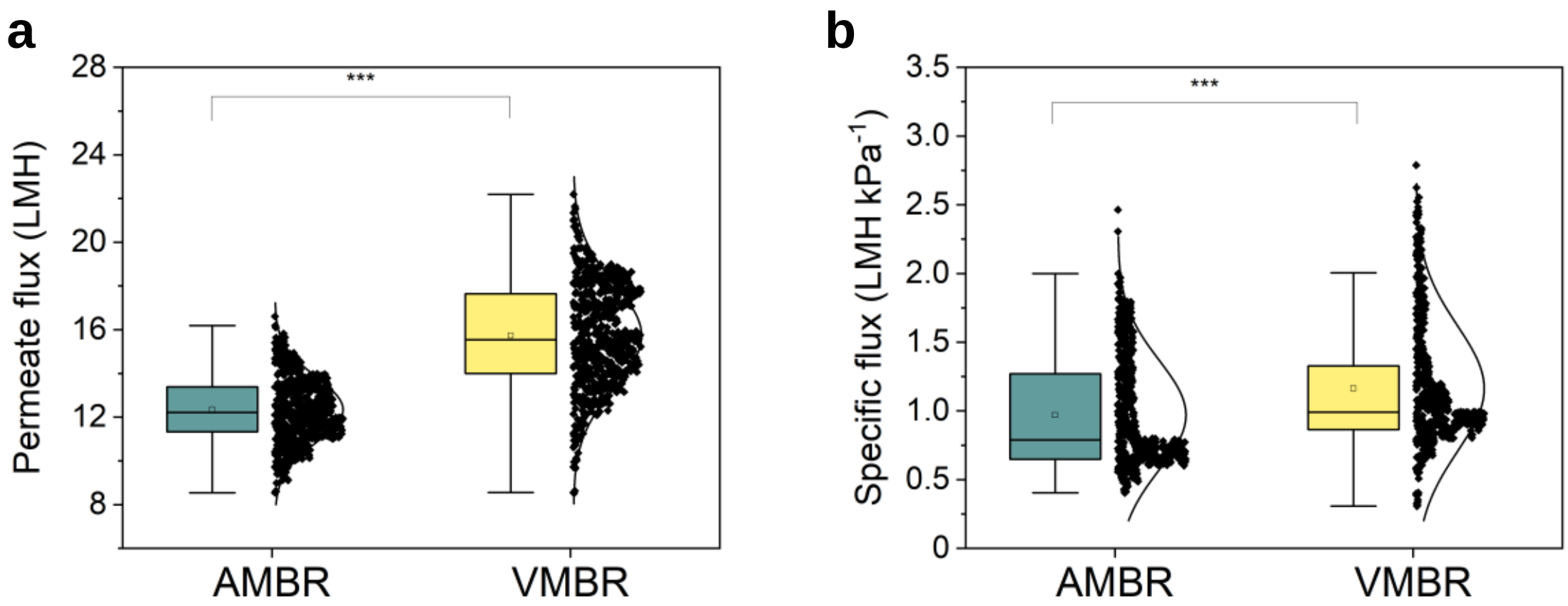

Supplementary Fig. 6 Box-plot comparison of the long-term performance between AMBR and VMBR processes, showing the distributions of (a) permeate flux and (b) specific flux over the operational period. Box plots show the median, quartiles, whiskers corresponding to 1.5× interquartile range and outlier points ($n \geq 663$). Statistical significance of differences in medians was assessed using the nonparametric Mann-Whitney U test (*, $p < 0.05$; **, $p < 0.01$; ***, $p < 0.001$; ns, not significant).

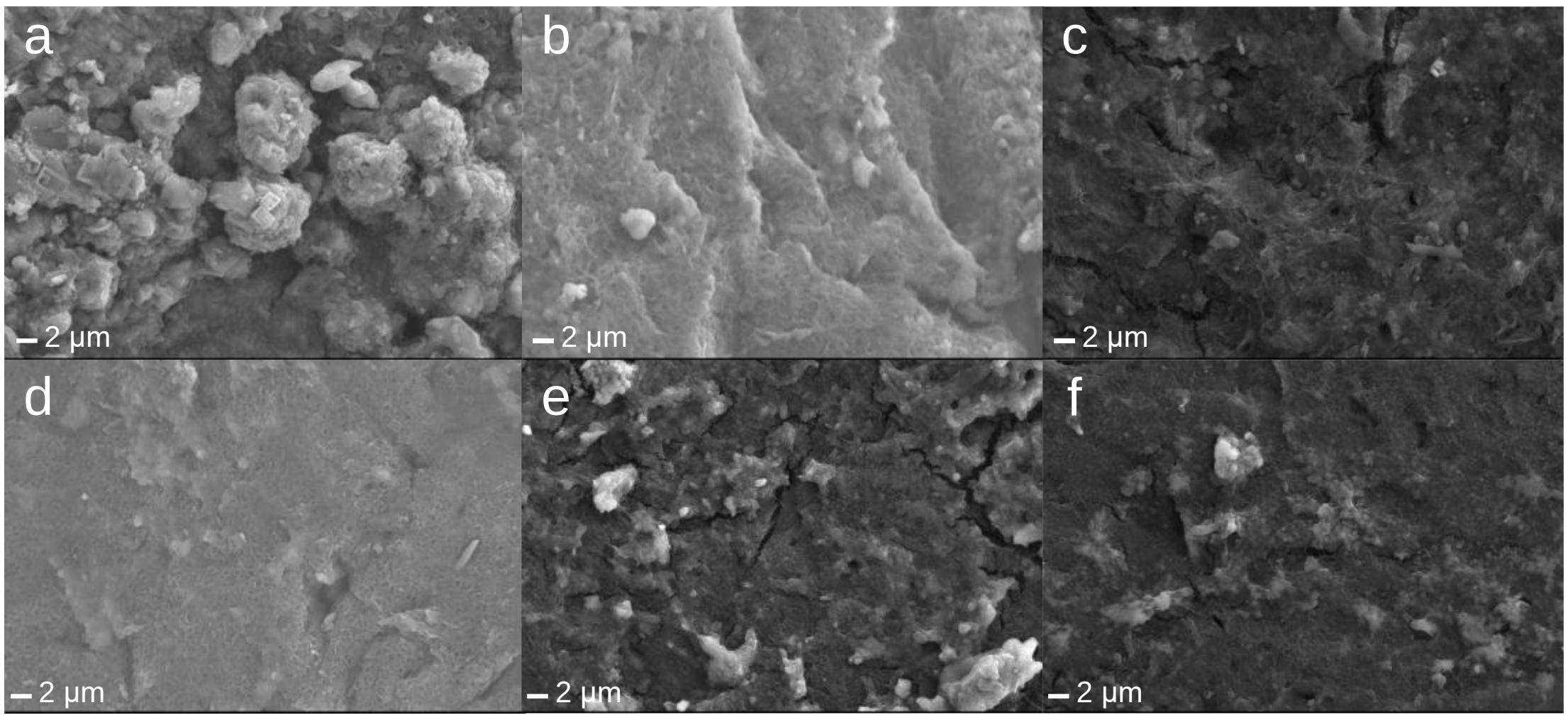

Supplementary Fig. 7 SEM images showing the fouling layer on the membrane surface in AMBR and VMBR at different stages (M1, M2 and M3): (a) M1-AMBR, (b) M2-AMBR, (c) M3-AMBR, (d) M1-VMBR, (e) M2-VMBR and (f) M3-VMBR.

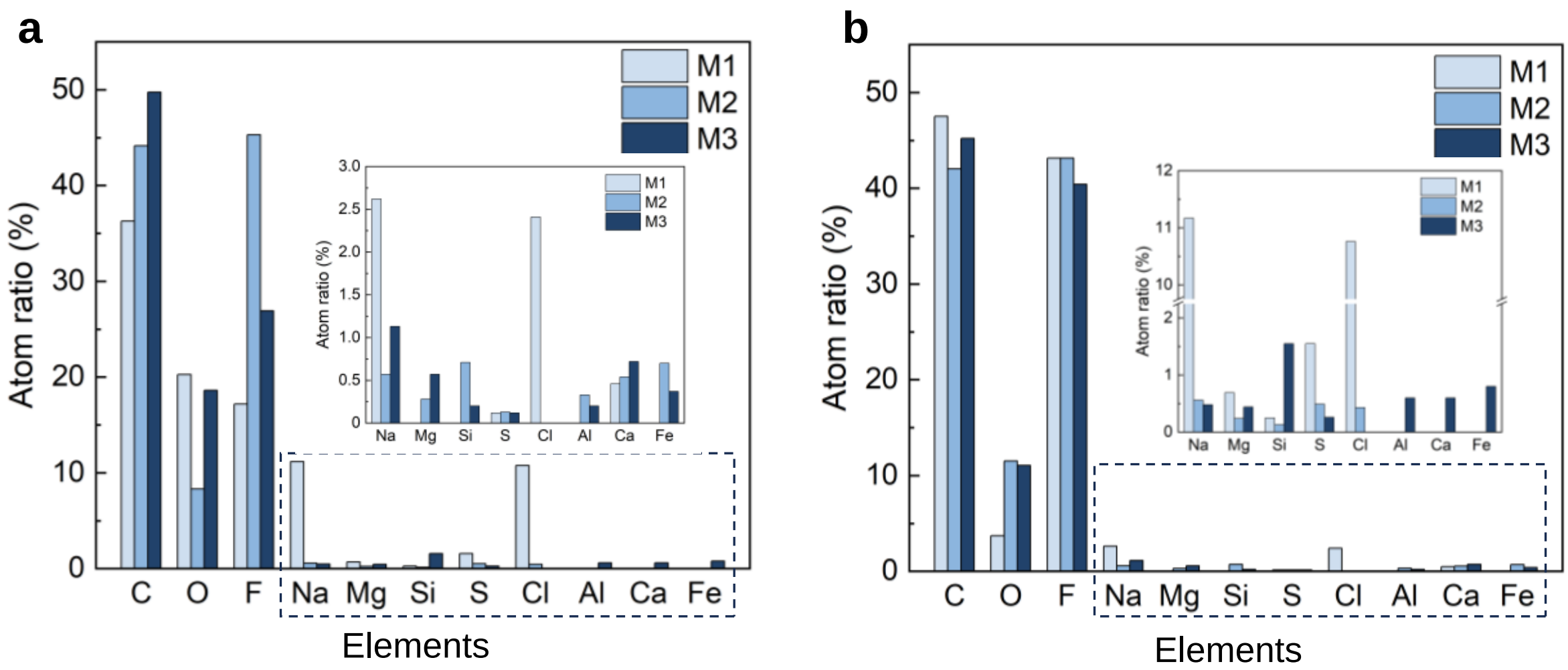

Supplementary Fig. 8 Elemental composition and proportions of inorganic elements in the fouling layer on the membrane surface at different stages (M1, M2, M3) for (a) AMBR and (b) VMBR. The dashed box indicates the range of the inset, which provides an expanded view of low-abundance elements for clearer comparison.

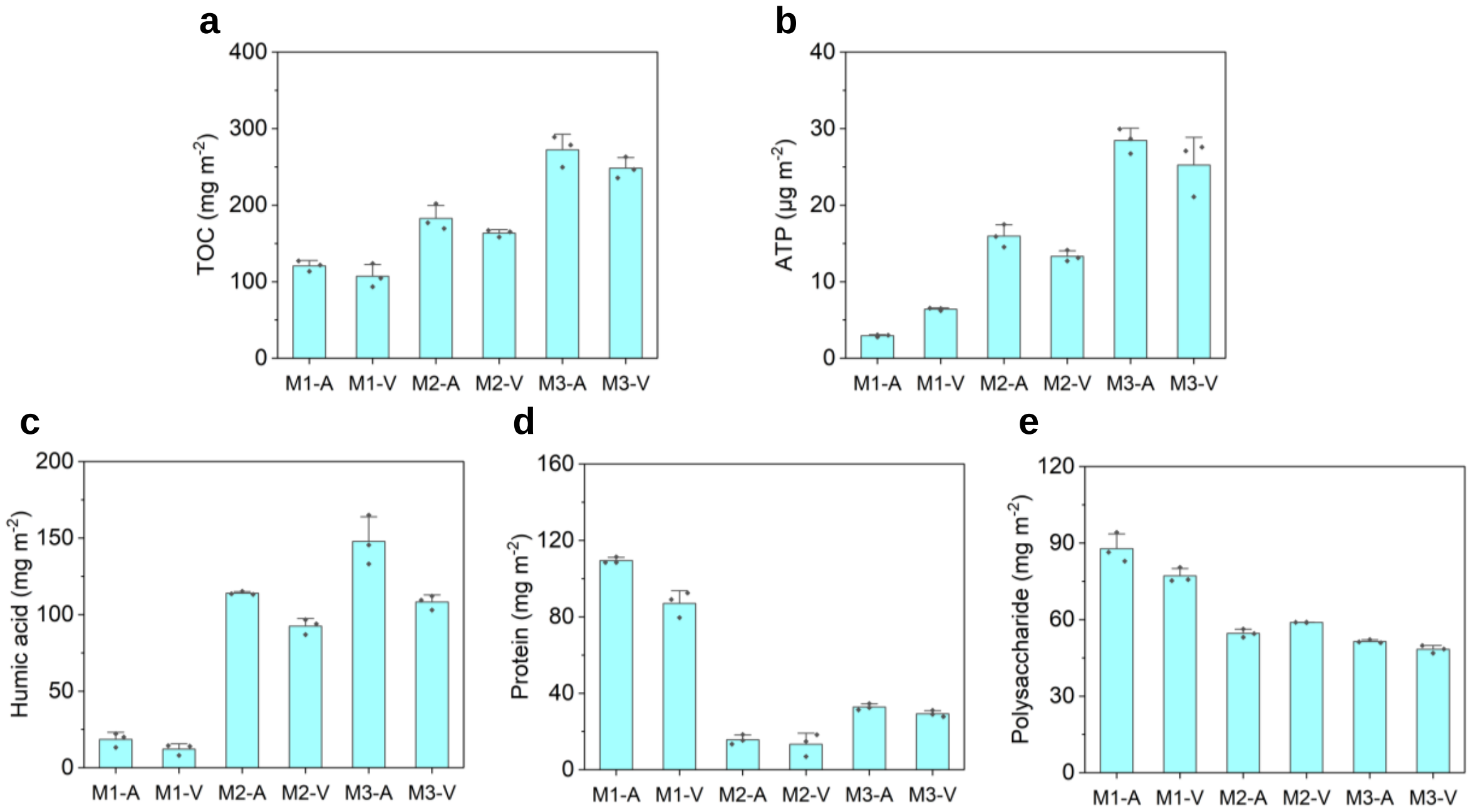

Supplementary Fig. 9 Contents of (a) TOC, (b) ATP, (c) humic substances, (d) proteins, and (e) polysaccharides in the foulant layer on the membrane surface of AMBR and VMBR at different stages (M1, M2, and M3). M1-A/M1-V represent AMBR/VMBR at stage M1; M2-A/M2-V and M3-A/M3-V are defined analogously. Error bars in the figures represent the SD (n = 3) and data are presented as mean values ± SD.

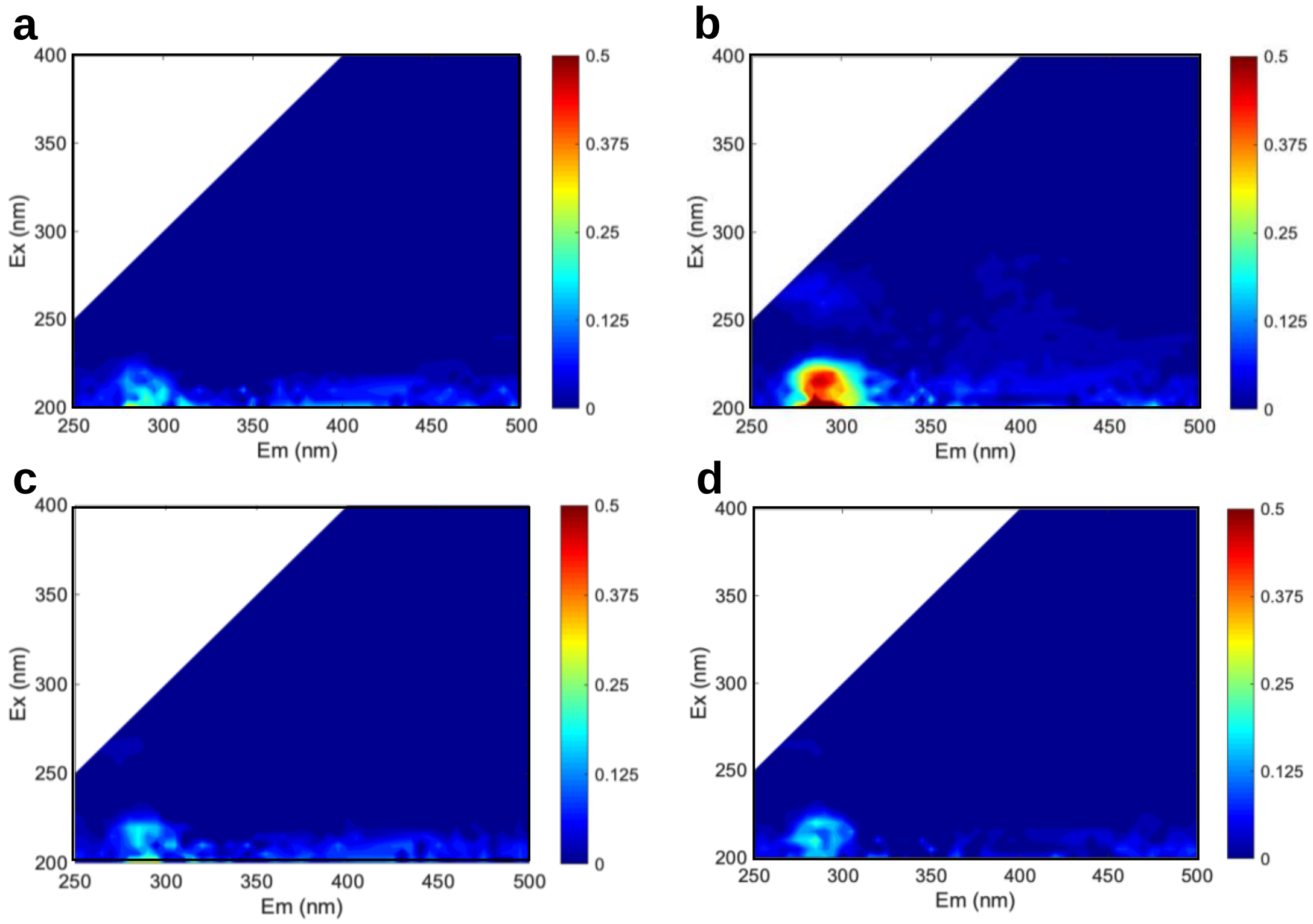

Supplementary Fig. 10 EEM fluorescence spectra of the fouling layer on the membrane surface for AMBR and VMBR at different stages (M2 and M3): (a) M2-AMBR, (b) M3-AMBR, (c) M2-VMBR and (d) M3-VMBR.

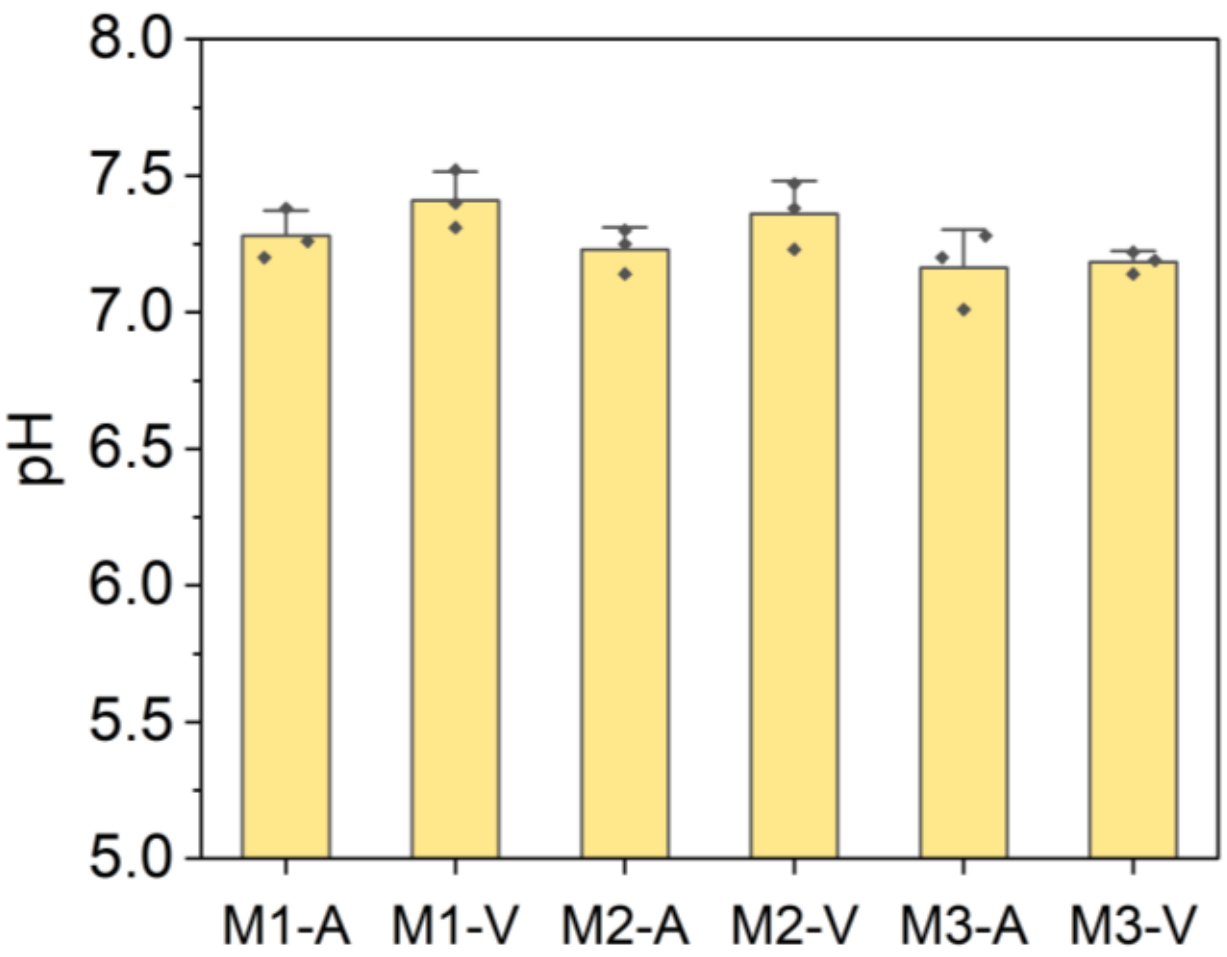

Supplementary Fig. 11 The pH of the mixed liquor in the membrane tank for AMBR and VMBR at different stages (M1, M2, and M3). M1-A/M1-V represent AMBR/VMBR at stage M1; M2-A/M2-V and M3-A/M3-V are defined analogously. Error bars in the figures represent the SD (*n* = 3) and data are presented as mean values ± SD.

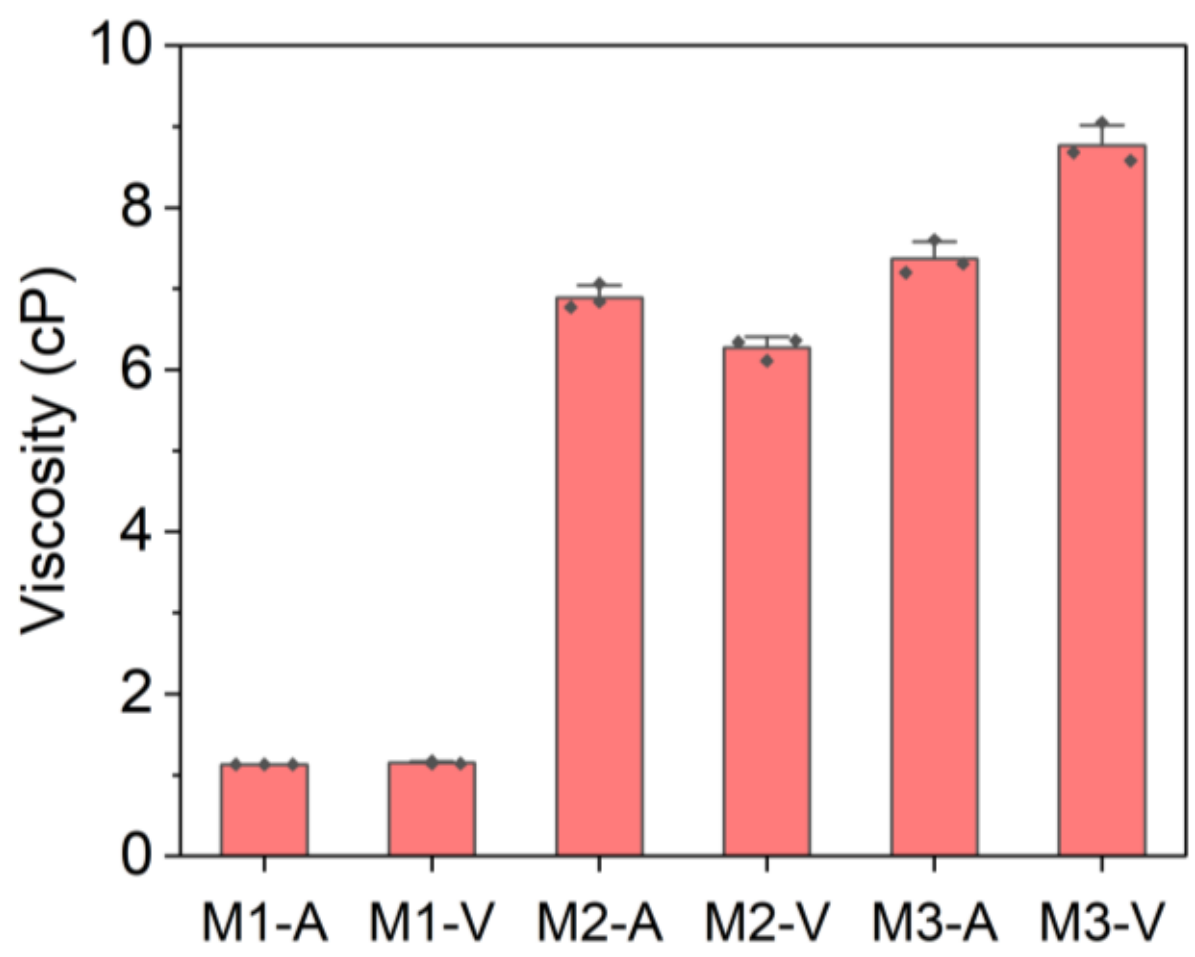

Supplementary Fig. 12 Viscosity of the mixed liquor in the membrane tank for AMBR and VMBR at different stages (M1, M2, and M3). M1-A/M1-V represent AMBR/VMBR at stage M1; M2-A/M2-V and M3-A/M3-V are defined analogously. Error bars in the figures represent the SD (n = 3) and data are presented as mean values ± SD.

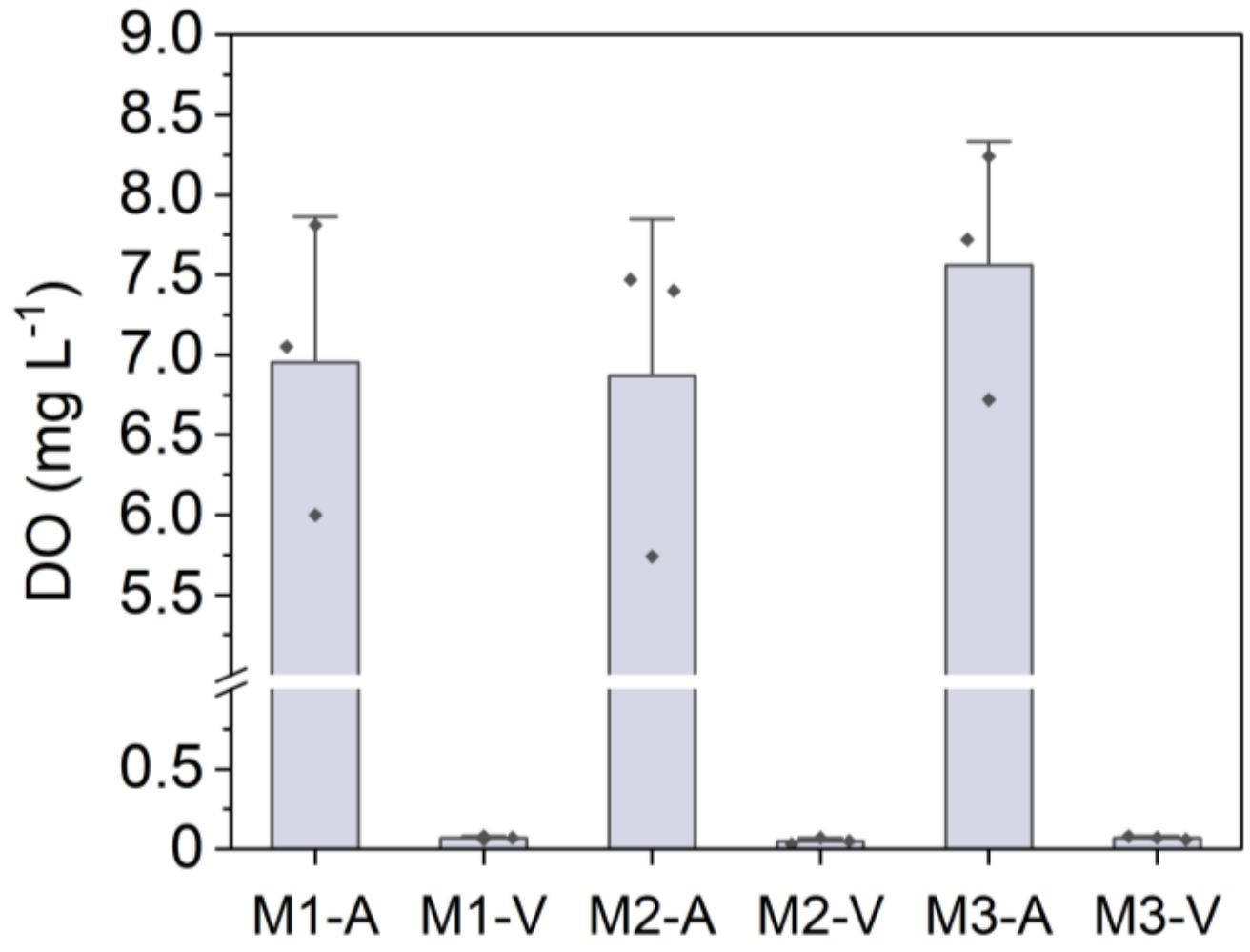

Supplementary Fig. 13 DO of the mixed liquor in the membrane tank for AMBR and VMBR at different stages (M1, M2, and M3). M1-A/M1-V represent AMBR/VMBR at stage M1; M2-A/M2-V and M3-A/M3-V are defined analogously. Error bars in the figures represent the SD (*n* = 3) and data are presented as mean values ± SD.

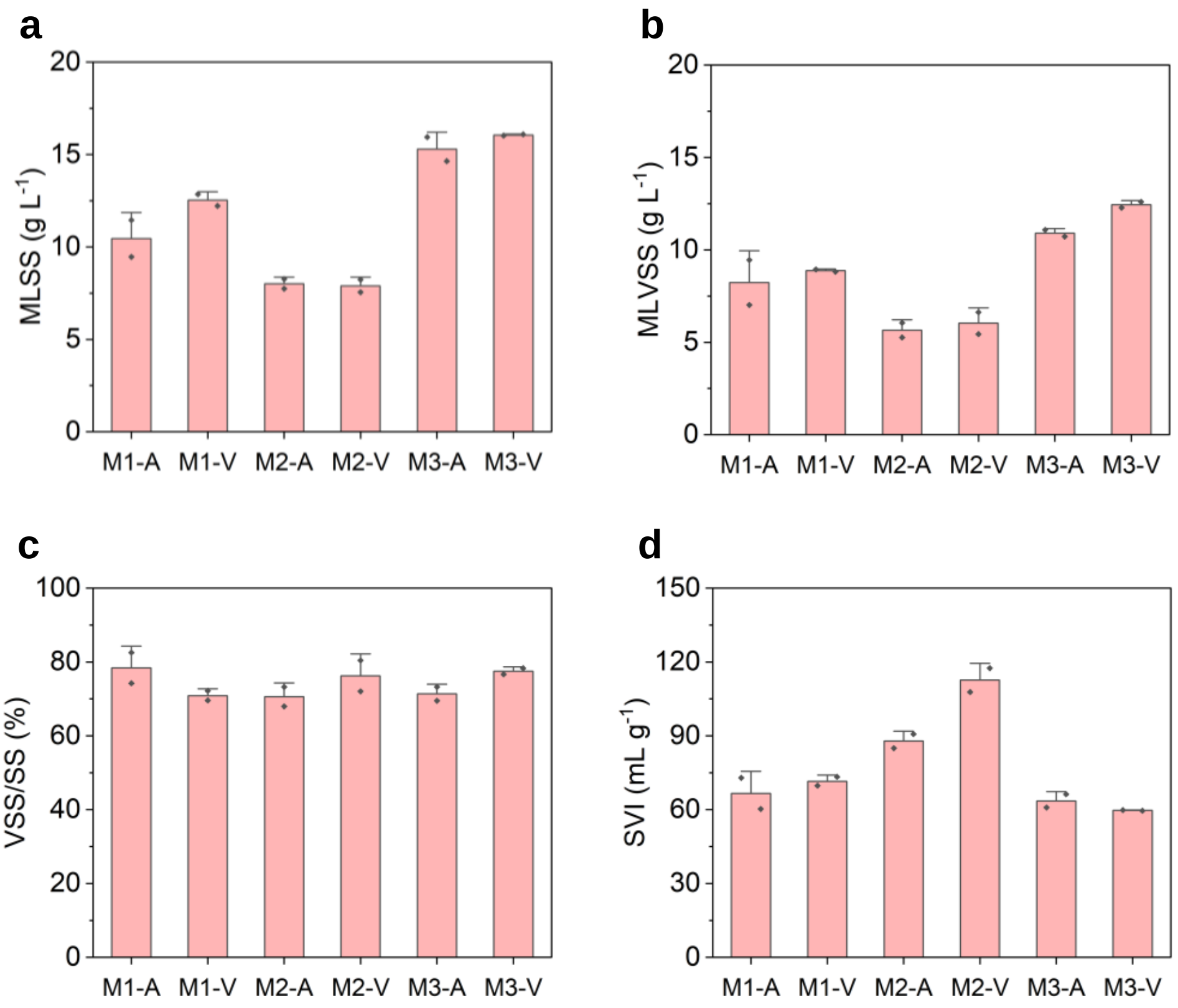

Supplementary Fig. 14 Key parameters of the mixed liquor for assessing sludge properties in AMBR and VMBR at different stages (M1, M2, and M3): (a) MLSS, (b) MLVSS, (c) VSS/SS ratio, and (d) SVI. M1-A/M1-V represent AMBR/VMBR at stage M1; M2-A/M2-V and M3-A/M3-V are defined analogously. Error bars in the figures represent the SD (n = 2) and data are presented as mean values ± SD.

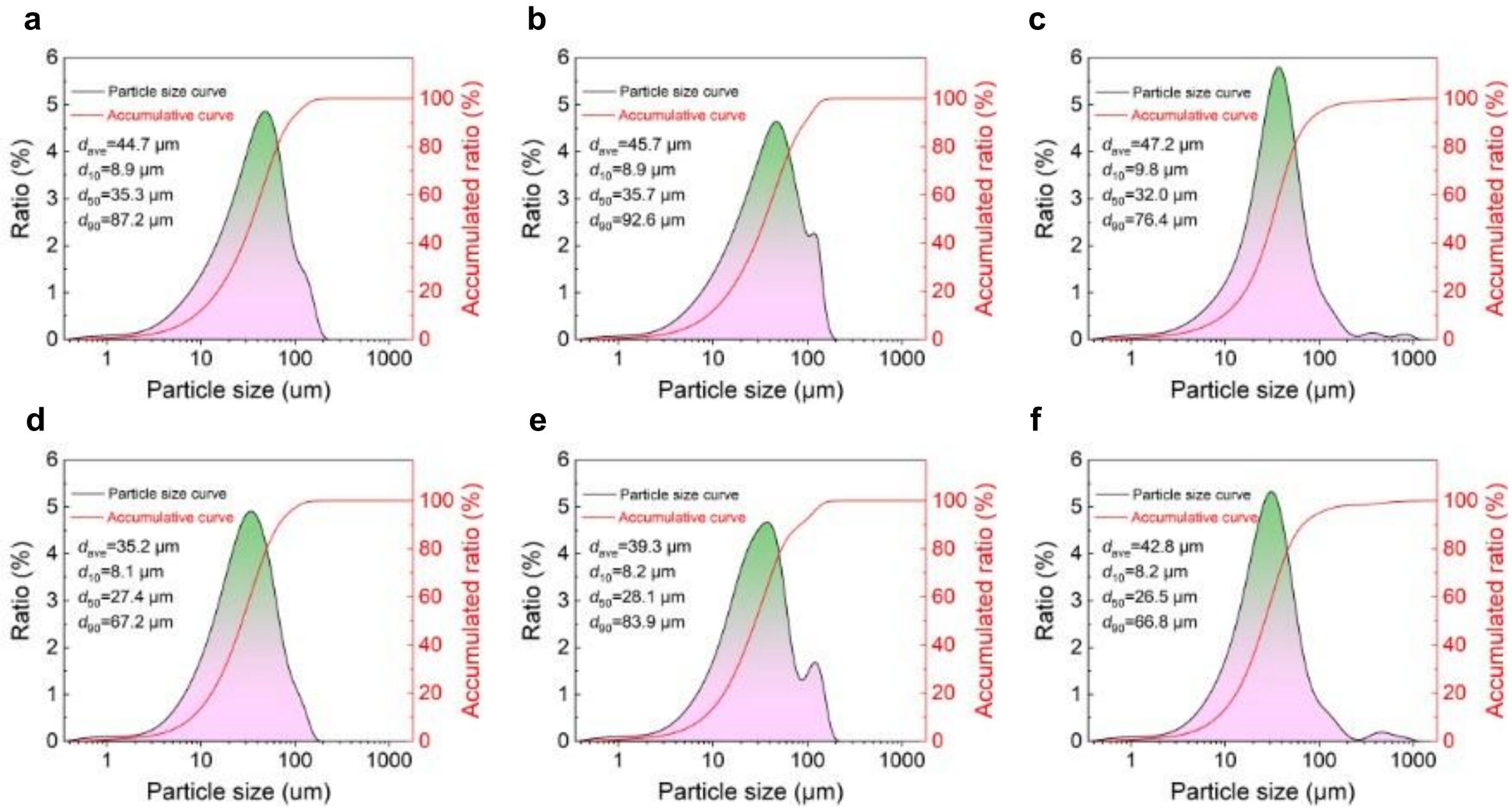

Supplementary Fig. 15 Particle size distribution (PSD) of the mixed liquor in the membrane tank of AMBR and VMBR at different stages (M1, M2, and M3): (a) M1-AMBR, (b) M2-AMBR, (c) M3-AMBR, (d) M1-VMBR, (e) M2-VMBR and (f) M3-VMBR. The notations d_{ave}, d_{10}, d_{50} and d_{90} represent the average, 10th percentile, 50th percentile (median), and 90th percentile particle sizes, respectively.

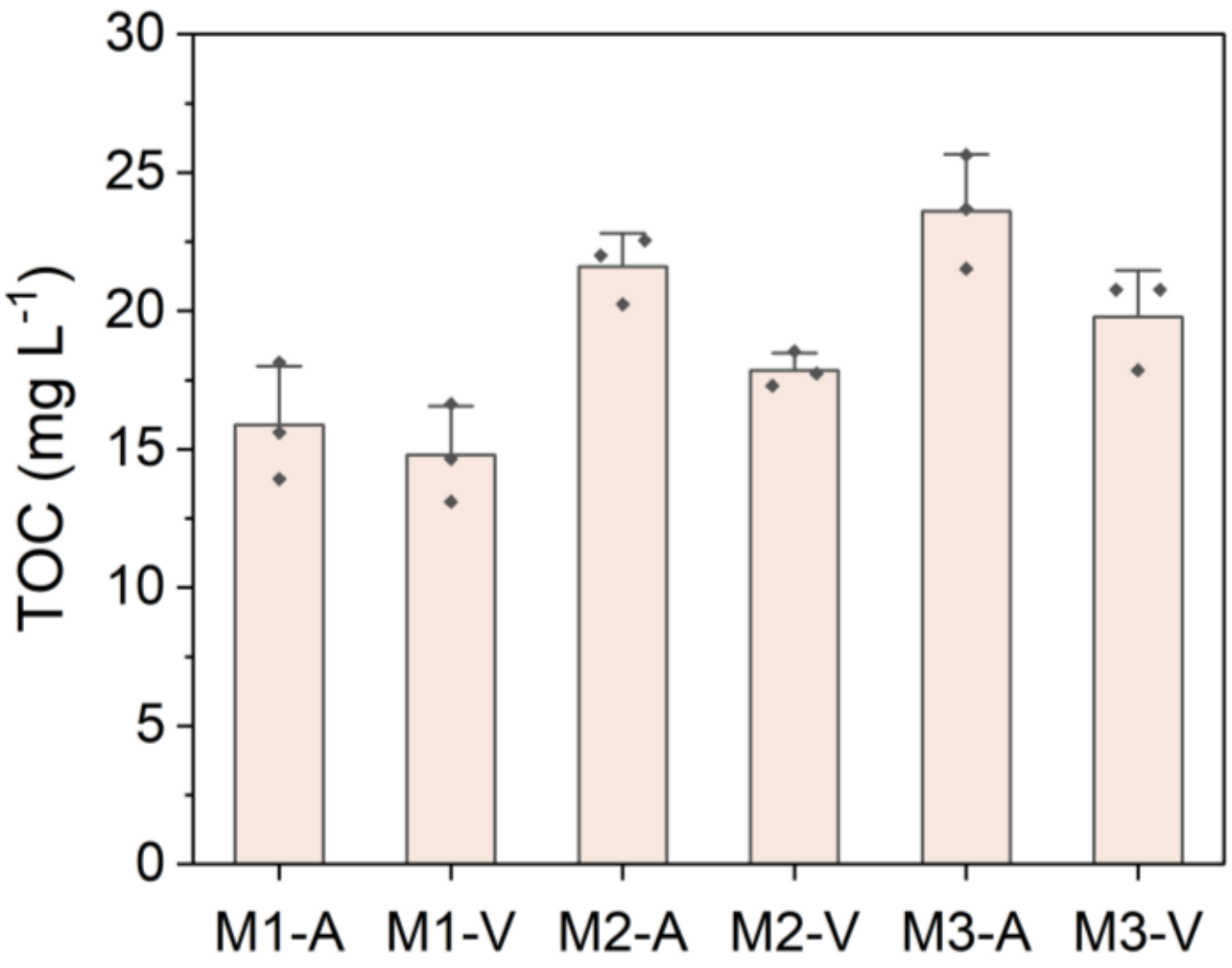

Supplementary Fig. 16 TOC content in the SMP of the mixed liquor supernatant of the AMBR and VMBR membrane tanks at different stages (M1, M2 and M3). M1-A/M1-V represent AMBR/VMBR at stage M1; M2-A/M2-V and M3-A/M3-V are defined analogously. Error bars in the figures represent the SD (*n* = 3) and data are presented as mean values ± SD.

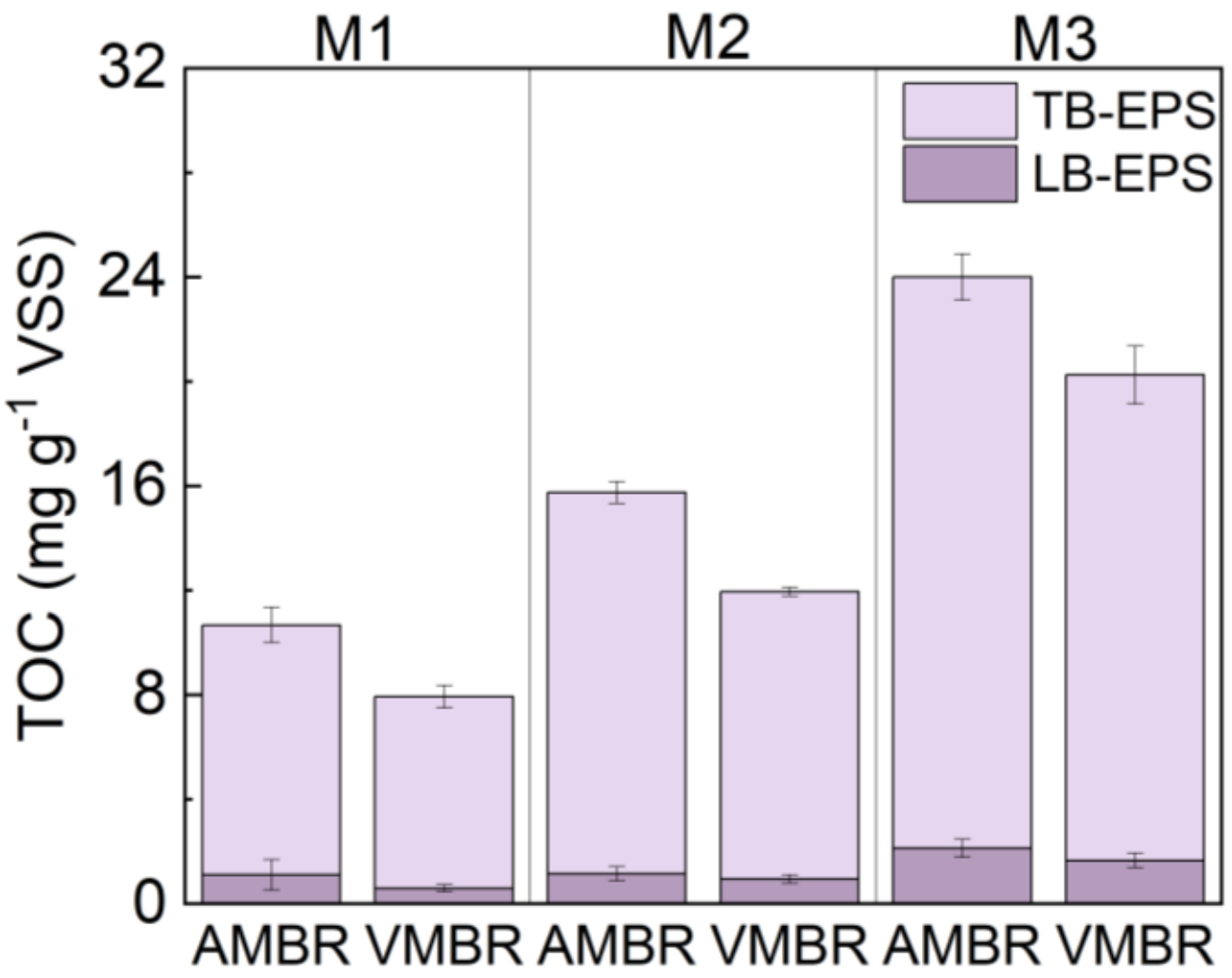

Supplementary Fig. 17 TOC content in the EPS of the sludge of the AMBR and VMBR membrane tanks at different stages (M1, M2 and M3). Error bars in the figures represent the SD (n = 3) and data are presented as mean values ± SD.

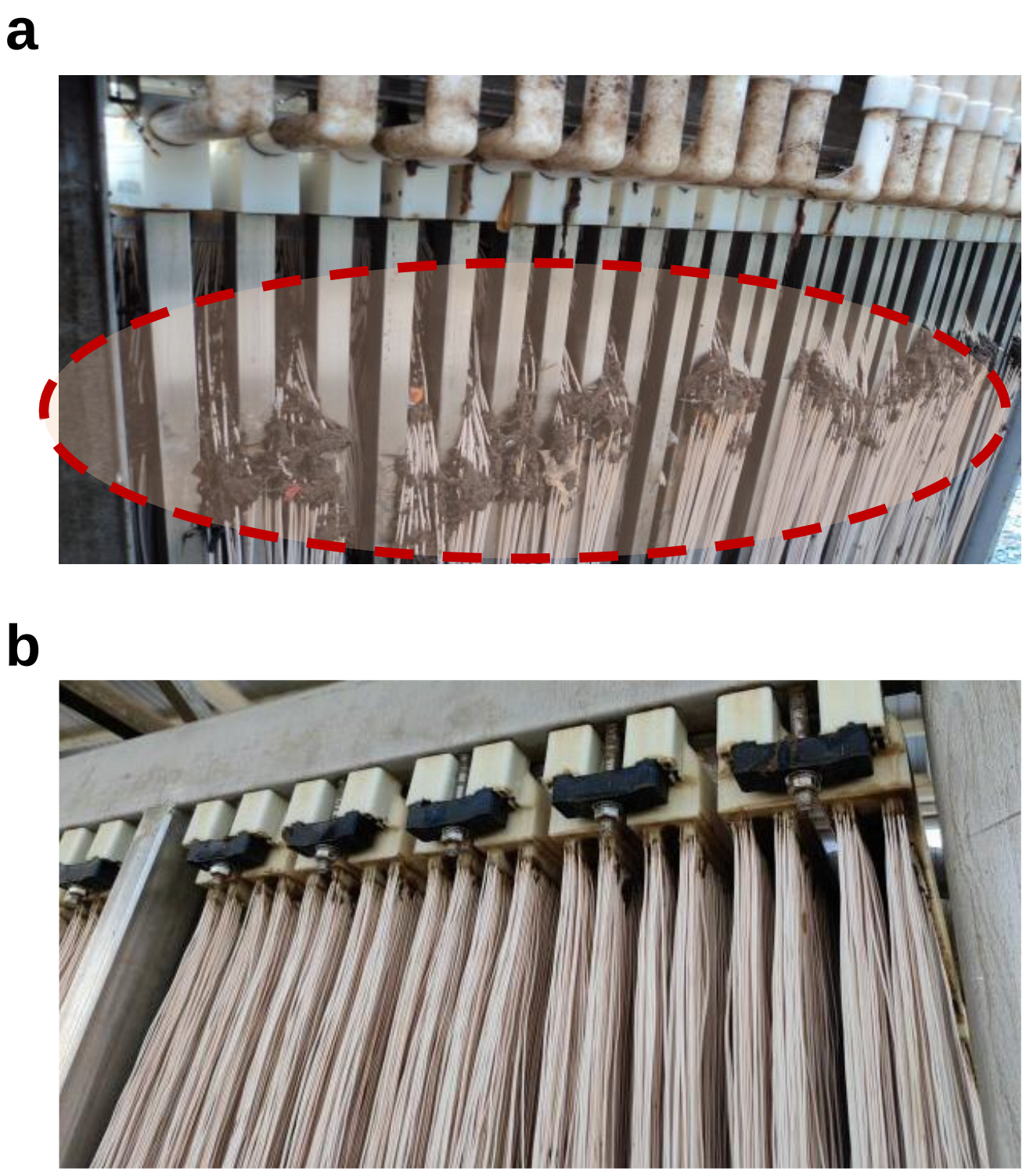

Supplementary Fig. 18 On-site photographs showing fouling at the top section of the membrane fibers in (a) AMBR and (b) VMBR.

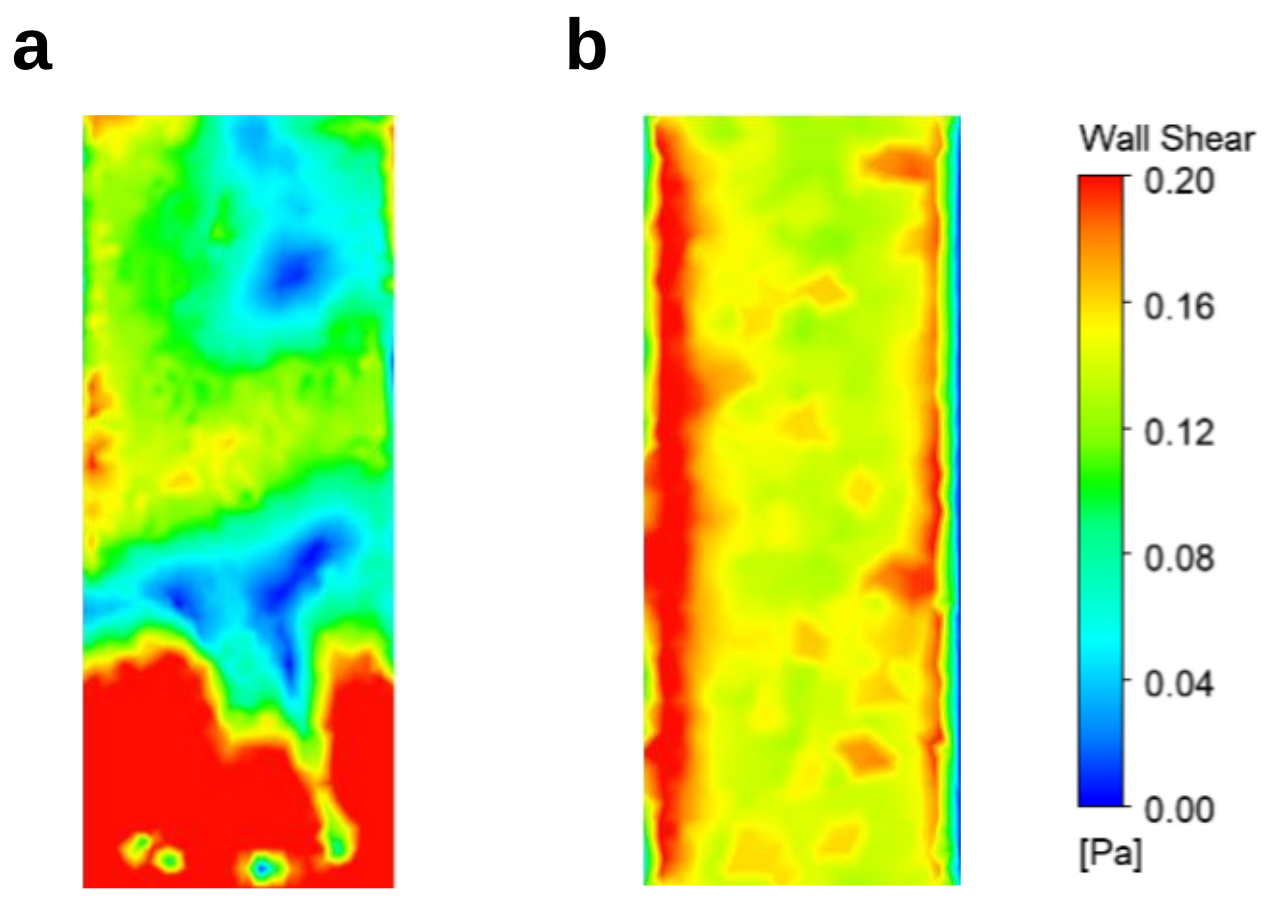

Supplementary Fig. 19 Distribution of wall shear on the membrane surface (XZ plane) of (a) AMBR and (b) VMBR.

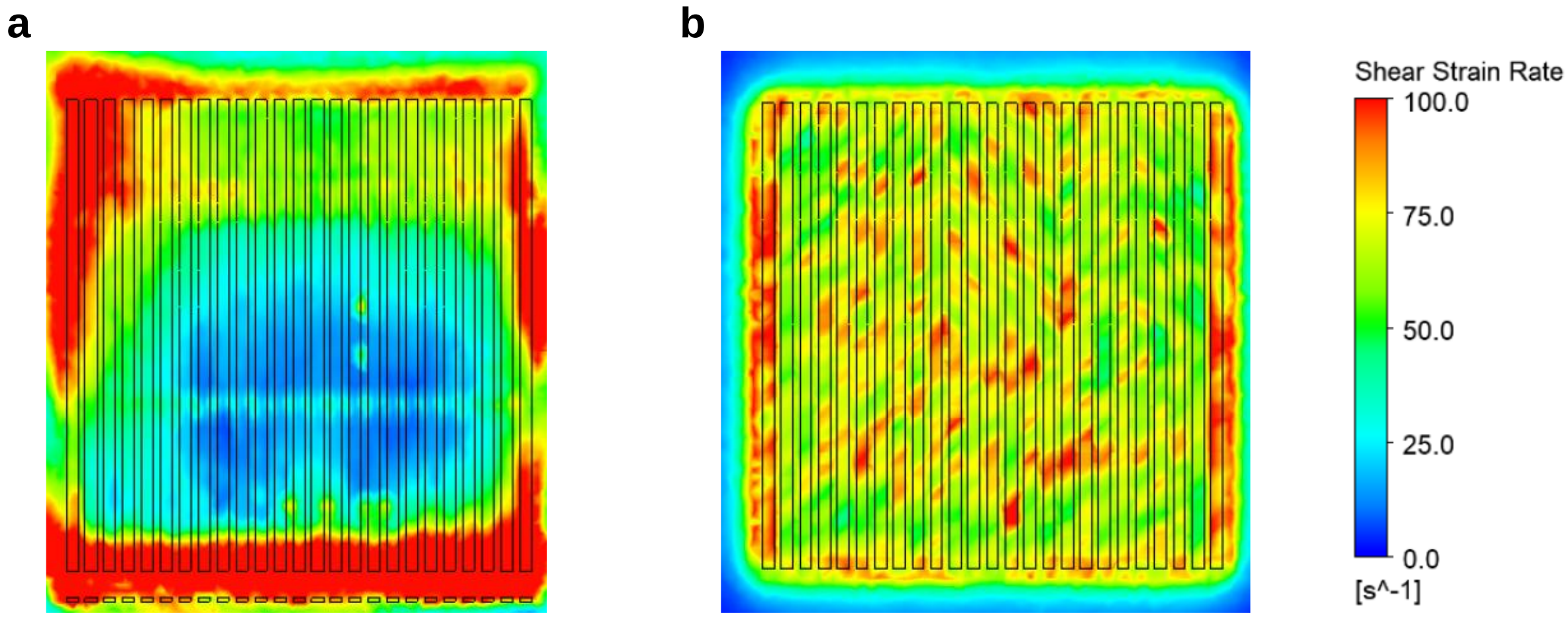

Supplementary Fig. 20 Shear strain rate profiles on the lateral plane (YZ plane) 1 mm from the membrane surface of (a) AMBR and (b) VMBR.

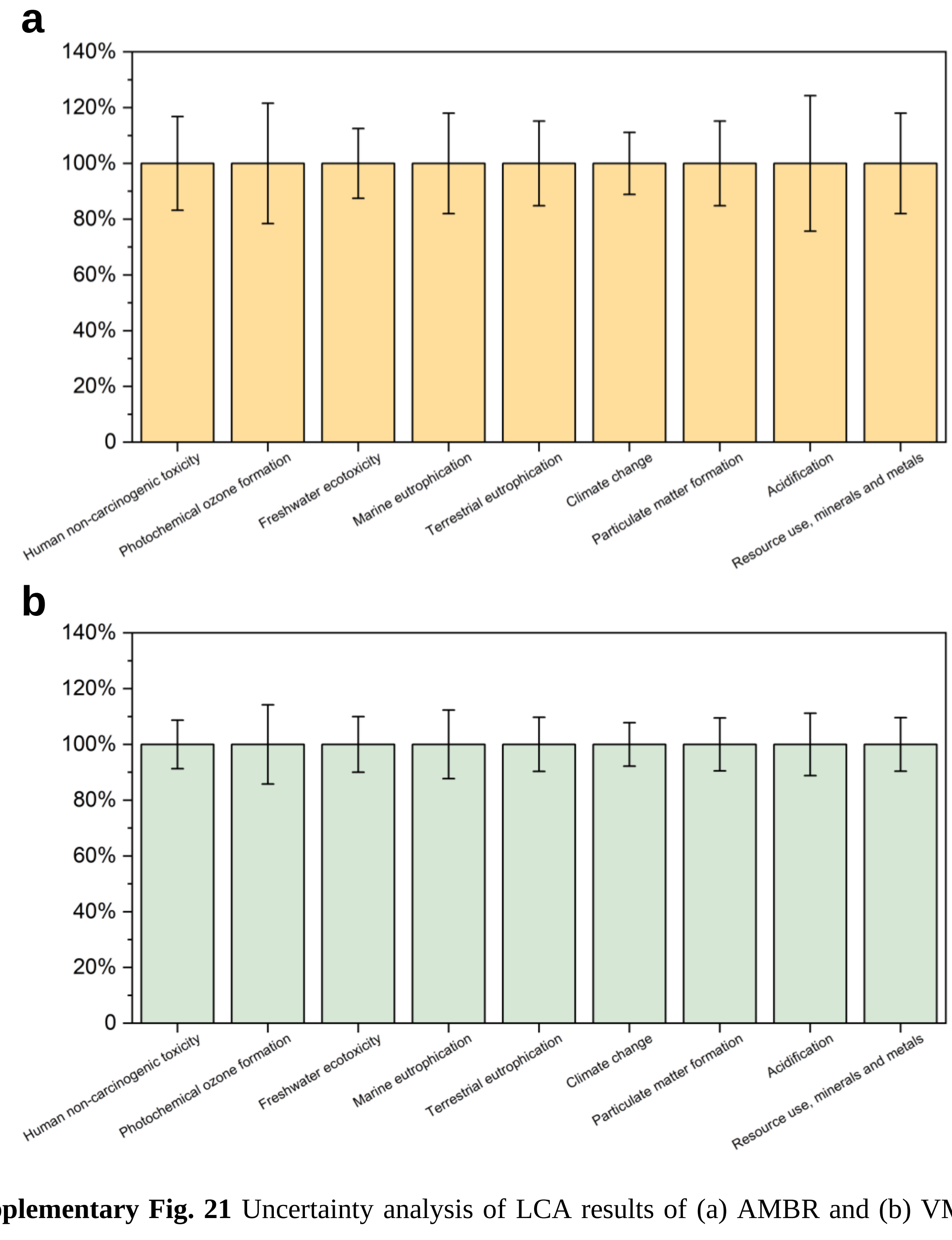

Supplementary Fig. 21 Uncertainty analysis of LCA results of (a) AMBR and (b) VMBR. The error bar represents the uncertainty range (95% confidence interval) of the result.

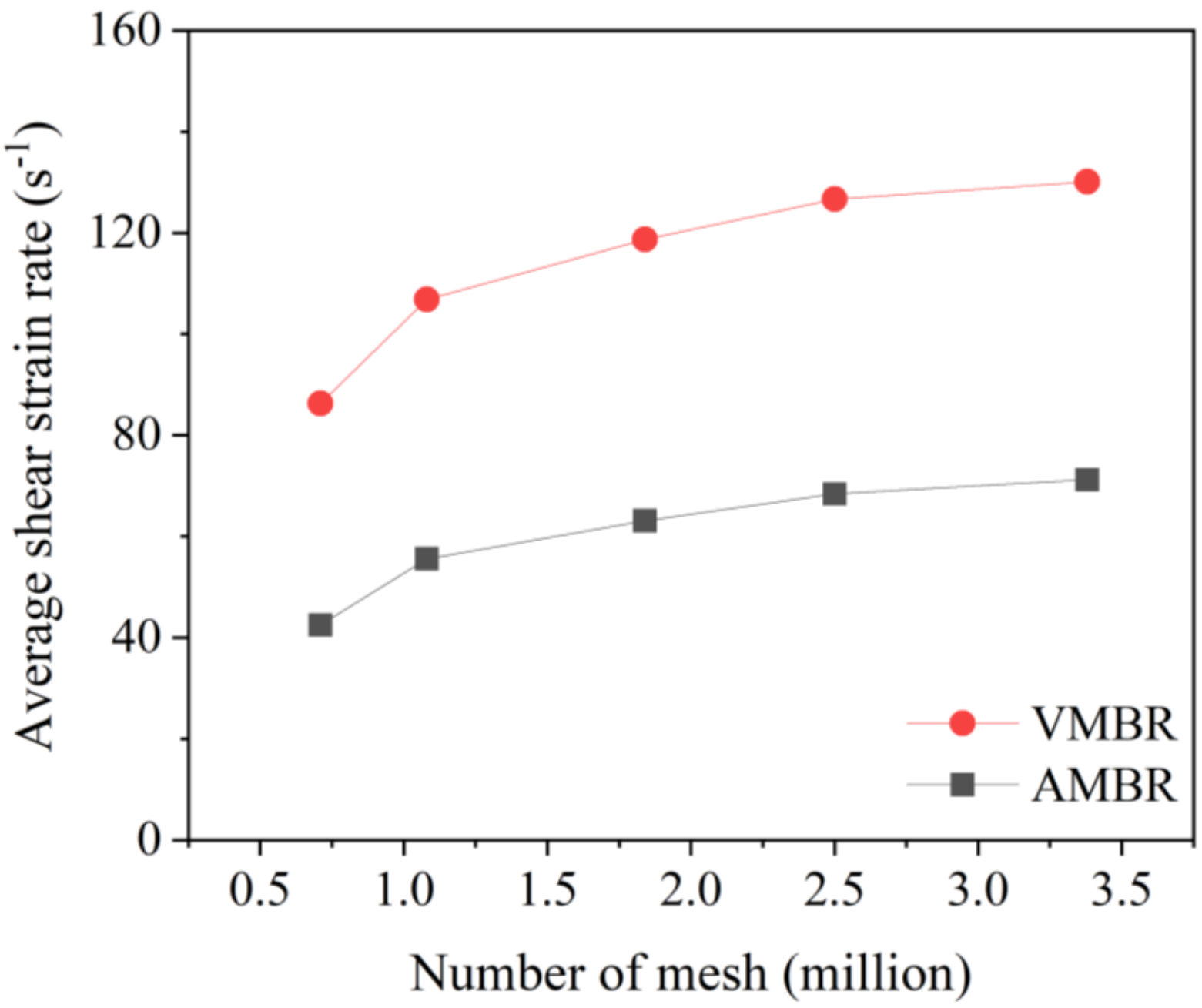

Supplementary Fig. 22 Grid independence analysis for CFD simulations of AMBR and VMBR systems, using the average shear strain rate (on the vertical plane at $x = x_2$) as the key hydrodynamic indicator. The relative differences in the computed average shear strain rate between the optimized mesh (~1.84 million elements) and a finer mesh were within 10% for both models.

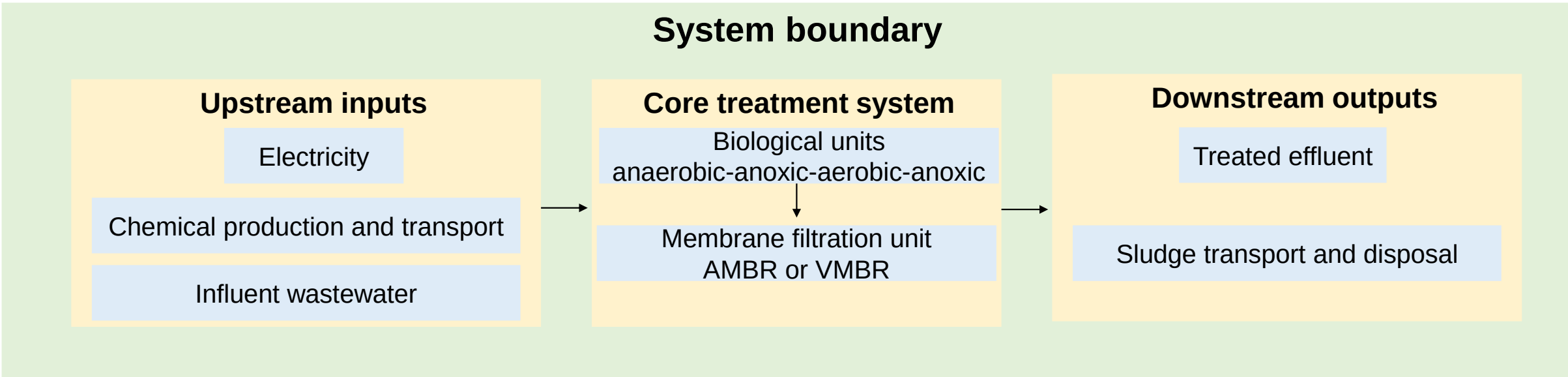

Supplementary Fig. 23 System boundary for the life cycle assessment (LCA). The assessment encompasses the operational phase, including the core biological and membrane treatment processes, key upstream inputs (electricity, chemicals), and downstream sludge disposal.

Supplementary Table 1. Life cycle environmental impacts for the AMBR and VMBR systems.

Category	Reference unit	AMBR	VMBR
Human non-carcinogenic toxicity	CTUh	1.11×10^{-11}	6.14×10^{-12}
Photochemical ozone formation	kg NMVOC eq.	2.04×10^{-6}	1.02×10^{-6}
Freshwater ecotoxicity	CTUe	6.40×10^{-5}	3.20×10^{-5}
Marine eutrophication	kg N eq.	6.58×10^{-9}	4.53×10^{-9}
Terrestrial eutrophication	mol N eq.	7.20×10^{-8}	5.44×10^{-8}
Climate change	kg CO_2 eq.	0.02467	0.02192
Particulate matter formation	disease incidence	1.03×10^{-13}	7.79×10^{-14}
Acidification	mol H^+ eq.	2.50×10^{-8}	1.72×10^{-8}
Resource use, minerals and metals	kg Sb eq.	2.63×10^{-15}	1.57×10^{-15}

Supplementary Method 1. Characterization of total organic carbon.

Total organic carbon (TOC) is determined by the subtraction method using a TOC-L Analyzer (Shimadzu, Japan). This approach quantifies total carbon (TC) and inorganic carbon (IC) separately, and the TOC concentration is derived from the difference between TC and IC. The instrument operates via high-temperature catalytic oxidation, where carbonaceous compounds in the sample are converted to carbon dioxide, which is then detected by a non-dispersive infrared sensor.

Potassium hydrogen phthalate, dried to constant weight, is used to prepare the TC standard solution, while sodium carbonate and sodium bicarbonate, both dried to constant weight, are used for the IC standard solution. A calibration series ranging from 0 to 50 mg C L^{-1} is established for both TC and IC, with linear correlation coefficients (R^2) ≥ 0.999.

Prior to measurement, the eluate is diluted 5- or 7-fold depending on the expected TOC concentration to ensure the readings fall within the linear range of the calibration curve, and then filtered through a 0.45 μm membrane filter. Each sample is analyzed in triplicate, and the average value is reported. The TOC concentration of the sample is calculated as the difference between the measured TC and IC concentrations.

Supplementary Method 2. Characterization of polysaccharides.

Polysaccharide content is determined by the phenol–sulfuric acid method using glucose as the calibration standard. In this assay, concentrated sulfuric acid hydrolyzes polysaccharides into monosaccharides, which subsequently undergo dehydration to form furfural derivatives. These derivatives then condense with phenol to produce a yellow-orange chromogen that exhibits a characteristic absorption peak at 490 nm, and the absorbance is proportional to the polysaccharide concentration in the sample.

A 5% (w/v) phenol solution is prepared fresh before use, and analytical-grade concentrated sulfuric acid is employed. D-(+)-glucose is used as the standard substance to prepare a calibration series with concentrations of 0, 2.5, 5, 10, 25, and 50 mg L^{-1}. The calibration curve is constructed by plotting absorbance against glucose concentration, with a linear correlation coefficient (R^2) of ≥ 0.998.

An appropriate volume of the eluate is mixed with the 5% (w/v) phenol solution, followed by rapid addition of concentrated sulfuric acid and thorough mixing. The mixture is allowed to react at room temperature for 20–30 min for color development. Absorbance is then measured at 490 nm using a VICTOR Nivo Multimode Plate Reader (PerkinElmer, USA). The polysaccharide concentration in the sample is calculated from the glucose calibration curve and expressed as glucose equivalents. Each sample is analyzed in triplicate.

Supplementary Method 3. Characterization of proteins.

Protein content is determined using a BCA protein assay kit (Beyotime, China). The assay is based on the biuret reaction principle: in an alkaline medium, proteins reduce Cu^{2+} ions to Cu^{+} ions, and two molecules of bicinchoninic acid (BCA) chelate with one Cu^{+} ion to form a purple-colored complex. This complex exhibits strong absorbance at 562 nm, and the absorbance intensity is directly proportional to the protein concentration in the sample.

The BCA working solution is prepared according to the kit protocol by mixing Reagent A and Reagent B at a volume ratio of 50:1. Bovine serum albumin (BSA) is used as the protein standard, and a calibration series is prepared by diluting the BSA stock solution with 0.9% (w/v) NaCl solution to final concentrations of 0, 2.5, 5, 10, 25, and 50 mg L^{-1}. The calibration curve is constructed by plotting the net absorbance against BSA concentration, with a linear correlation coefficient (R^2) of $\geq$ 0.995.

An appropriate volume of the eluate is mixed with the freshly prepared BCA working solution, and the mixture is incubated at 37 °C for 30 min. After incubation, absorbance is measured at 562 nm using a VICTOR Nivo Multimode Plate Reader (PerkinElmer, USA). The protein concentration in the sample is calculated from the BSA calibration curve and expressed as BSA equivalents. Each sample is analyzed in triplicate.

Supplementary Method 4. Characterization of humic substances.

Humic substance content is quantified by the modified Lowry method. This assay relies on the color development reaction between Folin–Ciocalteu phenol reagent and reducing substances in the sample, with absorbance measured at two wavelengths, 750 nm and 400 nm. The absorbance at 400 nm primarily reflects humic-like substances and is less affected by protein interference, making it suitable for the quantification of humic substances in complex organic mixtures.

The modified Lowry reagent, also known as the alkaline copper reagent, is prepared freshly before use by mixing Reagent A (sodium carbonate in sodium hydroxide solution) and Reagent B (copper sulfate in potassium sodium tartrate solution) at the specified ratio. The Folin–Ciocalteu reagent is diluted 1:1 (v/v) with ultrapure water prior to use. Humic acid sodium salt is used as the calibration standard, and a standard series with concentrations of 0, 2.5, 5, 10, 25, and 50 mg L^{-1} is prepared. The calibration curve for humic substances is constructed using the absorbance at 400 nm, with a linear correlation coefficient (R^2) of ≥ 0.99.

An appropriate volume of the eluate is first mixed with the modified Lowry reagent and allowed to react, followed by the addition of diluted Folin–Ciocalteu reagent for color development. The mixture is incubated at room temperature in the dark for a specified period of time. Absorbance is then measured at the corresponding wavelengths using a VICTOR Nivo Multimode Plate Reader (PerkinElmer, USA). The humic substance concentration in the sample is calculated from the humic acid calibration curve and expressed as humic acid equivalents. Each sample is analyzed in triplicate.
