# Supplementary material for: Vibrating membrane bioreactors enhance nutrient removal and energy efficiency in municipal wastewater treatment
Source: Nat Commun. 2026 Jul 20;17:8854. doi: 10.1038/s41467-026-75747-6 (PMC13500542; doi:10.1038/s41467-026-75747-6)
Supplement: Supplementary file 2 [file 41467_2026_75747_MOESM2_ESM.pdf]

Description of the Additional Supplementary Files

Supplementary Movie 1. Video of VMBR operation

Supplementary Movie 2. Video of AMBR operation
