# Supplementary material for: Vibrating membrane bioreactors enhance nutrient removal and energy efficiency in municipal wastewater treatment
Source: Nat Commun. 2026 Jul 20;17:8854. doi: 10.1038/s41467-026-75747-6 (PMC13500542; doi:10.1038/s41467-026-75747-6)
Supplement: Supplementary file 3 [file 41467_2026_75747_MOESM5_ESM.pdf]

nature portfolio

Peer Review File

Vibrating membrane bioreactors enhance nutrient removal and energy efficiency in municipal wastewater treatment

Corresponding Author: Professor Xia Huang

This file contains all reviewer reports in order by version, followed by all author rebuttals in order by version.

Version 0:

Reviewer comments:

Reviewer #1

(Remarks to the Author)
This manuscript presents a full-scale parallel comparison of vibrating membrane bioreactors (VMBR) and conventional aerated MBR (AMBR) over a long-term operation. It demonstrates VMBR's superior nutrient removal, fouling resistance, energy efficiency and lower carbon footprint, and elucidates underlying mechanisms via microbial analysis, sludge characterization and CFD simulation. This is a high-quality, impactful study with remarkable innovation and practical value. The experimental design is rigorous, the multi-scale mechanism analysis is comprehensive and in-depth, and the manuscript is well organized and written. I recommend the publication of this manuscript after a moderate revision addressing the following comments.
[1] The CFD simulation is a core part of the study to reveal the hydrodynamic mechanism of fouling control. While the grid independence test is mentioned, the complete quantitative data of key hydrodynamic parameters (e.g., average shear rate, flow velocity) under different grid numbers are suggested to be provided.
[2] The life cycle assessment (LCA) and carbon footprint analysis are key highlights of the study, but the system boundary definition and result robustness need to be further clarified. For example, the manuscript mentions membrane replacement materials in Scope 3 emissions, but does not specify whether the environmental impacts of membrane module production, replacement, and end-of-life disposal are included in the LCA framework.
[3] The VMBR's operating parameters are specified (0.5 Hz frequency, 5 cm amplitude). I suggest adding a brief explanation of the selection.
[4] Please discuss the potential challenges for large-scale promotion of VMBR, such as the mechanical stability of large-scale membrane stack vibration systems, performance regulation under extreme influent load fluctuations, and operational performance in low-temperature environments, to make the Discussion more comprehensive and objective.
[5] Line 126: The chemical dosage for phosphorus removal is provided, but the type of chemical agent is not specified.
[6] Line 183: The unit "LMH" is used for the first time here without defining its full form.
[7] Line 192-196: Please add 1-2 sentences of key quantitative results to directly support the conclusion that AMBR is more prone to inorganic and organo-inorganic composite fouling.
[8] Line 381: Please revise the phrase to either "representing 24% energy savings" or "representing a 24% energy saving".
[9] Line 470: The vibration frequency is 0.5 Hz in the Methods section, while the legend of Figure 4b marks f=5 Hz. Please carefully check and correct this typo to ensure the consistency of all key parameters throughout the manuscript.
[10] Line 520: Add the related reference to the measurement method.
[11] Line 563: What indicator was used for the grid independence test?
[12] Line 625: The parameter "BOD" appears for the first time in the main text without defining its full form.

Reviewer #2

(Remarks to the Author)
This study shows strong potential to advance municipal wastewater treatment. A key strength is the full-scale, parallel-line validation under real wastewater treatment plant conditions, where a long-term VMBR line (7,500 m3/d) is benchmarked against a conventional AMBR line. The authors report simultaneous improvements in nutrient removal, fouling resistance, and sustainability performance. Overall, the manuscript is potentially impactful. However, the interpretation and substantiation of the claimed innovation, together with several key aspects regarding the process configuration and comparability between the two full-scale lines, require substantial clarification and strengthening to make the manuscript

more rigorous and persuasive.

This is a potentially valuable full-scale engineering study, and I can see a path to publication. However, the manuscript currently overstates both novelty and mechanistic certainty. Therefore, I recommend major revision before the manuscript can be considered for publication. Please see the specific comments below:

1. The manuscript presents the two full-scale lines as parallel systems differing mainly in membrane-tank mixing (aeration in AMBR vs vibration in VMBR). However, Fig. 1 shows different recycle ratios (200% in VMBR vs 500% in AMBR), which the authors attribute to the lower DO in the VMBR membrane tank. While this may be acceptable in practice, it introduces a key confounding factor for a "parallel-line" comparison. Such differences in recycle strategy and DO conditions will inevitably influence overall treatment performance, making it unclear whether the reported advantages are mainly due to vibration itself or due to the altered DO/recycle regime enabled by VMBR. The authors should clarify this point and define the innovation boundary more explicitly.

2. To rigorously support the claimed innovation, an ideal experimental design would include a true control in which the VMBR line is operated with vibration turned off (i.e., identical configuration and operating conditions except for vibration), so that the net contribution of vibration can be directly quantified. Although this may be difficult to implement at full scale for practical reasons, the authors are strongly encouraged to supplement the study with a bench-/pilot-scale control test using the same wastewater (or representative mixed liquor) and comparable operating conditions.

3. The manuscript reports an approximately 40% reduction in chemical dosage while achieving comparable TP, which is potentially a strong and practically relevant finding. However, the current explanation is not sufficiently clear and convincing. The authors attribute the lower chemical demand to a much higher abundance of PAOs in VMBR than in AMBR (Page 9), but the manuscript does not adequately clarify what drives this PAO enrichment under the VMBR configuration (e.g., vibration-induced effects versus differences in aeration/DO regime and/or recycle strategy).
It does not demonstrate enhanced activity, flux, or pathway dominance. For a mechanistic claim at this level, I would expect stronger functional evidence such as targeted gene quantification, activity assays, phosphorus release/uptake kinetics under controlled conditions, or process-model-based mass-balance support.

4. The Methods section lacks sufficient description of the microbial community detection workflow and the associated bioinformatics/statistical analyses, which limits reproducibility and weakens the microbiology-based interpretation.

5. In the Methods, membrane fiber samples were collected only from the periphery of the AMBR and VMBR modules. Meanwhile, the CFD results suggest spatially non-uniform hydrodynamics, particularly in AMBR, which may translate into non-uniform fouling within the module. Given that fouling heterogeneity is commonly observed in full-scale systems, restricting sampling to peripheral fibers may introduce location bias and lead to position-dependent conclusions. The authors are encouraged to adopt a more representative sampling design by stratifying samples by module height (top/middle/bottom) and bundle position (periphery vs center), with appropriate replicates, and to compare fouling quantity/composition and filtration resistance accordingly.
In addition, organic foulants were eluted using 0.5% NaClO, which is an aggressive oxidant and can alter proteins, polysaccharides, and humic-like material. This makes the quantitative compositional comparison less convincing, as the cleaning efficiency by using 0.5% NaClO could be largely different between AMBR and VMBR. These limitations should be acknowledged explicitly, and the claims about foulant composition should be moderated unless the authors can provide validation that the extraction approach preserves the analytes of interest.

6. The Methods describe the VMBR vibration conditions as 0.5 Hz with an amplitude of 5 cm, whereas the caption/text associated with Fig. 4 states 5 Hz and 50 mm. The authors should carefully re-check and verify the correct operating parameters and ensure consistency across the Methods, figures/captions, and any related analyses (e.g., CFD and energy calculations).

7. In the CFD model, the mixed liquor was assigned a density of 1000 kg/m3 and a dynamic viscosity of 0.001 Pa·s, essentially treating it as water. However, the viscosity of MBR mixed liquor is typically substantially higher than that of water and can vary with MLSS and shear rate. Using a water-like viscosity may bias the predicted flow field and shear distribution. The authors should incorporate measured (or otherwise well-justified) mixed liquor viscosity under the relevant operating conditions and discuss how this assumption affects the CFD-based conclusions.

8. The Methods state that an online NaClO cleaning was conducted on a weekly basis. The authors should clearly annotate the cleaning times on the TMP and flux profiles, so that the post-cleaning recovery and cleaning effectiveness can be directly assessed and compared between AMBR and VMBR.

9. The Methods state that AMBR and VMBR have been stably operated for ~4 years, but the manuscript mainly presents a much shorter monitoring period. The authors should consider including longer-term performance/stability data, which would be highly valuable for evaluating the practical engineering applicability of VMBR.

10. The novelty needs to be repositioned much more carefully. The manuscript frames the study as if the benefits of reciprocating/vibrating submerged membranes are being established here for the first time, yet the paper itself cites prior laboratory- and pilot-scale studies already showing improved fouling control, energy savings, and enhanced nutrient removal in reciprocating/vibrating MBRs. The real advance here appears to be the full-scale side-by-side implementation, not the discovery of the core principle. I therefore encourage the authors to substantially tone down the conceptual novelty

claim and reframe the manuscript around scale-up validation, practical integration into a full biological train, and limits of transferability.

11. The antifouling improvement is promising but should be interpreted more cautiously. The reported average flux difference is relatively modest (15.0 versus 16.1 LMH), and both systems still relied on weekly online cleaning and annual offline cleaning. The lower TMP-rise rate in VMBR is meaningful, but the manuscript presently extrapolates this into a very broad antifouling narrative without sufficient long-term evidence on irreversible resistance buildup, recovery after repeated cleaning cycles, membrane integrity, or actual extension of service life. The antifouling claims would be much stronger if supported by longer post-cleaning trajectories or resistance partitioning.

12. The carbon-emission and LCA claims are broader than the underlying evidence base. Emissions seem not directly measured. Rather, N_2O and CH_4 are estimated from influent–effluent balances and default emission factors. The LCA then reports relative reductions with the AMBR normalized to 100%, which makes it harder to judge the magnitude and uncertainty of the absolute impacts. Importantly, the paper links lower resource use partly to extended membrane lifespan, but no membrane-life data are actually presented. These sustainability conclusions should therefore be stated more cautiously, and uncertainty analysis would greatly strengthen this section.

13. The translational scope remains narrower than the discussion suggests. Although this is full scale, it is still effectively a single-plant case study. The direct comparative window is 120 days after cleaning, within one site configuration and one influent context. The manuscript does not yet adequately address broader deployment questions such as vibration-system reliability, maintenance burden, retrofit feasibility, mechanical durability, or robustness under different wastewater compositions and seasonal regimes. Those issues are especially important if the authors wish to argue that VMBR is a key next-generation wastewater technology.

Reviewer #3

(Remarks to the Author)
I co-reviewed this manuscript with one of the reviewers who provided the listed reports. This is part of the Nature Communications initiative to facilitate training in peer review and to provide appropriate recognition for Early Career Researchers who co-review manuscripts.

Version 1:

Reviewer comments:

Reviewer #1

(Remarks to the Author)
I have reviewed the revised manuscript and the authors' responses to my previous comments. I confirm that the paper has been revised thoroughly, with all review comments addressed point by point in the response letter and corresponding changes made in the manuscript. The revisions have significantly improved the quality of the work. I believe the manuscript in its current form has reached an acceptable standard for publication.

Reviewer #2

(Remarks to the Author)
The authors have addressed all my comments

Reviewer #3

(Remarks to the Author)
I co-reviewed this manuscript with one of the reviewers who provided the listed reports. This is part of the Nature Communications initiative to facilitate training in peer review and to provide appropriate recognition for Early Career Researchers who co-review manuscripts.

Open Access This Peer Review File is licensed under a Creative Commons Attribution 4.0 International License, which permits use, sharing, adaptation, distribution and reproduction in any medium or format, as long as you give appropriate credit to the original author(s) and the source, provide a link to the Creative Commons license, and indicate if changes were made.
In cases where reviewers are anonymous, credit should be given to 'Anonymous Referee' and the source.
The images or other third party material in this Peer Review File are included in the article's Creative Commons license, unless indicated otherwise in a credit line to the material. If material is not included in the article's Creative Commons license and your intended use is not permitted by statutory regulation or exceeds the permitted use, you will need to obtain permission directly from the copyright holder.
To view a copy of this license, visit https://creativecommons.org/licenses/by/4.0/

Manuscript Number: NCOMMS-26-008551A
Manuscript Title: Vibrating membrane bioreactors unlock synergistic enhancement of nutrient removal and energy efficiency in full-scale municipal wastewater treatment.
Article Type: Research paper
Reply to Reviewer comments, NCOMMS-26-008551A

Reviewers' comments:

-Reviewer 1

This manuscript presents a full-scale parallel comparison of vibrating membrane bioreactors (VMBR) and conventional aerated MBR (AMBR) over a long-term operation. It demonstrates VMBR's superior nutrient removal, fouling resistance, energy efficiency and lower carbon footprint, and elucidates underlying mechanisms via microbial analysis, sludge characterization and CFD simulation. This is a high-quality, impactful study with remarkable innovation and practical value. The experimental design is rigorous, the multi-scale mechanism analysis is comprehensive and in-depth, and the manuscript is well organized and written. I recommend the publication of this manuscript after a moderate revision addressing the following comments.

Reply:

We sincerely appreciate the reviewer's positive evaluation and insightful suggestions on our work. The detailed response and revisions based on your questions and suggestions are listed below. The line numbers in the response refer to the revised manuscript with changes marked.

1. The CFD simulation is a core part of the study to reveal the hydrodynamic mechanism of fouling control. While the grid independence test is mentioned, the complete quantitative data of key hydrodynamic parameters (e.g., average shear rate, flow velocity) under different grid numbers are suggested to be provided.

Reply:

We sincerely thank the reviewer for this rigorous and constructive comment, which is critical for improving the reproducibility and accuracy of our CFD simulation. We fully acknowledge that the original manuscript only stated the final result of the grid independence study but lacked the supporting quantitative data of key hydrodynamic parameters. To address this, we have supplemented the complete quantitative results of core evaluation parameter (average shear strain rate) under different grid numbers, and clearly elaborated the basis for selecting the optimized mesh of ~1.84 million elements in the manuscript, as "A grid independence study was performed using average shear strain rate as the key indicator, with a relative difference of less than 10% between the optimized mesh (~1.84 million elements) and a finer mesh (Supplementary Fig. 22), which meets the CFD accuracy requirement while balancing computational cost." in Line 699-704.

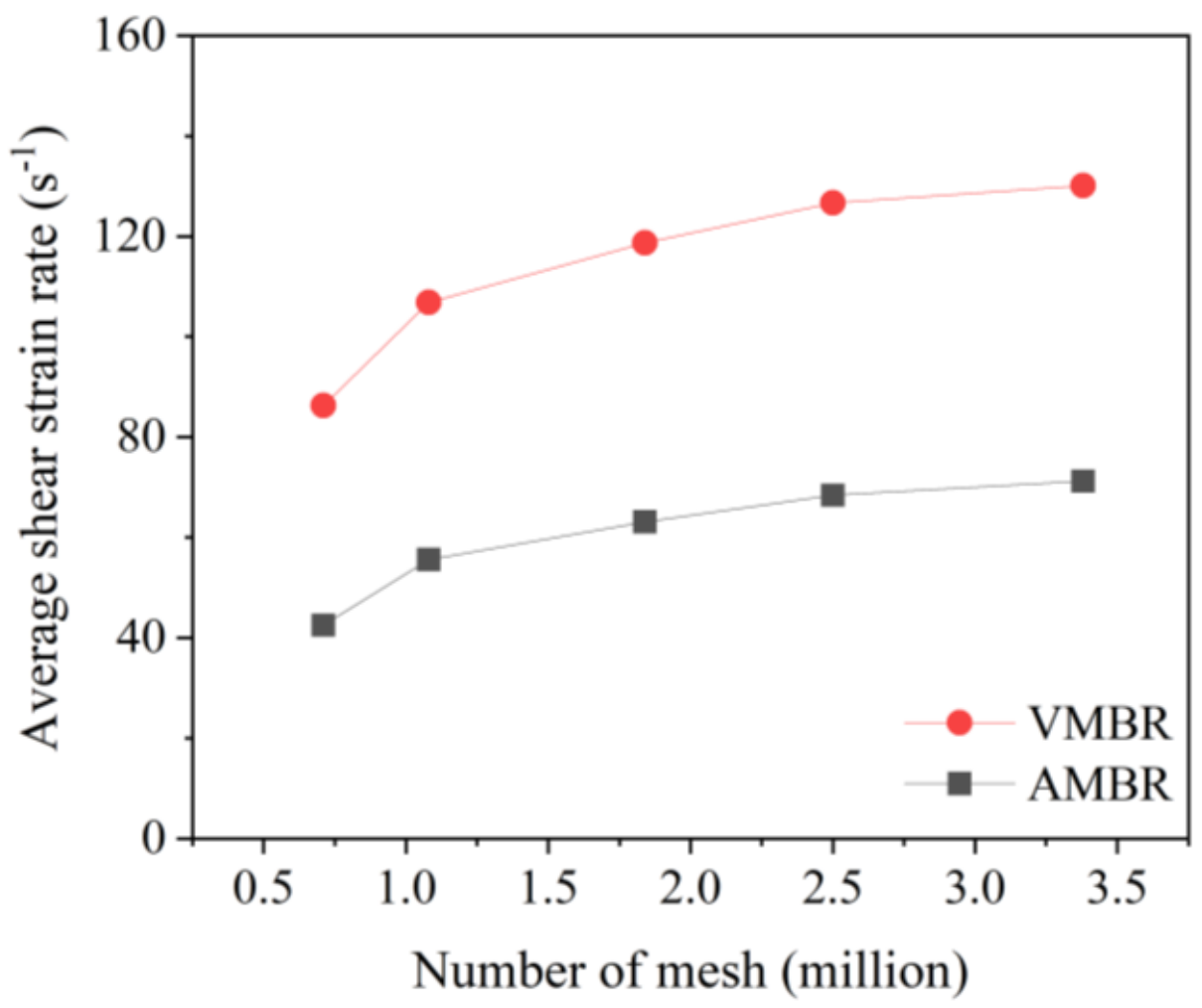

Supplementary Fig. 22 The grid independence analysis for CFD simulations of AMBR and VMBR systems, using the average shear strain rate (on the vertical plane at $x = x_2$) as the key hydrodynamic indicator. The relative differences in the computed average shear strain rate between the optimized mesh (~1.84 million elements) and a finer mesh were within 10% for both models.

2. The life cycle assessment (LCA) and carbon footprint analysis are key highlights of the study, but the system boundary definition and result robustness need to be further clarified. For example, the manuscript mentions membrane replacement materials in Scope 3 emissions, but does not specify whether the environmental impacts of membrane module production, replacement, and end-of-life disposal are included in the LCA framework.

Reply:

Thank you for this rigorous and constructive comment, which helps us correct a critical descriptive error in our manuscript. The statement mentioning "membrane replacement materials are included in Scope 3 emissions" in the original Methods section is an incorrect description; in fact, membrane replacement materials, along with the full life cycle environmental impacts of membrane module production, operational replacement, and end-of-life disposal, were not included in our LCA framework. This is because our LCA mainly focused on the operational phase, with the core objective of quantifying the relative carbon emission difference between the two parallel systems with identical membrane configurations, to isolate the impact of different fouling control strategies. Besides, no membrane replacement occurred during the operational phase. Accordingly, we have revised the manuscript to correct the incorrect Scope 3 description, and clearly define the LCA's operational phase system boundary, as "The system boundary of LCA followed a framework focused on the operational phase of the systems (detailed in Supplementary Fig. 23, which encompassed: (1) direct operational inputs and emissions, including energy consumption for biological treatment, pumping, and membrane fouling control, as well as direct emissions of N_2O and CH_4; (2) relevant upstream and downstream processes, including the production and transport of chemicals ($FeCl_3$) for phosphorus removal, upstream emissions from regional grid electricity generation (predominantly fossil fuel-based), and the transport

and incineration of dewatered sludge. Environmental impacts related to membrane modules (including production, replacement, and end-of-life disposal) are explicitly excluded from the system boundary and accounting scope." in Line 778-790.

3. The VMBR's operating parameters are specified (0.5 Hz frequency, 5 cm amplitude). I suggest adding a brief explanation of the selection.

Reply:

Thank you for this constructive suggestion. The selection of VMBR's operating parameters (0.5 Hz frequency, 5 cm amplitude) was based on full-scale engineering-oriented considerations: the safe long-term operating range of the vibration device, and the optimal balance between fouling control efficiency and operating energy consumption verified by pre-commissioning tests. We have supplemented a brief explanation for the selection of the VMBR's operating parameters in the Methods section, as "The VMBR system was actuated by a horizontal reciprocating motion, configured to operate at a frequency of 0.5 Hz and an amplitude of 5 cm (Supplementary Movie 1). These parameters were selected through full-scale pre-commissioning to balance fouling control efficiency and long-term mechanical stability." in Line 584-587.

4. Please discuss the potential challenges for large-scale promotion of VMBR, such as the mechanical stability of large-scale membrane stack vibration systems, performance regulation under extreme influent load fluctuations, and operational performance in low-temperature environments, to make the Discussion more comprehensive and objective.

Reply:

Thank you for this suggestion. We have supplemented a dedicated section in the Discussion to systematically elaborate the key potential challenges for the large-scale promotion of VMBR technology, as "While this full-scale parallel study demonstrates the promising performance of VMBR, broader translational conclusions still require further multi-scenario validation. The full-life-cycle mechanical reliability, maintenance burden, retrofit feasibility for existing MBR facilities, and structural durability of large-scale vibration systems have yet to be comprehensively evaluated over the full membrane design service life (typically 5-8 years). Additionally, carbon emission accounting in this study relies on standardized IPCC emission factors, and future on-site continuous monitoring of greenhouse gas emissions will further improve the accuracy of carbon accounting. Moreover, the operational robustness of VMBR under extreme wastewater quality fluctuations, shock influent loading events, and long-term variable climatic conditions also warrants further investigation. These follow-up studies would help facilitate the broader engineering application of VMBR as a low-carbon, energy-efficient wastewater treatment technology." in Line 538-550.

5. Line 126: The chemical dosage for phosphorus removal is provided, but the type of chemical agent is not specified.

Reply:

Thank you for this comment. The type of chemical agent for phosphorus removal is

$FeCl_3$. We have supplemented the information in both the main text as "the chemical dosage ($FeCl_3$) required for phosphorus removal" in Line 140, and the figure caption of Fig. 1g as "**g,** Chemical dosage ($FeCl_3$) for phosphorus removal in AMBR and VMBR systems." in Line 211.

6. Line 183: The unit "LMH" is used for the first time here without defining its full form.

Reply:

Thank you for the correction. We have provided the full form of LMH here, as "Over the full monitoring period, the AMBR recorded an average permeate flux of 12.3 ± 1.5 $L/(m^2 \cdot h)$ (LMH), while the VMBR maintained a significantly higher average flux of 15.7 ± 2.3 LMH ($p < 0.001$, Supplementary Fig. 6a)." in Line 230-233.

7. Line 192-196: Please add 1-2 sentences of key quantitative results to directly support the conclusion that AMBR is more prone to inorganic and organo-inorganic composite fouling.

Reply:

Thank you for this suggestion. We have added a quantitative statement to support the claim here, as "Specifically, the AMBR foulant contains higher proportions of inorganic fouling-related elements (e.g., Si: 1.55%, Al: 0.60%, Ca: 0.60%, Fe: 0.80%) alongside a higher organic carbon content (49.75%), while the VMBR foulant showed significantly reduced inorganic element levels (e.g., Si: 0.20%, Al: 0.20%, Ca: 0.72%, Fe: 0.37%) and a comparable organic carbon fraction (45.21%), confirming less severe inorganic and composite fouling (Supplementary Fig. 8)." in Line 252-257.

8. Line 381: Please revise the phrase to either "representing 24% energy savings" or "representing a 24% energy saving".

Reply:

Thank you for the correction. We have revised it to "representing a 24% energy saving" to ensure grammatical accuracy.

9. Line 470: The vibration frequency is 0.5 Hz in the Methods section, while the legend of Figure 4b marks f=5 Hz. Please carefully check and correct this typo to ensure the consistency of all key parameters throughout the manuscript.

Reply:

Thank you for correcting the typo. The parameters described in the Methods are correct, and we have revised Fig. 4 with its caption as follows.

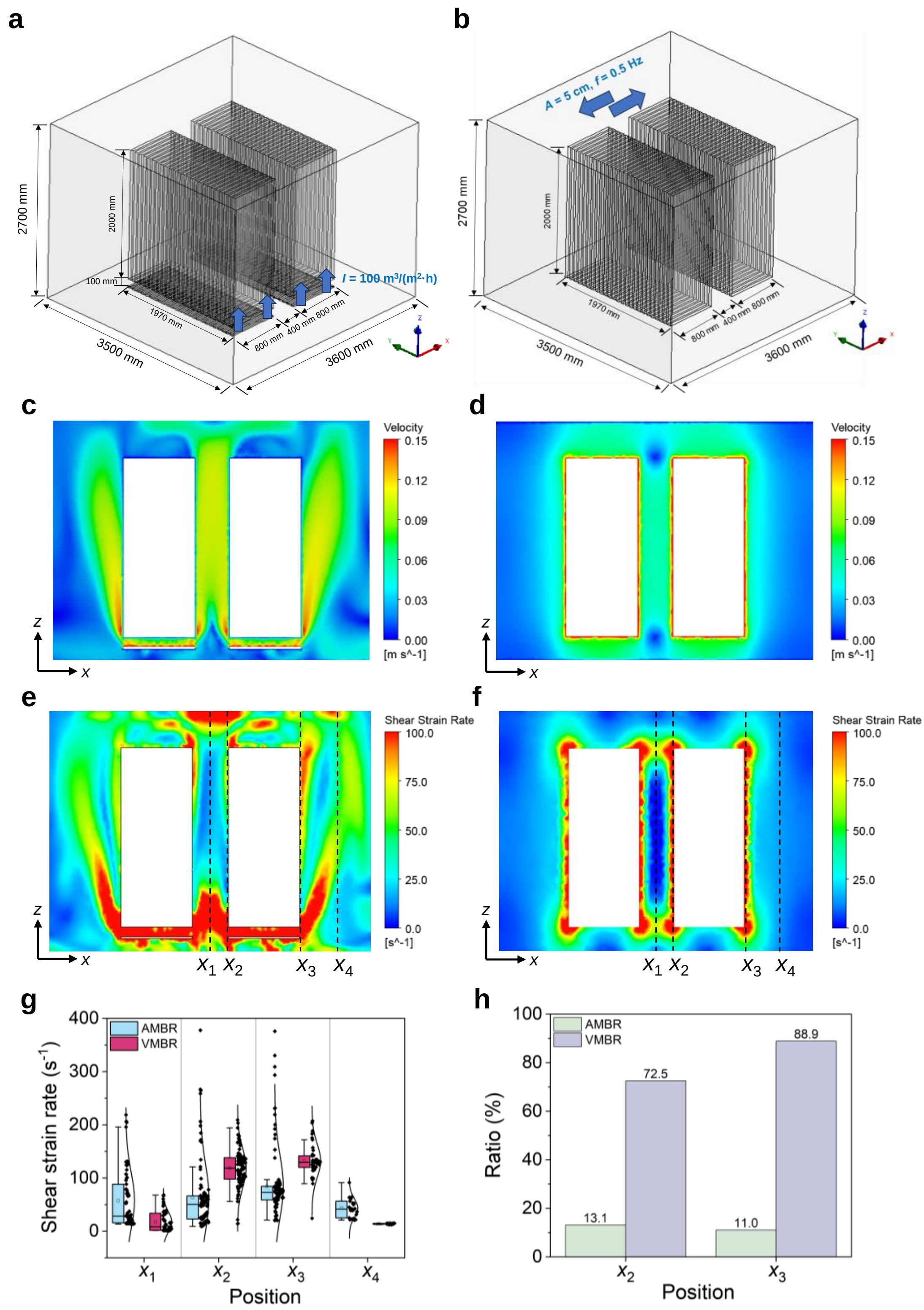

Fig. 4 Comparison of flow field characteristics in the AMBR and VMBR membrane tanks. a–b, Physical models of the membrane tanks of the (**a**) AMBR and (**b**) VMBR. Each membrane tank has 2 stacks of membrane sheets, with 25 sheets per stack and a spacing of 30 mm between adjacent membrane sheets. In the AMBR, an aeration box is installed directly beneath each membrane sheet, with an aeration intensity of 100 $m^3/(m^2 \cdot h)$. In the VMBR, instead of the aeration boxes, all the membrane sheets undergo horizontal reciprocating vibration with an amplitude (A) of 5 cm and a

frequency (*f*) of 0.5 Hz. Other key geometric parameters are indicated in the figures. **c–d,** Velocity distribution of mixed liquor in the vertical cross section of the (**c**) AMBR and (**d**) VMBR. **e–f,** Fluid shear rate distribution in the vertical cross section of the (**e**) AMBR and (**f**) VMBR. **g,** Statistical distribution of fluid shear rates on the *x*-axis for the AMBR and VMBR. **h,** Comparison of the area percentage on the membrane surface where the fluid shear rate exceeds 100 s^{-1} between the AMBR and VMBR. Here, $x_1 = 0$ (the mixed liquor region at the midpoint between the two stacks of membrane sheets), $x_2 = 0.2$ m and $x_3 = 1$ m (regions near the membrane surface), and $x_4 = 1.4$ m (the mixed liquor region at the midpoint between the membrane stack and the outer boundary).

10. Line 520: Add the related reference to the measurement method.

Reply:

Thank you for this comment. We have added the following reference to the measurement method described here.

*39. Magic-Knezev A, van der Kooij D. Optimisation and significance of ATP analysis for measuring active biomass in granular activated carbon filters used in water treatment. Water Research **38**, 3971-3979 (2004).*

11. Line 563: What indicator was used for the grid independence test?

Reply:

Thank you for this comment. The average shear stress rate was used as the indicator for the grid independence test. We have supplemented this information in the Methods section, as "A grid independence study was performed using average shear strain rate as the key indicator, with a relative difference of less than 10% between the optimized mesh (~1.84 million elements) and a finer mesh (Supplementary Fig. 22), which meets the CFD accuracy requirement while balancing computational cost." in Line 699-704.

12. Line 625: The parameter "BOD" appears for the first time in the main text without defining its full form.

Reply:

Thank you for the correction. We have provided the full form of BOD here, as "a maximum methane-producing capacity of 0.6 kg CH_4/kg biochemical oxygen demand (BOD) with a methane correction factor of 0.165 for CH_4) for both the AMBR and VMBR systems." in Line 765-767.

-Reviewer 2

This study shows strong potential to advance municipal wastewater treatment. A key strength is the full-scale, parallel-line validation under real wastewater treatment plant conditions, where a long-term VMBR line (7,500 m^3/d) is benchmarked against a conventional AMBR line. The authors report simultaneous improvements in nutrient removal, fouling resistance, and sustainability performance. Overall, the manuscript is potentially impactful. However, the interpretation and substantiation of the claimed innovation, together with several key aspects regarding the process configuration and comparability between the two full-scale lines, require substantial clarification and

strengthening to make the manuscript more rigorous and persuasive.

This is a potentially valuable full-scale engineering study, and I can see a path to publication. However, the manuscript currently overstates both novelty and mechanistic certainty. Therefore, I recommend major revision before the manuscript can be considered for publication. Please see the specific comments below:

Reply:

We sincerely appreciate the reviewer's positive evaluation and insightful suggestions on our work. We agree that the novelty and mechanistic certainty should be further clarified to make the work more rigorous. The detailed response and revisions based on your questions and suggestions are listed below. The line numbers in the response refer to the revised manuscript with changes marked.

1. The manuscript presents the two full-scale lines as parallel systems differing mainly in membrane-tank mixing (aeration in AMBR vs vibration in VMBR). However, Fig. 1 shows different recycle ratios (200% in VMBR vs 500% in AMBR), which the authors attribute to the lower DO in the VMBR membrane tank. While this may be acceptable in practice, it introduces a key confounding factor for a "parallel-line" comparison. Such differences in recycle strategy and DO conditions will inevitably influence overall treatment performance, making it unclear whether the reported advantages are mainly due to vibration itself or due to the altered DO/recycle regime enabled by VMBR. The authors should clarify this point and define the innovation boundary more explicitly.

Reply:

We appreciate the reviewer for raising this rigorous, insightful, and highly constructive comment, which is critical for clarifying the innovation boundary of our work. Below, we provide a detailed clarification of the issue and corresponding revisions to the manuscript.

(1) The differences in recycle strategy and DO conditions are endogenous outcomes of the fouling control technology, not independent confounding variables.

For the conventional AMBR, continuous aeration is mandatory for fouling control, which inevitably creates a high-DO environment (7.1 ± 0.9 mg/L) in the membrane tank. Direct recycling of this oxygen-rich mixed liquor to the anaerobic tank would break the strict anaerobic environment required for phosphorus release and deplete high-quality volatile fatty acid (VFA) carbon sources. Thus, the AMBR must adopt a multi-step recycle pathway to avoid direct oxygen input into the anaerobic zone. However, this multi-step pathway inherently dilutes sludge concentration during the long recycle loop. To maintain the target mixed liquor suspended solids (MLSS) concentration and sludge retention time (SRT) in the anaerobic tank, the AMBR consequently requires a much higher total recycle ratio, which is an inherent technical limitation of aeration-based fouling control.

For the VMBR, mechanical vibration fully replaces aeration for fouling control, so the membrane tank naturally maintains an anoxic environment (DO < 0.1 mg/L) without additional intervention. This allows direct recycling of mixed liquor to the anaerobic tank without disrupting the anaerobic environment, enabling a lower recycle

ratio to meet process requirements. This optimized recycle regime is an endogenous advantage unlocked by the vibration technology, not a separate process modification.

All other key operational parameters of the biological treatment train (e.g., HRT, aerobic zone aeration intensity, etc.) were kept identical between the two systems, ensuring a controlled, representative comparison of full-scale engineering performance under each technology's optimal operating conditions.

We acknowledge the lack of a detailed elaboration on this issue in our original manuscript, and for this reason, we have added the following paragraph to the description of the AMBR and VMBR systems in the Methods section, as "The recycle strategy for each system was designed following full-scale engineering optimal principles, dictated by the inherent DO characteristics of the membrane tank from each fouling control method. For the AMBR, the high-DO environment in the aerated membrane tank required a multi-step recycle pathway (membrane tank → aerobic tank → anaerobic/anoxic tanks) to avoid disrupting the anaerobic environment, with a total recycle ratio of 500% to meet sludge retention and nitrogen removal requirements. For the VMBR, the vibration-enabled anoxic membrane tank (DO < 0.1 mg/L) allowed direct recirculation of mixed liquor to the anaerobic tank without oxygen interference, enabling a reduced recycle ratio of 200% while maintaining stable process performance. All other key operational parameters of the biological treatment train, including hydraulic retention time (HRT) and aeration intensity of the aerobic tank, were identical between the two parallel systems to ensure a controlled and rigorous comparison." in Line 568-580.

(2) The innovation boundary of this work has been explicitly defined in the Results and Discussion sections.

We fully recognize that the vibration technology, the resulting anoxic DO environment in the membrane tank, and the adapted recycle strategy are inherently linked and cannot be completely separated. To address the reviewer's concern, we clarify the mechanistic origin of each observed performance advantage, distinguishing between the inherent benefits of the vibration technology itself and the synergistic gains unlocked by this technology, to explicitly define the innovation boundary of this work.

Firstly, the 20% higher specific flux and 75% reduction in specific energy consumption for fouling control are core advantages derived directly from the vibration-based fouling control mechanism, independent of the system's DO environment and recycle configuration. We have strengthened this point at the end of the analysis of antifouling mechanism, as "These results demonstrate that the improved antifouling performance of the VMBR is directly driven by the targeted, uniform shear generated by mechanical vibration, an inherent hydrodynamic feature of the technology that is independent of the system's DO concentration and recycle strategy." in Line 428-432.

Secondly, the 22% reduction in effluent TN and 40% lower chemical dosage for phosphorus removal are synergistic benefits enabled by the vibration technology. A net 3.8 mg/L TN removal advantage from the membrane tank alone is directly attributed to sustained endogenous denitrification in the anoxic VMBR membrane tank, which is only possible because vibration eliminates the need for membrane tank aeration. The

remaining TN removal benefit and reduced chemical dosage stem from the optimized recycle regime, which preserves influent VFA carbon sources and enriches PAOs and DPAOs to enhance biological nutrient removal. Critically, this process optimization cannot be replicated in the conventional AMBR, as the oxygen-rich mixed liquor from the aerated membrane tank will inevitably introduce dissolved oxygen into the anaerobic zone, even with multi-step recycle designs. This inherent oxygen intrusion disrupts the strict anaerobic conditions required for efficient biological nutrient removal, and cannot be eliminated by adjusting the recycle ratio alone. We have further clarified this point, as "Notably, the differences in DO environment and recycle strategy between the two systems are not independent variables, but endogenous outcomes of the distinct fouling control technologies. The anoxic environment in the VMBR membrane tank directly contributed a 3.8 mg/L TN removal advantage via sustained endogenous denitrification, while the optimized recycle regime enabled by this anoxic environment accounted for the remaining TN removal benefit and 40% reduction in phosphorus removal chemical dosage." in Line 163-169.

2. To rigorously support the claimed innovation, an ideal experimental design would include a true control in which the VMBR line is operated with vibration turned off (i.e., identical configuration and operating conditions except for vibration), so that the net contribution of vibration can be directly quantified. Although this may be difficult to implement at full scale for practical reasons, the authors are strongly encouraged to supplement the study with a bench-/pilot-scale control test using the same wastewater (or representative mixed liquor) and comparable operating conditions.

Reply:

We thank the reviewer for this suggestion. Below, we clarify the rationale for our experimental design, along with corresponding manuscript revisions.

First, it is critical to clarify that a submerged MBR system with no fouling control measures (i.e., vibration turned off with no supplementary aeration) is neither technically feasible nor scientifically meaningful for this study, which is a well-established consensus in MBR research and engineering practice. Without continuous and effective fouling control via either aeration scouring or mechanical vibration, submerged membrane modules treating municipal wastewater will undergo rapid, severe, and irreversible fouling, with TMP rising to the operational limit within hours to days. Such a setup cannot sustain stable filtration and basic wastewater treatment function, and thus cannot generate valid and long-term comparative data to quantify the contribution of vibration.

For this reason, the conventional AMBR, which is the current dominant, commercially mature technology for full-scale MBR applications worldwide, is universally recognized as the gold-standard benchmark control for evaluating innovative MBR fouling control technologies in both academic research and engineering practice. This benchmarking approach has been widely adopted in peer-reviewed studies on vibration/reciprocation-based MBR technologies, including the prior lab- and pilot-scale investigations cited in our manuscript. In our study, the two parallel full-scale lines share identical front-end anaerobic-anoxic-aerobic biological

treatment trains, influent wastewater quality, HRT, and organic loading rate, *etc*. The only fundamental difference between the two systems is the membrane fouling control strategy: continuous aeration scouring for the AMBR, and mechanical vibration for the VMBR. This head-to-head parallel design is the most rigorous, representative, and industry-accepted comparison that can be implemented at full scale, as it directly compares the innovative technology against the current industry standard under real-world operational conditions.

To clarify this issue, we have added the statement "All other key operational parameters of the biological treatment train, including hydraulic retention time (HRT) and aeration intensity of the aerobic tank, were identical between the two parallel systems to ensure a controlled and rigorous comparison. This head-to-head comparison against the industry-standard conventional AMBR aligns with established MBR research and engineering consensus, as a system without any fouling control measures cannot sustain stable filtration and thus cannot serve as a valid experimental control." in Line 577-583.

3. The manuscript reports an approximately 40% reduction in chemical dosage while achieving comparable TP, which is potentially a strong and practically relevant finding. However, the current explanation is not sufficiently clear and convincing. The authors attribute the lower chemical demand to a much higher abundance of PAOs in VMBR than in AMBR (Page 9), but the manuscript does not adequately clarify what drives this PAO enrichment under the VMBR configuration (e.g., vibration-induced effects versus differences in aeration/DO regime and/or recycle strategy). It does not demonstrate enhanced activity, flux, or pathway dominance. For a mechanistic claim at this level, I would expect stronger functional evidence such as targeted gene quantification, activity assays, phosphorus release/uptake kinetics under controlled conditions, or process-model-based mass-balance support.

Reply:

Thank you for the insightful and constructive comment, which is critical for strengthening the mechanistic explanation. We address the comment mainly through analysis of biological activity and phosphorus release/uptake kinetics, with corresponding targeted revisions to the manuscript, as shown below.

We explicitly clarify that the significant enrichment of PAOs and DPAOs in the VMBR is not driven by a direct physical effect of vibration, but by the stable, strictly anaerobic metabolic niche in the front-end anaerobic tank, a unique advantage fundamentally and endogenously enabled by the vibration-based fouling control technology. For the conventional AMBR, mandatory continuous aeration in the membrane tank for fouling control creates a high-DO environment (7.1 ± 0.9 mg/L), leading to high nitrate and DO carryover into the anaerobic tank via recycled mixed liquor. This results in 6.7 mg/L of nitrate removal in the AMBR anaerobic tank, which consumes high-quality influent volatile fatty acids (VFAs) via denitrification, disrupts the strictly anaerobic environment required for PAO metabolism. In contrast, the VMBR maintains an anoxic membrane tank (DO < 0.1 mg/L) with no DO or excess nitrate carryover. This limits nitrate removal in the anaerobic tank to only 3.0 mg/L,

preserving influent VFAs for PAO intracellular carbon storage and maintaining the strict anaerobic environment — the core prerequisites for PAO/DPAO enrichment. The significant anaerobic phosphorus release of 8 mg/L observed in the VMBR anaerobic tank directly reflects the robust metabolic activity of these enriched functional populations. We have re-written the related discussion to strengthen the mechanistic explanation, as "Analysis of TP removal pathways revealed different behaviors between the two systems (Supplementary Fig. 4). For the AMBR, membrane tank aeration caused high nitrate/DO carryover into the anaerobic tank, leading to 6.7 mg/L of nitrate removal that depleted VFAs and disrupted the anaerobic environment required for phosphate-accumulating organisms (PAOs) metabolism (Supplementary Fig. 3). In contrast, vibration-based fouling control in the VMBR maintained an anoxic membrane tank (DO < 0.1 mg/L), limiting anaerobic nitrate removal to 3.0 mg/L, preserving VFAs and sustaining the strict anaerobic conditions prerequisite for the enrichment of PAOs and denitrifying PAOs (DPAOs). The VMBR system resulted in significant anaerobic phosphorus release (8 mg/L), indicating robust activity of these enriched populations, followed by substantial biological uptake in the preanoxic and aerobic zones (4.9 mg/L and 6.4 mg/L, respectively), while negligible biological phosphorus removal in the AMBR led to near-total reliance on chemical precipitation (Supplementary Fig. 4)." in Line 170-183.

4. The Methods section lacks sufficient description of the microbial community detection workflow and the associated bioinformatics/statistical analyses, which limits reproducibility and weakens the microbiology-based interpretation.

Reply:

We acknowledge the lack of sufficient methodological details for microbial detection and associated bioinformatics/statistical analyses in the original manuscript. To fully address this concern, we have supplemented a complete, concise description of the microbial community analysis in the Methods section, as "Mixed liquor samples for microbial community analysis were collected in triplicate from the membrane tanks of both the AMBR and VMBR systems, with consistent sampling volume and location to ensure comparability. After collection, the samples were placed on ice, immediately transported to the laboratory, and centrifuged to harvest biomass pellets, which were aliquoted into sterile centrifuge tubes and stored at -80 °C until analysis. Total genomic DNA was extracted from the pellets using a commercial kit, with DNA concentration and integrity verified via spectrophotometry and 1% agarose gel electrophoresis. The V4 hypervariable region of the bacterial 16S rRNA gene was amplified using the barcoded universal primer pair 515F/806R, with purified PCR amplicons pooled into equimolar libraries and sequenced on the Illumina NovaSeq 6000 platform. Raw sequencing data were processed using QIIME 2 (v2023.5), including quality filtering, denoising to generate amplicon sequence variants, and taxonomic classification against the SILVA 138.1 database, with statistically significant differences in genus-level relative abundance between the two systems assessed via two-tailed Student's t-test (significance threshold $p < 0.05$)." in Line 669-683.

5. In the Methods, membrane fiber samples were collected only from the periphery of the AMBR and VMBR modules. Meanwhile, the CFD results suggest spatially non-uniform hydrodynamics, particularly in AMBR, which may translate into non-uniform fouling within the module. Given that fouling heterogeneity is commonly observed in full-scale systems, restricting sampling to peripheral fibers may introduce location bias and lead to position-dependent conclusions. The authors are encouraged to adopt a more representative sampling design by stratifying samples by module height (top/middle/bottom) and bundle position (periphery vs center), with appropriate replicates, and to compare fouling quantity/composition and filtration resistance accordingly.

In addition, organic foulants were eluted using 0.5% NaClO, which is an aggressive oxidant and can alter proteins, polysaccharides, and humic-like material. This makes the quantitative compositional comparison less convincing, as the cleaning efficiency by using 0.5% NaClO could be largely different between AMBR and VMBR. These limitations should be acknowledged explicitly, and the claims about foulant composition should be moderated unless the authors can provide validation that the extraction approach preserves the analytes of interest.

Reply:

Thank you for the insightful suggestion.

First, we clarify the rationale of our original sampling design and the objective constraints on supplementary testing. The membrane fouling samples in this study were collected at three fixed time points (day 30, 60, and 90) during the first operation cycle after the first offline chemical cleaning, corresponding to specific fouling development stages of the two systems. At present, the membrane modules have been in continuous long-term operation, and the current fouling state is completely inconsistent with the specific stages during the controlled comparison period. Supplementary sampling and testing now cannot reflect the original fouling characteristics during the experiment, and the resulting data would not be comparable with our existing results. For the original sampling design, we collected membrane fiber samples from the identical peripheral position of both AMBR and VMBR membrane modules, with fully consistent sampling protocols, sample volume, and pretreatment methods for the two systems. This design was selected to minimize disturbance to the structural integrity and continuous stable operation of the full-scale membrane modules, as sampling from the module center would require partial disassembly of the membrane stack and risk damaging the module sealing structure. More importantly, the consistent sampling location across the two parallel systems ensures the comparability of fouling characterization results between AMBR and VMBR, and our core conclusion that VMBR exhibits superior antifouling performance is not affected by the sampling location.

Second, we fully recognize the scientific value of the stratified sampling design suggested by the reviewer, and we have systematically completed this work in another study. In that study, we conducted a comprehensive investigation of the spatial heterogeneity of membrane fouling in an engineering-scale VMBR system, with different module heights (top/middle/bottom) and different bundle positions (periphery

vs center). We performed a systematic comparison of fouling quantity, foulant composition, and filtration resistance across all sampling points, and the related manuscript is currently under peer review at another journal. To avoid duplicate publication of overlapping research content, these results are not included in the present manuscript.

Third, although the 0.5% NaClO elution method is widely used for membrane foulant extraction, we recognize the limitation that NaClO's oxidizing nature may partially alter the structures of organic foulants, and differences in fouling layer properties may lead to slight variations in elution efficiency. Accordingly, we have moderated the relevant claims about foulant composition to ensure the prudence of our conclusions, as "Furthermore, the total organic carbon (TOC) and adenosine triphosphate (ATP) levels were consistently higher in the AMBR after the initial stage (Supplementary Fig. 9a–b), reflecting relatively more severe organic and biological fouling." in Line 260-263, "Analysis of organic foulant composition based on the NaClO elution method revealed a temporal shift from proteins and polysaccharides in the initial fouling layer (M1) toward a higher proportion of recalcitrant humic acids in later stages (M2 and M3) (Supplementary Fig. 9c–e)." in Line 264-267, and "Quantitative analysis and excitation–emission matrix (EEM) fluorescence spectra collectively indicated a higher degree of organic fouling in the AMBR system relative to the VMBR system (Supplementary Fig. 10)." in Line 269-272,

6. The Methods describe the VMBR vibration conditions as 0.5 Hz with an amplitude of 5 cm, whereas the caption/text associated with Fig. 4 states 5 Hz and 50 mm. The authors should carefully re-check and verify the correct operating parameters and ensure consistency across the Methods, figures/captions, and any related analyses (e.g., CFD and energy calculations).

Reply:

Thank you for correcting the typo. The parameters described in the Methods are correct, and we have revised Fig.4 with its caption as follows.

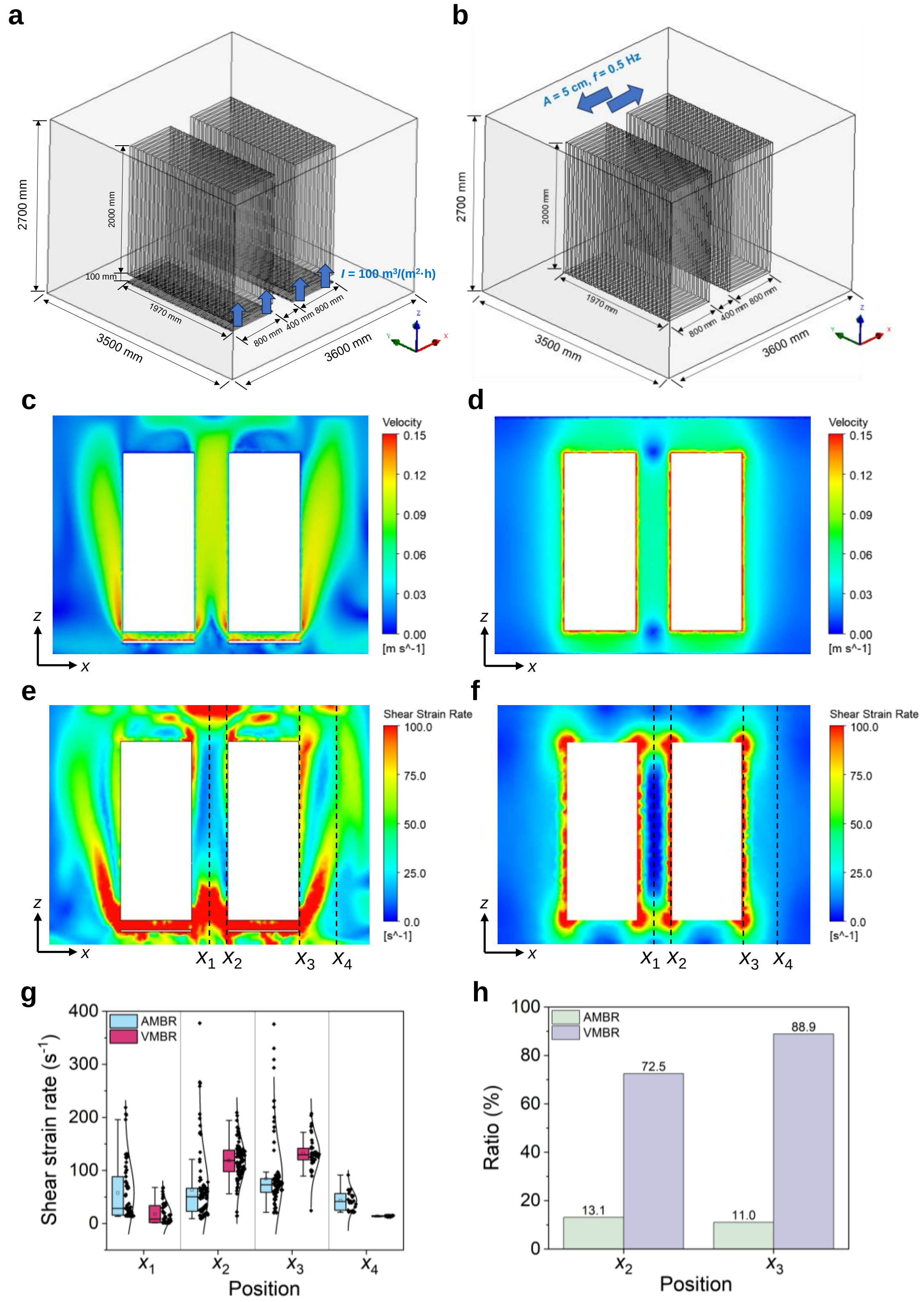

Fig. 4 Comparison of flow field characteristics in the AMBR and VMBR membrane tanks. a–b, Physical models of the membrane tanks of the (**a**) AMBR and (**b**) VMBR. Each membrane tank has 2 stacks of membrane sheets, with 25 sheets per stack and a spacing of 30 mm between adjacent membrane sheets. In the AMBR, an aeration box is installed directly beneath each membrane sheet, with an aeration intensity of 100 $m^3/(m^2{\cdot}h)$. In the VMBR, instead of the aeration boxes, all the membrane sheets undergo horizontal reciprocating vibration with an amplitude (*A*) of 5 cm and a

frequency (*f*) of 0.5 Hz. Other key geometric parameters are indicated in the figures. **c–d,** Velocity distribution of mixed liquor in the vertical cross section of the (**c**) AMBR and (**d**) VMBR. **e–f,** Fluid shear rate distribution in the vertical cross section of the (**e**) AMBR and (**f**) VMBR. **g,** Statistical distribution of fluid shear rates on the *x*-axis for the AMBR and VMBR. **h,** Comparison of the area percentage on the membrane surface where the fluid shear rate exceeds 100 s^{-1} between the AMBR and VMBR. Here, $x_1 = 0$ (the mixed liquor region at the midpoint between the two stacks of membrane sheets), $x_2 = 0.2$ m and $x_3 = 1$ m (regions near the membrane surface), and $x_4 = 1.4$ m (the mixed liquor region at the midpoint between the membrane stack and the outer boundary).

7. In the CFD model, the mixed liquor was assigned a density of 1000 kg/m^3 and a dynamic viscosity of 0.001 Pa·s, essentially treating it as water. However, the viscosity of MBR mixed liquor is typically substantially higher than that of water and can vary with MLSS and shear rate. Using a water-like viscosity may bias the predicted flow field and shear distribution. The authors should incorporate measured (or otherwise well-justified) mixed liquor viscosity under the relevant operating conditions and discuss how this assumption affects the CFD-based conclusions.

Reply:

Thank you for this constructive comment. We note that our CFD analysis focused primarily on the relative differences in shear distribution between the AMBR and VMBR systems, for which we applied a consistent mixed liquor viscosity corresponding to the initial operation stage. The measured viscosity data for both membrane tanks are provided in Supplementary Figure 12, showing a value of approximately 1 cP (0.001 Pa·s) at the early stable operation phase for both systems. To further improve the rigor and transparency of the model description, we have made the following revisions to the Methods section, as "The mixed liquor was assumed to be perfectly mixed within the reactor and defined as the continuous phase, with a density of 1005 kg/m^3 and a dynamic viscosity of 0.001 Pa·s, matching the measured value at the system's initial stable operation stage (Supplementary Fig. 12)." in Line 708-711.

8. The Methods state that an online NaClO cleaning was conducted on a weekly basis. The authors should clearly annotate the cleaning times on the TMP and flux profiles, so that the post-cleaning recovery and cleaning effectiveness can be directly assessed and compared between AMBR and VMBR.

Reply:

Thank you for this suggestion. In response to the subsequent suggestion (comment 9#), we have compiled more than two years of continuous operational data and reconstructed the profiles of permeate flux and TMP. Given the long monitoring period and high frequency of weekly online cleaning, it is impractical to label every individual online cleaning event clearly in the figure. Instead, we have marked all offline chemical cleaning events (approximately once every six months) with vertical dashed lines, as shown below in the revised Fig. 2. These key offline cleaning points allow direct evaluation and comparison of post-cleaning performance recovery and cleaning efficiency between the AMBR and VMBR.

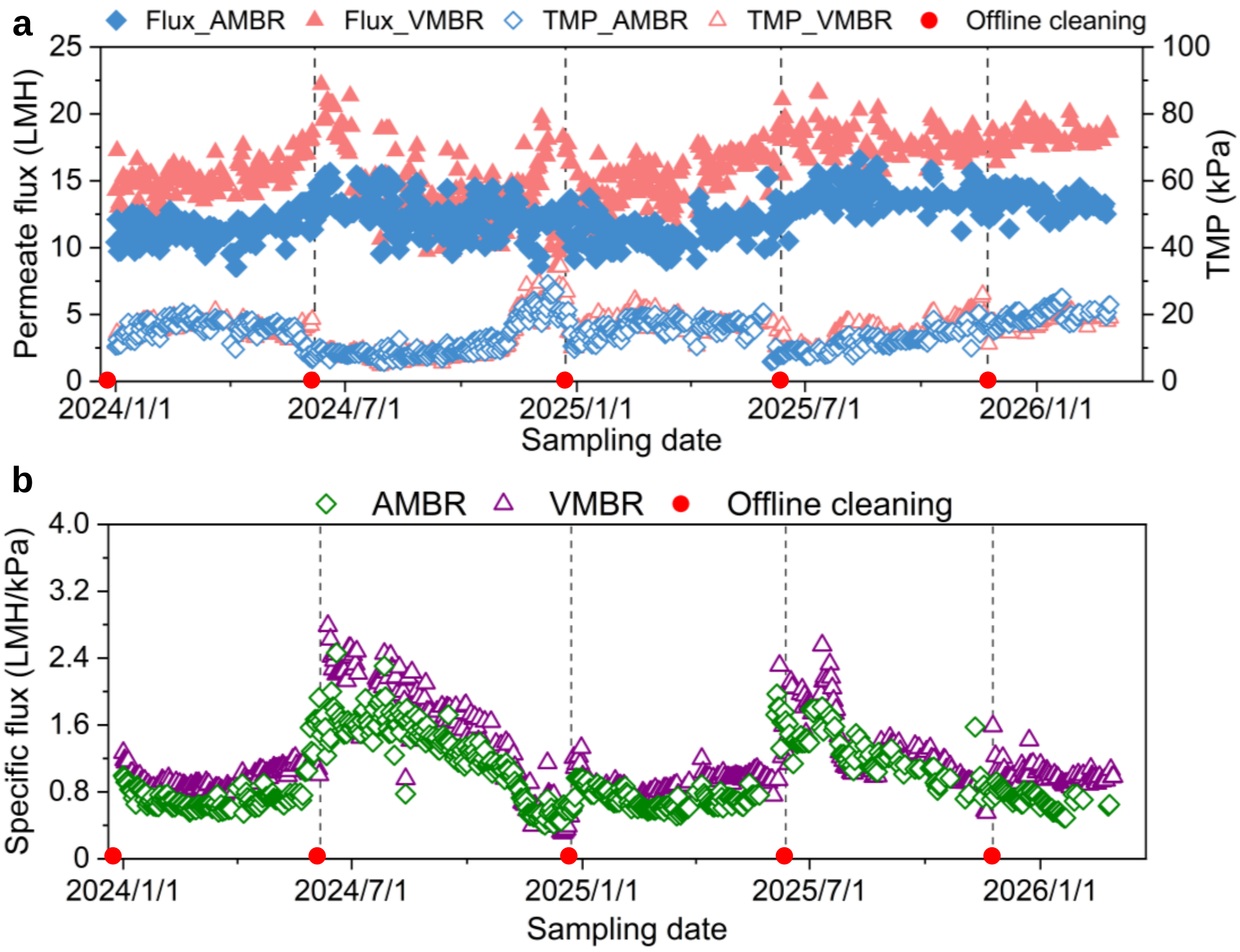

Fig. 2 Comparison of operational stability between AMBR and VMBR over a ~800-day operational period. a, Temporal variations of permeate flux and transmembrane pressure (TMP) over more than two years of operation. **b,** Temporal variation of specific flux (ratio of permeate flux to TMP) over more than two years of operation. Vertical dashed lines indicate offline cleaning events for both systems.

9. The Methods state that AMBR and VMBR have been stably operated for ~4 years, but the manuscript mainly presents a much shorter monitoring period. The authors should consider including longer-term performance/stability data, which would be highly valuable for evaluating the practical engineering applicability of VMBR.

Reply:

We sincerely thank the reviewer for this valuable and constructive suggestion. Including long-term full-scale operational data is critical to demonstrating the practical engineering applicability of the VMBR technology, and it also greatly strengthens the robustness of our conclusions and the overall impact of this work.

The full-scale systems were commissioned in 2021, and were forced to suspend operation in July 2023 due to severe damage to the host wastewater treatment plant's infrastructure caused by the catastrophic torrential rainstorm and flood disaster in Fangshan District, Beijing. After comprehensive post-disaster restoration, equipment re-commissioning and full process stabilization, the parallel systems resumed continuous and stable operation at the end of 2023.

In response to the reviewer's suggestion, we have re-collected continuous full-cycle operational data from January 1, 2024 to the present (over two full years of monitoring), including core performance indicators such as effluent pollutant removal efficiency,

permeate flux, and transmembrane pressure (TMP). Based on these long-term data, we have completely redrawn Fig. 1 (effluent quality performance) and Fig. 2 (long-term operational stability of flux and TMP) as below, and clarified that the membrane fouling characterization samples M1 (30 d), M2 (60 d), and M3 (90 d) were collected at 30, 60, and 90 days after the first offline cleaning event marked in the revised Fig. 2. We have also comprehensively updated the corresponding descriptions, statistical analysis, and discussion of system performance in the revised manuscript, to fully demonstrate the long-term stable performance and engineering viability of the VMBR technology.

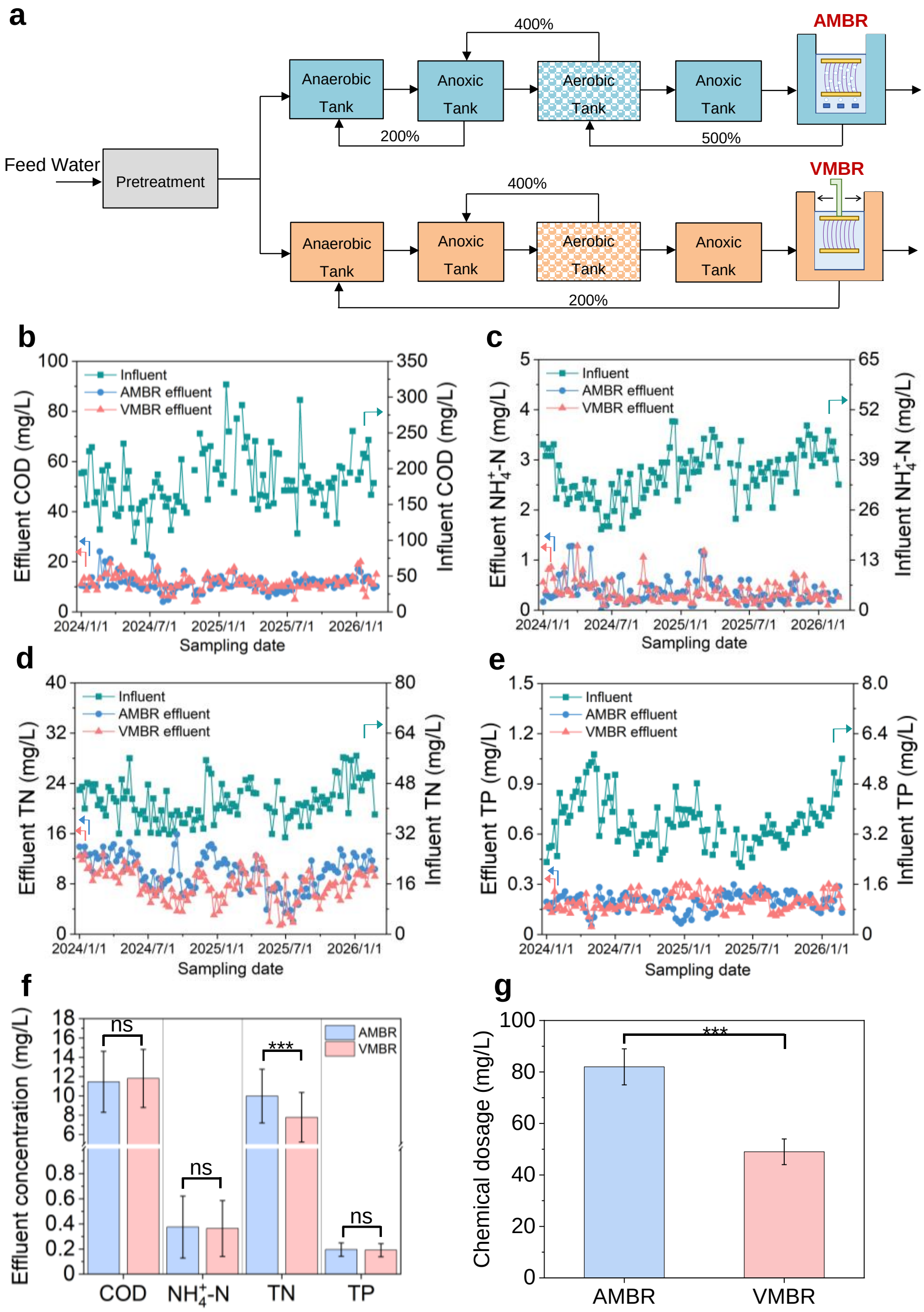

Fig. 1 Process flow diagrams and pollutant removal performance of the aerated membrane bioreactor (AMBR) and the vibrating membrane bioreactor (VMBR). a, Schematic of the AMBR and VMBR processes. **b–e,** Weekly monitoring of influent and effluent quality of municipal

wastewater treated by the AMBR and VMBR over a ~800-day operational period: (**b**) COD, (**c**) NH_4^+-N, (**d**) TN, and (**e**) TP. **f,** Comparison of average effluent quality between the AMBR and VMBR. **g,** Chemical dosage ($FeCl_3$) for phosphorus removal in AMBR and VMBR systems. The error bars denote the standard deviation (SD) ($n \geq 110$), and statistical significance was assessed by a two-tailed Student's t test (*, $p < 0.05$; **, $p < 0.01$; ***, $p < 0.001$; ns, not significant).

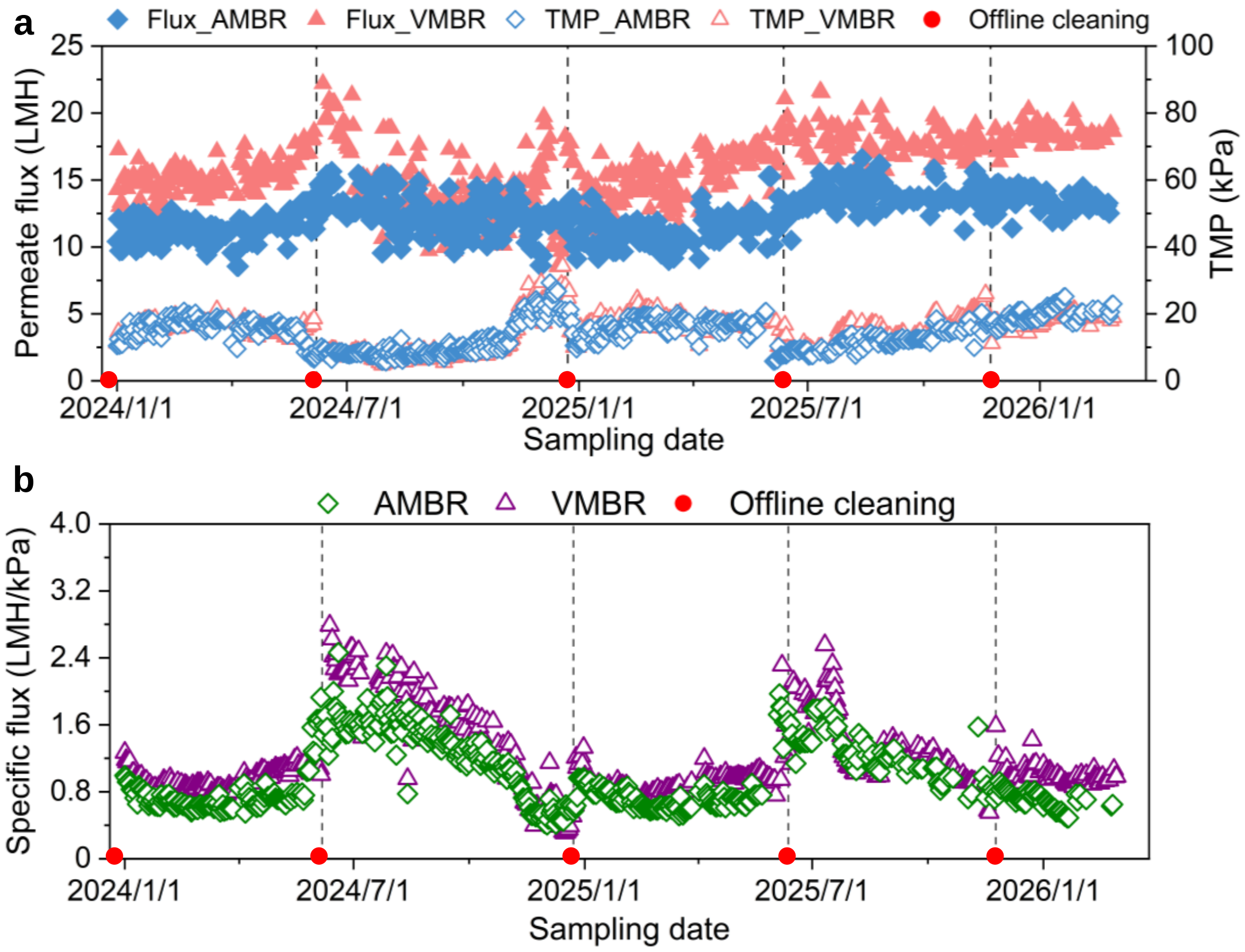

Fig. 2 Comparison of operational stability between AMBR and VMBR over a ~800-day operational period. a, Temporal variations of permeate flux and transmembrane pressure (TMP) over more than two years of operation. **b,** Temporal variation of specific flux (ratio of permeate flux to TMP) over more than two years of operation. Vertical dashed lines indicate offline cleaning events for both systems.

10. The novelty needs to be repositioned much more carefully. The manuscript frames the study as if the benefits of reciprocating/vibrating submerged membranes are being established here for the first time, yet the paper itself cites prior laboratory- and pilot-scale studies already showing improved fouling control, energy savings, and enhanced nutrient removal in reciprocating/vibrating MBRs. The real advance here appears to be the full-scale side-by-side implementation, not the discovery of the core principle. I therefore encourage the authors to substantially tone down the conceptual novelty claim and reframe the manuscript around scale-up validation, practical integration into a full biological train, and limits of transferability.

Reply:

Thank you for this kind suggestion. We fully agree with your opinion that the novelty of this study should be stated more carefully. The primary and novel contribution of our

work is the first comprehensive, long-term and side-by-side validation at full scale (7500 m^3/d), coupled with a deep mechanistic investigation that links system-wide performance to microbial ecology and hydrodynamics within a complete biological treatment train. Following your suggestion, we have thoroughly revised the manuscript to reframe it unambiguously around scale-up validation, practical integration, and mechanistic elucidation, and have toned down all claims of conceptual novelty regarding the VMBR principle itself. The key changes are as follows:

(1) **Abstract:** We have replaced the verb "unveil" with more accurate phrasing. The opening sentence now reads: "Here, we report approximately 800 consecutive days of full-scale (7500 m^3/d) parallel operation of the vibrating MBR (VMBR) and the conventional aerated MBR (AMBR) systems, systematically comparing their pollutant removal performance, long-term filtration stability, membrane fouling mechanisms, and full life cycle environmental impacts." in Line 25-31.

(2) **Introduction:** We have added a new transition sentence after the summary of knowledge gaps, as "Therefore, a definitive, side-by-side demonstration at full scale, within a complete biological treatment train, is critical to confirm these benefits and elucidate the underlying mechanisms governing performance at scale." in Line 90-92.

(3) **Discussion:** We have phrased the opening sentence to tone down the conceptual novelty claim, as "Confirming and extending earlier findings from lab- and pilot-scale studies, this long-term, full-scale comparative study demonstrates that the VMBR project achieves improved performances to the conventional AMBR across critical metrics of sustainability and efficiency." in Line 520-524.

11. The antifouling improvement is promising but should be interpreted more cautiously. The reported average flux difference is relatively modest (15.0 versus 16.1 LMH), and both systems still relied on weekly online cleaning and annual offline cleaning. The lower TMP-rise rate in VMBR is meaningful, but the manuscript presently extrapolates this into a very broad antifouling narrative without sufficient long-term evidence on irreversible resistance buildup, recovery after repeated cleaning cycles, membrane integrity, or actual extension of service life. The antifouling claims would be much stronger if supported by longer post-cleaning trajectories or resistance partitioning.

Reply:

Thank you for this critical, insightful, and highly constructive comment, which guides us to interpret the antifouling performance of VMBR more rigorously and objectively, avoid over-extrapolation of conclusions, and greatly strengthen the robustness of our narrative.

In direct response to this comment and Comment 9#, we have supplemented more than two years of continuous full-scale operational data (from January 2024 to the present), which covers several complete offline cleaning cycles, and completely redrew Fig. 2 to present the full trajectories of permeate flux, TMP, and specific flux before and after each cleaning event. These long-term data demonstrate that the VMBR system achieves better performance recovery after repeated offline cleaning cycles, and maintains higher specific flux for most of the operation period compared with the AMBR, providing solid long-term evidence to support the antifouling advantage of the

vibration-based strategy. Notably, we have updated the average permeate flux data from the original short monitoring period to the full two-year operational dataset in the revised manuscript: the AMBR achieved an average flux of 12.3 ± 1.5 LMH (n = 721), while the VMBR maintained a significantly higher average flux of 15.7 ± 2.3 LMH (n = 664, $p < 0.001$, Supplementary Fig. 6a) and a significantly higher specific flux (n = 663, $p < 0.001$, Supplementary Fig. 6b), with a more pronounced and statistically significant difference than the original short-cycle data.

Accordingly, we have made the following revisions to the manuscript, as "Fig. 2 compares the long-term operational stability of the full-scale AMBR and VMBR systems over more than two years of continuous operation. As illustrated in Fig. 2a, the two systems exhibited similar transmembrane pressure (TMP) variation patterns, with TMP dropping sharply after each offline cleaning event and rising gradually during sustained operation, and both maintained stable TMP levels below 30 kPa throughout the monitoring period. However, the permeate flux of the AMBR was consistently lower than that of the VMBR throughout most of the operation cycles. Over the full monitoring period, the AMBR recorded an average permeate flux of 12.3 ± 1.5 $L \cdot m^{-2} \cdot h^{-1}$ (LMH), while the VMBR maintained a significantly higher average flux of 15.7 ± 2.3 LMH ($p < 0.001$, Supplementary Fig. 6a).

The specific flux, defined as the ratio of permeate flux to TMP (a core metric for membrane permeability and fouling propensity), is presented in Fig. 2b. Over the full monitoring period, the VMBR maintained higher specific flux values for the majority of the operation cycle (Fig. 2b), and achieved an average specific flux 20% higher than the conventional AMBR, with the difference statistically significant ($p < 0.001$, Supplementary Fig. 6b). These results confirm that the vibration-based fouling control strategy delivers superior and more stable long-term filtration performance in full-scale applications. Even under identical offline cleaning regimes, the VMBR preserved better membrane permeability, mitigated fouling progression, and exhibited more robust operational stability than the conventional AMBR." in Line 223-243.

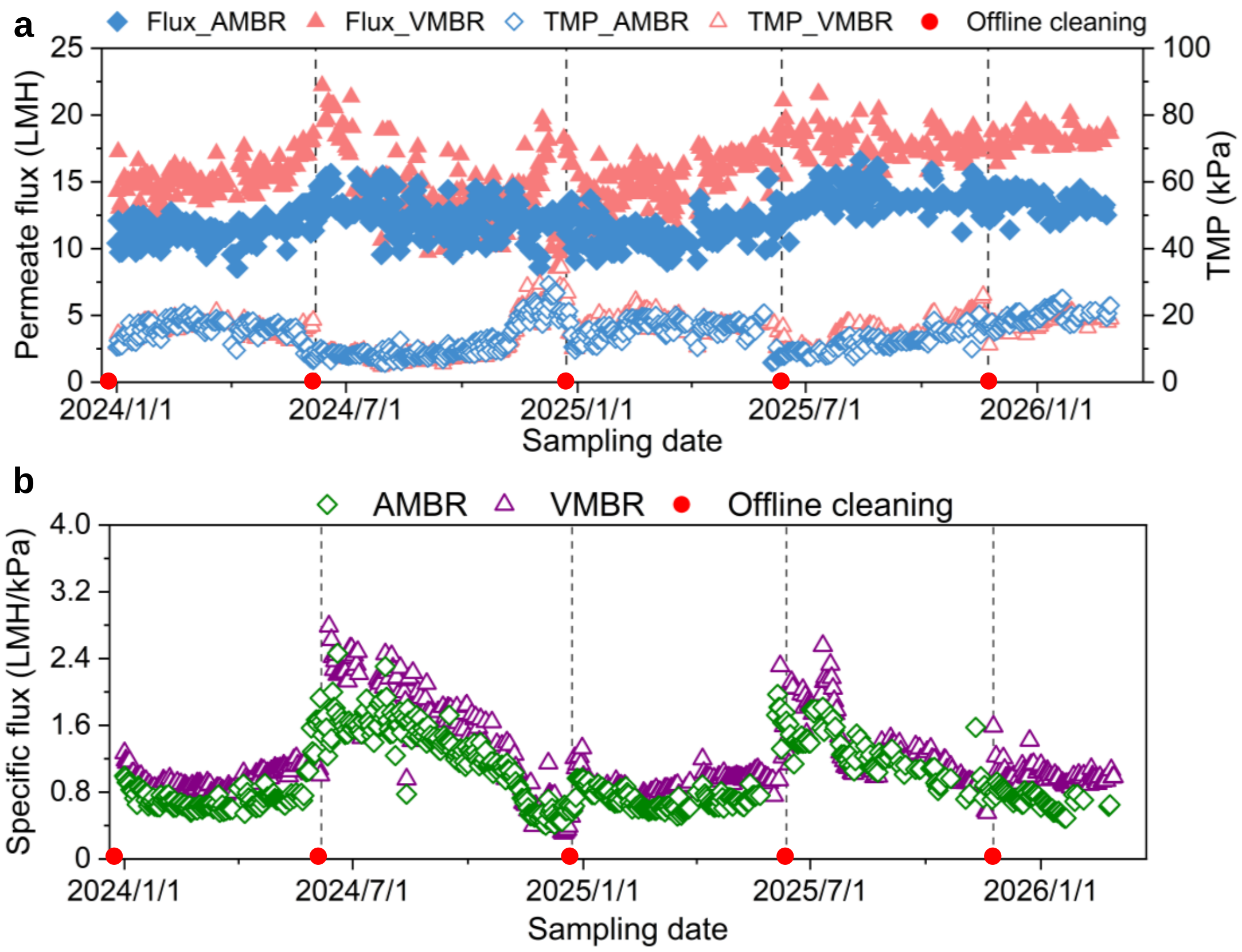

Fig. 2 Comparison of operational stability between AMBR and VMBR over a ~800-day operational period. a, Temporal variations of permeate flux and transmembrane pressure (TMP) over more than two years of operation. **b,** Temporal variation of specific flux (ratio of permeate flux to TMP) over more than two years of operation. Vertical dashed lines indicate offline cleaning events for both systems.

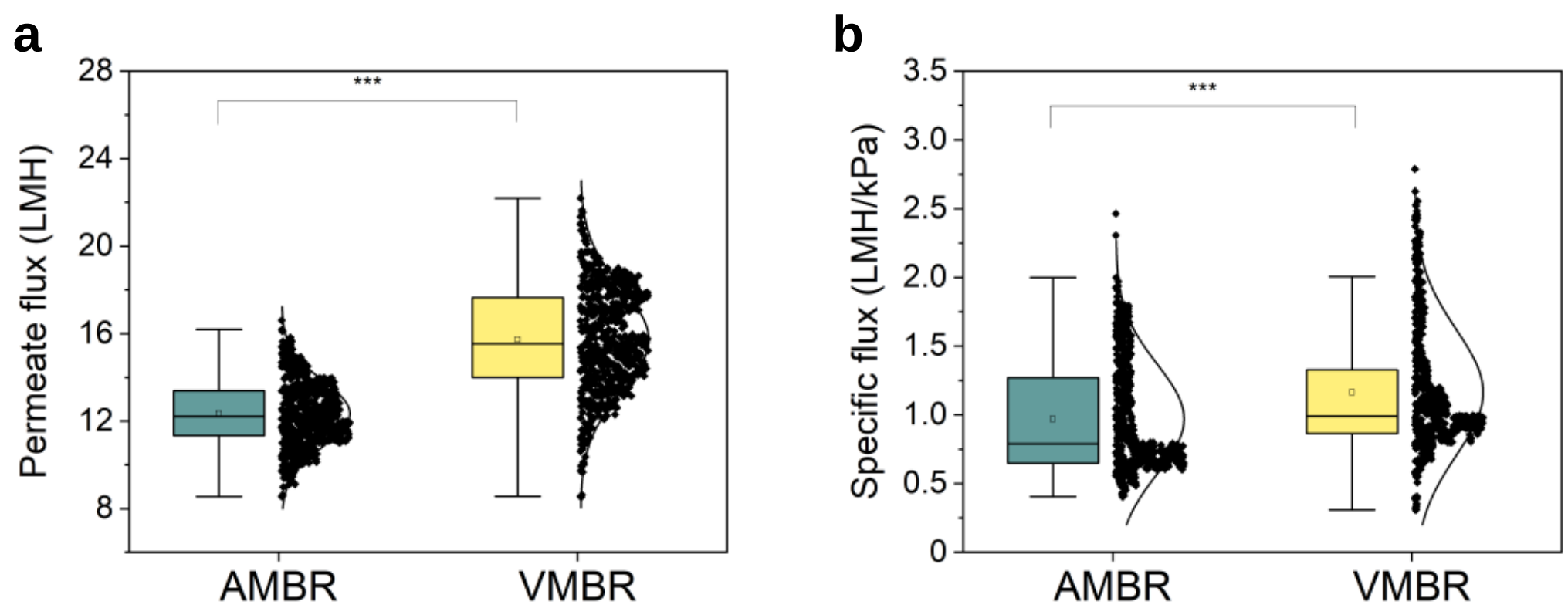

Supplementary Fig. 6 Box-plot comparison of the long-term performance between AMBR and VMBR processes, showing the distributions of (a) permeate flux and (b) specific flux over the ~800-day operational period. Statistical significance of differences in medians was assessed using the nonparametric Mann-Whitney U test (*, $p < 0.05$; **, $p < 0.01$; ***, $p < 0.001$; ns, not significant).

12. The carbon-emission and LCA claims are broader than the underlying evidence base. Emissions seem not directly measured. Rather, N2O and CH4 are estimated from

influent–effluent balances and default emission factors. The LCA then reports relative reductions with the AMBR normalized to 100%, which makes it harder to judge the magnitude and uncertainty of the absolute impacts. Importantly, the paper links lower resource use partly to extended membrane lifespan, but no membrane-life data are actually presented. These sustainability conclusions should therefore be stated more cautiously, and uncertainty analysis would greatly strengthen this section.

Reply:

We sincerely thank the reviewer for this highly constructive comment, which guides us to improve the academic rigor and robustness of this section. We have systematically addressed each of the reviewer's concerns with targeted revisions in the revised manuscript, as detailed below:

(1) Regarding the accounting methodology of N_2O and CH_4 emissions

We acknowledge that the greenhouse gas emissions in this study were estimated via influent-effluent mass balance combined with standardized emission factors, rather than in-situ continuous measurement. This methodology fully complies with the *2019 Refinement to the 2006 IPCC Guidelines for National Greenhouse Gas Inventories*, which is the internationally recognized and widely adopted standard for full-scale wastewater treatment plant carbon emission accounting, and has been extensively applied in peer-reviewed publications. Meanwhile, we fully agree that in-situ monitoring can further improve the accuracy of emission accounting, and we have clearly stated this point in the Discussion, as "Additionally, carbon emission accounting in this study relies on standardized IPCC emission factors, and future on-site continuous monitoring of greenhouse gas emissions would further improve the accuracy of carbon accounting." in Line 543-545. Continuous in-situ measurement of N_2O and CH_4 emissions in the full-scale plant is set as a key direction for our follow-up research.

(2) Regarding the normalization approach of LCA results

We appreciate the reviewer's suggestion on improving the transparency of LCA results. The normalization approach (with AMBR normalized to 100% for each impact category) was adopted mainly because the absolute values of different environmental impact categories vary greatly in orders of magnitude, making it impossible to intuitively compare the relative differences between the two systems in a single figure. This is a widely used and accepted visualization method in LCA-related peer-reviewed publications. In direct response to the reviewer's comment, we have supplemented the full absolute quantitative values of all environmental impact categories for both AMBR and VMBR in Supplementary Table 1 as follows, to allow readers to clearly evaluate the magnitude and absolute impacts of the two technologies.

Supplementary Table 1 Life cycle environmental impacts for the AMBR and VMBR systems.

Category	Reference unit	AMBR	VMBR
Human non-carcinogenic toxicity	CTUh	1.11×10^{-11}	6.14×10^{-12}
Photochemical ozone formation	kg NMVOC eq.	2.04×10^{-6}	1.02×10^{-6}
Freshwater ecotoxicity	CTUe	6.40×10^{-5}	3.20×10^{-5}
Marine eutrophication	kg N eq.	6.58×10^{-9}	4.53×10^{-9}
Terrestrial eutrophication	mol N eq.	7.20×10^{-8}	5.44×10^{-8}

Climate change	kg CO_2 eq.	0.02467	0.02192
Particulate matter formation	disease incidence	1.03×10^{-13}	7.79×10^{-14}
Acidification	mol H^+ eq.	2.50×10^{-8}	1.72×10^{-8}
Resource use, minerals and metals	kg Sb eq.	2.63×10^{-15}	1.57×10^{-15}

(3) Regarding the unsubstantiated claim of extended membrane lifespan

We fully agree with the reviewer's critical point that the original description linking resource use reduction to extended membrane lifespan lacked direct supporting data and was insufficiently rigorous. We have removed the unsubstantiated description related to "extended membrane lifespan" from the original sentence, and revised it to "Furthermore, a 40% reduction in mineral and metal resource use underscores the improved resource efficiency linked to lower chemical demand." in Line 493-495. All resource use-related descriptions in the manuscript are now strictly anchored to our measured operational data, with no overstated claims beyond the evidence base.

(4) Regarding the uncertainty analysis of LCA results

In direct response to the reviewer's valuable suggestion, we have supplemented an uncertainty analysis for the LCA results in the revised manuscript. The analysis was performed using the standard Monte Carlo simulation method with 1000 iterative calculations, and reasonable fluctuation ranges were set for key input parameters (including energy consumption, chemical dosage, and emission factors). The full results of the uncertainty analysis are presented in Supplementary Fig. 21, which shows that the overall result sensitivity of the VMBR is lower than that of the AMBR, indicating that the environmental performance of the VMBR is more stable and less affected by the fluctuation of operational parameters, further supporting the reliability of its environmental benefit conclusion. We have added the related description of the uncertainty analysis method as "Uncertainty analysis was conducted via Monte Carlo simulation (1000 iterations) to assess the robustness of the LCA results." in Line 803-804, and the related discussion as "Uncertainty analysis shows that the VMBR exhibited lower overall sensitivity than the AMBR, indicating more stable environmental performance against operational fluctuations (Supplementary Fig. 21)." in Line 495-497.

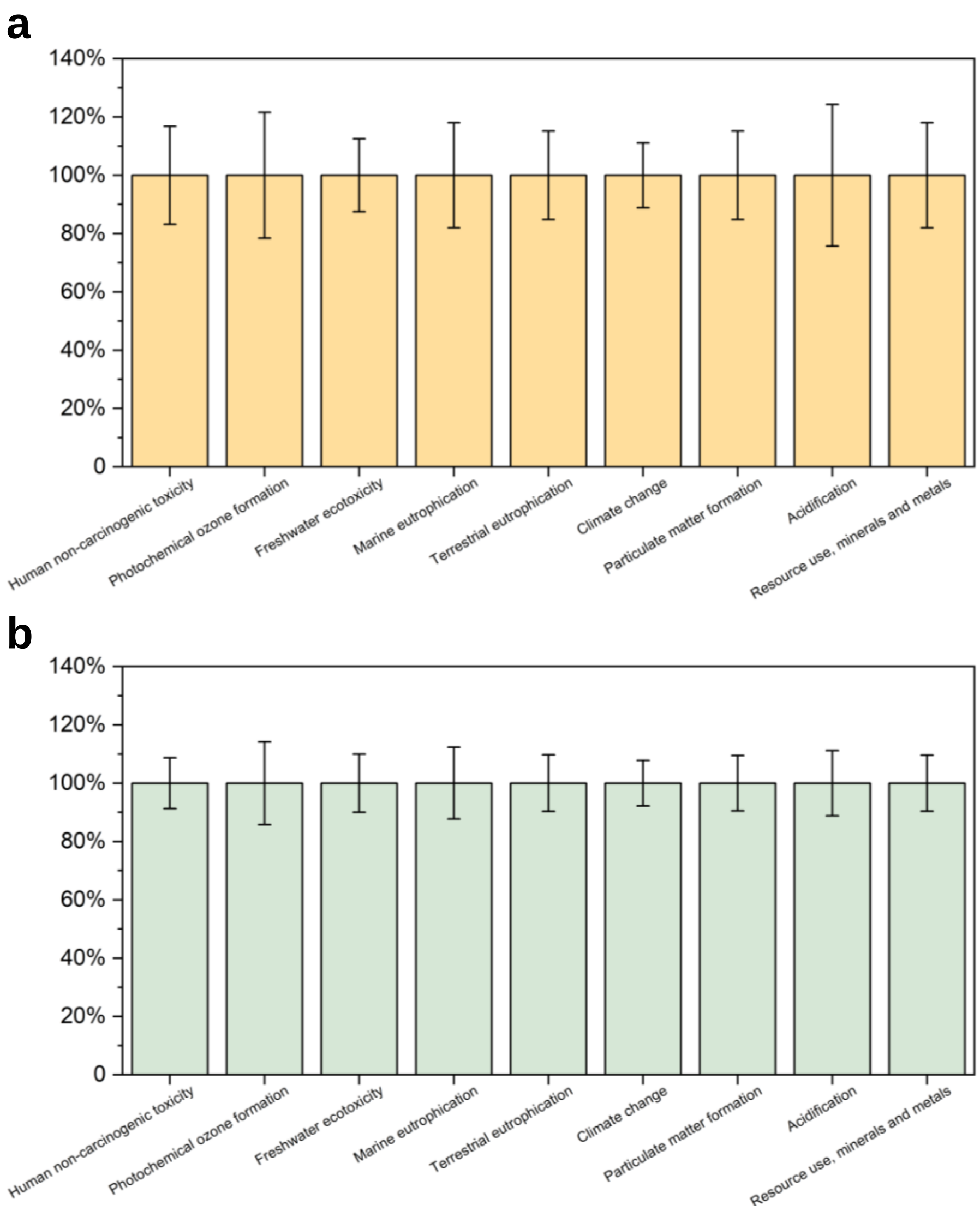

Supplementary Fig. 21 Uncertainty analysis of LCA results of (a) AMBR and (b) VMBR. The error bar represents the uncertainty range (95% confidence interval) of the result.

13. The translational scope remains narrower than the discussion suggests. Although this is full scale, it is still effectively a single-plant case study. The direct comparative window is 120 days after cleaning, within one site configuration and one influent context. The manuscript does not yet adequately address broader deployment questions such as vibration-system reliability, maintenance burden, retrofit feasibility, mechanical durability, or robustness under different wastewater compositions and seasonal regimes. Those issues are especially important if the authors wish to argue that VMBR is a key next-generation wastewater technology.

Reply:

We sincerely thank the reviewer for this rigorous and insightful comment, which is critical for us to objectively position the translational value of this work and strengthen the comprehensiveness of our Discussion section. Firstly, we have extended the comparative monitoring period from 120 days to over two years of stable full-scale operation, greatly strengthening the evidence base according to your suggestion in

Comment 9#. Secondly, we fully agree that systematic evaluation of vibration-system reliability, maintenance burden, retrofit feasibility, mechanical durability, and operational robustness across variable wastewater compositions and seasonal regimes is essential to support the positioning of VMBR as a competitive next-generation wastewater treatment technology. Accordingly, we have comprehensively revised the last paragraph of Discussion section, as "While this full-scale parallel study demonstrates the promising performance of VMBR, broader translational conclusions still require further multi-scenario validation. The full-life-cycle mechanical reliability, maintenance burden, retrofit feasibility for existing MBR facilities, and structural durability of large-scale vibration systems have yet to be comprehensively evaluated over the full membrane design service life (typically 5-8 years). Additionally, carbon emission accounting in this study relies on standardized IPCC emission factors, and future on-site continuous monitoring of greenhouse gas emissions will further improve the accuracy of carbon accounting. Moreover, the operational robustness of VMBR under extreme wastewater quality fluctuations, shock influent loading events, and long-term variable climatic conditions also warrants further investigation. These follow-up studies will further support the wide engineering application of VMBR as a next-generation low-carbon, energy-efficient wastewater treatment technology." in Line 538-550.

-Reviewer 3

I co-reviewed this manuscript with one of the reviewers who provided the listed reports. This is part of the Nature Communications initiative to facilitate training in peer review and to provide appropriate recognition for Early Career Researchers who co-review manuscripts.

Reply:

We appreciate the valuable and constructive comments from all reviewers. We have carefully addressed all comments in our revised manuscript, which greatly helps us improve our work.
